# Gold(I) Complexes Based on Nonsteroidal Anti-Inflammatory Derivatives as Multi-Target Drugs against Colon Cancer

**DOI:** 10.1021/acs.inorgchem.4c02988

**Published:** 2024-10-10

**Authors:** Javier Saez, Javier Quero, María Jesús Rodriguez-Yoldi, M. Concepción Gimeno, Elena Cerrada

**Affiliations:** † Departamento de Química Inorgánica, Instituto de Síntesis Química y Catálisis Homogénea-ISQCH, Universidad de Zaragoza-C.S.I.C., 50009 Zaragoza, Spain; ‡ Departamento de Farmacología y Fisiología, Medicina Legal y Forense, Unidad de Fisiología, Facultad de Veterinaria, Ciber de Fisiopatología de la Obesidad y Nutrición (CIBERobn), Instituto Agroalimentario de Aragón (IA2), 50013 Zaragoza, Spain; § Instituto de Investigación Sanitaria de Aragón (IIS Aragón), 50009 Zaragoza, Spain

## Abstract

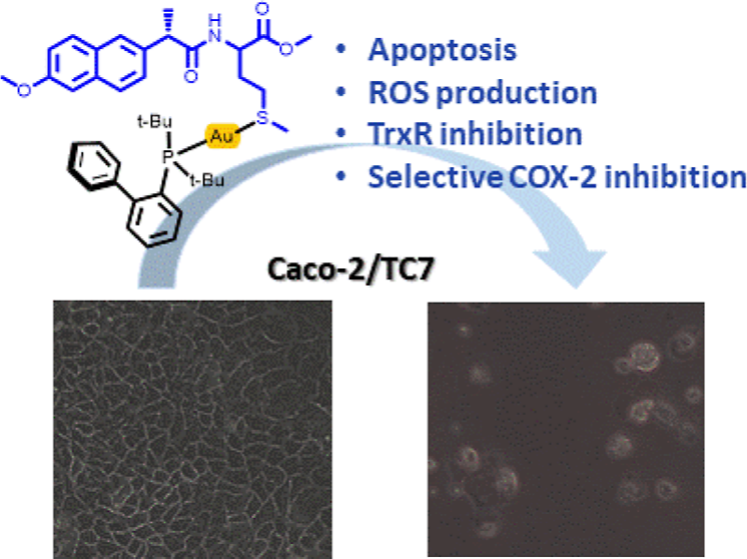

Targeting inflammation and the molecules involved in the inflammatory process could be an effective cancer prevention and therapy strategy. Therefore, the use of anti-inflammatory strategies, such as NSAIDs and metal-based drugs, has become a promising approach for preventing and treating cancer by targeting multiple pathways involved in tumor progression. The present work describes new phosphane gold(I) complexes derived from nonsteroidal anti-inflammatory drugs as multitarget drugs against colon cancer. The antiproliferative effect of the most active complexes, [Au(L3)(JohnPhos)] (**3b**), [Au(L4)(CyJohnPhos)] (**4a**) and [Au(L4)(JohnPhos)] (**4b**) against colon cancer cells (Caco2-/TC7) seems to be mediated by the inhibition of the enzyme cyclooxygenase-1/2, modulation of reactive oxygen species levels by targeting thioredoxin reductase (TrxR) activity, and induction of apoptosis in cancer cells. Additionally, the three complexes exhibit high selectivity index values toward noncancerous cells. The research highlights the importance of maintaining cellular redox balance and the role of TrxR in cancer cell survival.

## Abstract 2

Synthesis of new phosphane gold(I) complexes derived from NSAIDs as multitarget drugs against colon cancer, which show selective inhibition of COX-1/2 and TrxR, ROS production and apoptosis induction.

## Introduction

Colon cancer, also known as colorectal cancer, represents one of the most common cancers diagnosed in older adults being one of the leading causes of cancer deaths worldwide.^1^ While surgical resection and chemotherapy remain the main curative options for colon cancer, adjuvant therapy continues to play an important role in preventing disease recurrence and metastasis. Several factors increase the risk of developing colon cancer, including an aging population and dietary habits of high-income countries, family history of colon cancer or polyps, personal history of inflammatory bowel disease (such as Crohn’s disease or ulcerative colitis), and certain genetic syndromes.^2−4^ Chronic intestinal inflammation has been associated with the development of colon cancer. Recent studies indicate that in genetically predisposed individuals, the innate immune system could promote colon tumor development in chronic inflammation, particularly in response to specific native microorganisms or cellular debris.^5^ Inflammation is also likely to be involved in other forms of colon cancer, both sporadic and hereditary.^6^ Additionally, chronic inflammation stimulates cell proliferation, angiogenesis, and metastasis, while simultaneously reducing the immune system response and the effectiveness of chemotherapeutic agents.^7,8^ As a result, targeting inflammation and the molecules involved in the inflammatory process could represent a good strategy for cancer prevention and therapy.^9^

Many anti-inflammatory compounds, including NSAIDs (nonsteroidal anti-inflammatory drugs), have been reported to exhibit anticancer activities. They can disrupt the tumor microenvironment by diminishing cell migration while enhancing apoptosis and chemo-sensitivity.^9−13^ Specifically, for colorectal cancer, a great deal of epidemiological and preclinical studies endorse NSAIDs as a chemopreventive effect.^14^ Several clinical cancer trials to date have supported the chemopreventive and chemotherapeutic potential of NSAIDs alone or in combination with other drugs in colorectal cancer (NCT02052908, NCT01786200, NCT05411718, NCT01719926, NCT00473980, NCT00002796).^15^

The main anticancer mechanism attributed to NSAIDs is the inhibition of cyclooxygenases (COX),^16^ leading to a reduction in prostaglandin production, which plays a crucial role in several physiological processes, being particularly involved in the inflammatory response and the sensation of pain. This, in turn diminishes tumor cell proliferation and angiogenesis and promotes apoptosis. The two main isoforms COX-1 and COX-2 have a shared function in the metabolic process of arachidonic acid (AA), wherein biologically active prostaglandin (PG) species are produced from arachidonate.^17^

While COX-1 is constitutively expressed in many tissues and maintains homeostasis of some physiological functions, like safeguarding the stomach lining, regulating blood flow to the kidneys, and promoting platelet aggregation for blood clotting, COX-2 is triggered by inflammation and is a well-known tumor promoter.^18^ In addition, overexpression of the COX-2 gene occurs in many types of cancer, including colon cancer.^19−21^ Hence, the expression of COX-2 could indicate cancer development, making it a potential marker for the disease.^22^ Accordingly, COX-2 selective inhibition could be considered a promising strategy to fight colon cancer.^23,24^

Metal ions are essential participants in various biological processes, and the area of expertise dedicated to employing inorganic chemistry to treat or diagnose diseases, known as Medicinal Inorganic Chemistry, is on the rise.^25^ After the fortuitous discovery of the anticancer properties of cisplatin (*cis*-[PtCl_2_(NH_3_)_2_]) that demonstrated significant chemotherapeutic capabilities, a significant body of research on metal-based drugs has emerged. Cisplatin is commonly used in the treatment of various types of cancer, being particularly effective against testicular, ovarian, bladder and lung cancers, among others. Cisplatin interferes with the DNA inside cancer cells, preventing them from dividing and growing, ultimately leading to cancer cells’ death.^26^ However, the use of cisplatin and platinum-based anticancer drugs in chemotherapy is associated with several side effects, including nausea, vomiting, kidney damage and hearing loss, besides drug-resistance phenomena.^27,28^ To overcome these limitations, novel metallodrugs for cancer treatment have been designed, using nonplatinum metals like ruthenium or gold.

Research has shown that NSAID ligand scaffolds strongly tend to form complexes with metal ions. Many examples of metallic compounds with NSAID molecules have been reviewed in the literature.^29−32^ In most cases, the metal complexes of NSAID ligands are more active as drugs than the corresponding free NSAIDs, due to the synergistic effect of the metal ion together with the bioactive NSAID pharmacophore, resulting in a significantly enhanced biological response compared to the parent NSAIDs. Divalent cations such as Cu(II), Co(II), Mn(II), Ni(II) and Zn(II), besides Pt(IV),^33−37^ have the highest number of examples of NSAID-based complexes where the ligand binds through oxygen and/or nitrogen atoms. NSAIDs-based metallic complexes with heavier metals, such as ruthenium,^38,39^ silver,^40,41^ and gold^42−44^ are less commonly found. The mechanism of action of such NSAIDs-metal complexes varies upon complexation to the different metals. However, enhanced cytotoxicity is observed, attributed to multiple pathways in addition to COX-1 and COX-2 inhibition activity.

Gold complexes, particularly gold(I) complexes, have undergone extensive research due to their potential use in treating cancer. Auranofin [2,3,4,6-tetra-6-acetyl-1-thio-β-d-glucopyranoside-*S*-triphenylphosphane gold(I)] is one of the most well-known gold complexes utilized in this field and it has been studied for its ability to treat various types of cancer.^45−51^ One of the advantages of gold complexes in cancer therapy is their ability to target cancer cells while sparing healthy cells selectively. This selectivity is partly due to differences in redox regulation between cancerous and normal cells. Gold complexes and auranofin effectively generate reactive oxygen species (ROS) and inhibit thioredoxin reductase (TrxR),^45,52^ an essential enzyme that plays a crucial role in maintaining cellular redox balance and protecting cells from oxidative stress.^53^ Overexpression of TrxR has been observed in different types of cancer cells, including colon cancer.^54,55^ Consequently, this biological activity of gold complexes makes them a potential candidate for cancer therapy.

Besides, chemical modifications of the chemical structures of NSAIDs enable the investigation and development of more potent agents, that may introduce novel mechanisms of action, aiming to reduce or eliminate the side effects linked to COX inhibition, such as gastric bleeding and cardiovascular risks.^11^ With this idea, we have modified the structure of the NSAID molecule by attaching the ester of the essential amino acid l-methionine, which can serve as a directing group and facilitate its coordination to a gold center through the thioether moiety. Here we describe the synthesis of new NSAID ligands, the corresponding phosphane gold(I) derivatives and their evaluation against colon cancer cells, studies of their possible mechanism through the inhibition of enzymes thioredoxin reductase (TrxR), cyclooxygenase (COX, isoforms 1 and 2), as well as redox disturbances consequence of ROS generation and the type of cell death.

## Results and Discussion

### Synthesis and Characterization of Gold Complexes

**Scheme 1:**
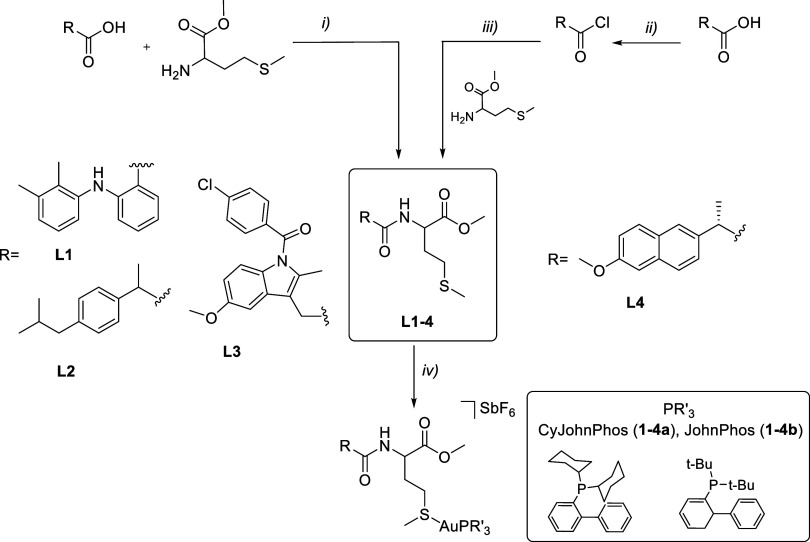
Synthesis of NSAIDs Derivatives and Their Gold Complexes

The synthesis of the new ligands (**L1–4**, Scheme 1) based on the modification of the skeleton of different NSAIDs (mefenamic acid (2-[(2,3-dimethylphenyl)amino]benzoic acid), ibuprofen (2-(4-isobutylphenyl)propionic acid) and indomethacin (2-[1-(4-chlorobenzoyl)-5-methoxy-2-methylindol-3-yl]acetic acid)) was performed via an esterification reaction between the NSAID and the α-amino acid l-methionine methyl ester in the presence of *N*-[3-(dimethylamino)propyl]-*N*′-ethylcarbodiimide hydrochloride (EDCI), a coupling agent, and NEt_3_ as a basic catalyst. In the case of naproxen, we started from (*S*)-6-methoxy-α-methyl-2-naphthaleneacetic acid, which under the reaction conditions, led to a mixture of diastereomers, resulting from a racemization process. Consequently, we devised an alternative method based on the in situ synthesis of the corresponding acetyl chloride, which was then reacted with l-methionine ester in the presence of diisopropylethylamine (DIPEA) [Scheme 1,(ii),(iii)]. Ligands **L1–4** were isolated by chromatographic purification in moderate or high yields (from 55 to 90%). The formation of the new ligands was evidenced by the disappearance of the signal characteristic of the acid group around 12 ppm and the appearance of the thioether methyl signal around 2 ppm from l-methionine in the ^1^H NMR spectra. Moreover, their vibration around 3300 cm^–1^ has disappeared in the IR spectra of the new molecules, in addition to the ν(CO) vibration due to the methionine moiety around 1740 cm^–1^.

Treatment of the new NSAIDs **L1–4** with [Au(NCMe)(PR’_3_)]SbF_6_ (PR’_3_ = CyJohnPhos (dicyclohexyl-(2-phenylphenyl)phosphane) and JohnPhos (di*tert*-butyl-(2-phenylphenyl)phosphane) in dichloromethane afforded the corresponding phosphane gold(I) derivatives **1–4a** with CyJohnPhos and **1–4b** with JohnPhos (Scheme 1) as air-stable solids in moderate yields. All complexes were characterized by ^1^H, ^31^P{^1^H}, and ^13^C NMR spectroscopy, IR spectroscopy, mass spectrometry, and elemental analysis.

Compounds with the CyJohnPhos phosphane show a singlet in the ^31^P{^1^H} NMR spectra at around 41 ppm. In comparison, those containing the JohnPhos unit appear at 63 ppm, confirming their coordination to the metal center. The ^1^H NMR spectra of complexes **1–4(a–b)** display more complicated pattern compared to the free ligands, because of the CyJohnPhos and JohnPhos phosphane presence in the aromatic region.

### Stability, Reactivity, and Protein Interactions of Phosphane Gold(I) Complexes in Physiological Conditions or Stability and Reactivity of Phosphane Gold(I) Complexes in Physiological Conditions

The stability of the new complexes was analyzed by UV–vis absorption spectroscopy in phosphate-buffered saline (PBS) solution (pH = 7.4). Solutions at a concentration of 5 × 10^–5^ M were prepared by diluting DMSO stock solutions of the complexes in PBS buffered at pH 7.4. The resulting solutions were monitored over 24 h at 37 °C (Figure S45). The spectra of the complexes exhibited an absorption band with low intensity at around 260 nm, which could be assigned as π → π* intraligand transitions. The UV–visible spectra demonstrate that the bands remained unchanged in shape over time, with nonobservable red or blue shifts in their maxima. However, a decrease in intensity was noted in the specific cases of complexes **2a** and **2b**, the less soluble compounds, being attributed to a reduction in the solubility of the complexes under 37 °C after a partial solvent evaporation. Over time, a slight turbidity was observed in the test cuvettes of such complexes, indicating this phenomenon. Importantly, no new absorbance bands were detected, ruling out dissociation processes, the formation of bisphosphane gold complexes, or other rearrangements in PBS. Furthermore, the absence of an absorbance around 500 nm over 24 h, characteristic of gold nanoparticle formation, further supports the considerable stability of the compounds under physiological conditions.

Gold can coordinate through ligand exchange reactions with cysteine and selenocysteine residues in the active sites of enzymes, leading to their inhibition.^52^ To assess the reactivity of the complexes with nucleophilic reagents, the complexes were exposed to an equimolar amount of *N*-acetyl-l-cysteine [N-acetyl cysteine (NAC)] in DMSO-*d*_6_ (containing 20% D_2_O) over 72 h and analyzed by ^1^H NMR spectroscopy (Figures S46–S55). The results showed that most of the complexes remained without changes in their spectra, in addition to new signal peaks generated by the auto-oxidation of NAC after 24 h (1.87, 3.09, and 4.45 ppm). However, complexes [Au(L1)(JohnPhos)]SbF_6_ (**1b**) and [Au(L2)(JohnPhos)]SbF_6_ (**2b**) reacted with NAC, since new signals in the aromatic region can be observed after 24 h and new singlets appear and increase over time in their ^31^P{^1^H} NMR spectra, which agrees on the formation of new species in solution. Considering that no signals of free phosphane or phosphane oxide are observed in the ^31^P{^1^H} NMR spectra and that in the aromatic region of the ^1^H NMR spectra, there are signals consistent with the presence of the ligand derived from the free NSAID, an exchange reaction of this ligand with NAC is postulated.

In addition to the stability studies of the complexes with NAC, we tested the reactivity of the most active complex **4b** and the NAC-reactive complexes **1b** and **2b**, against the tripeptide glutathione reduced (GSH) that can be representative of some protein binding site. The compounds were mixed with equimolar amounts of GSH in DMSO-*d*_6_ and D_2_O and their behavior was monitored by ^1^H and ^31^P{^1^H} NMR experiments (Figures S56–S61). The results indicate that complex **4b** is more stable than complexes **1b** and **2b**. In all the cases, new signals corresponding to the self-oxidation process of glutathione were observed (3.53, 3.12, 2.15 ppm) in their ^1^H NMR spectra, while the formation of a new species was observed immediately or after 24 h for complexes **2b** and **1b**, respectively. This has been corroborated in the ^31^P{^1^H} NMR spectra by the appearance of a new signal compatible with the presence of a new species resulting from the reaction with GSH after the release of the NSAID-derived ligand.

Furthermore, albumin, particularly bovine serum albumin (BSA), was selected as a model protein to study its reactivity toward the same [Au(L1)(JohnPhos)]SbF_6_ (**1b**), [Au(L2)(JohnPhos)]SbF_6_ (**2b**) and [Au(L4)(JohnPhos)]SbF_6_ (**4b**).

**Figure 1:**
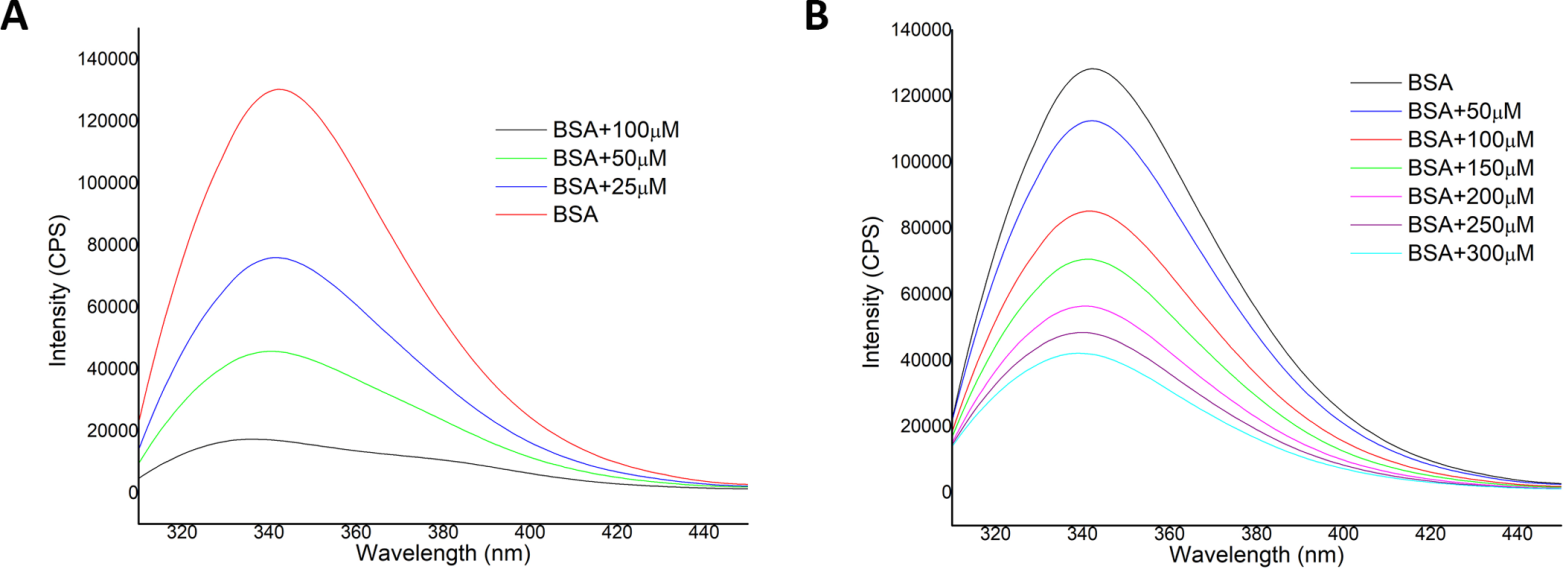
Fluorescence quenching spectra of BSA in the presence of various concentrations of complexes [Au(L1)(JohnPhos)]SbF_6_ (**1b**) (A) and [Au(L2)(JohnPhos)]SbF_6_ (**2b**) (B) (from 25 to 300 μM) at λ_excitation_ = 295 nm. [BSA] = 50 μM.

Fluorescence measurements provide information about the molecular environment of the chromophore molecule. BSA possesses two tryptophane residues (Trp134 and Trp212) with high emission intensity, and their fluorescence is sensitive to the environment, as changes in conformation or binding to a substrate can result in signal quenching. The fluorescence spectra of BSA in the presence of increasing amounts of complexes **1b** and **2b** were recorded in the range 310–450 nm upon excitation at 295 nm (Figure 1). Complex **4b** was excluded from the experiment since it emits within the same wavelength range as BSA. A concentration-dependent quenching of the fluorescence is observed, which is more pronounced in the case of complex **1b**, where saturation is reached at 100 μM of the complex. A slight shift in the maximum of the emission is observed in both cases, which is associated with changes in the polarity around the chromophore. These results suggest the presence of an interaction between both complexes and the tryptophan residue of BSA, which might be more intense in the case of complex **1a**.

To determine whether complex **4b** reacts with BSA, the possible reaction was monitored directly by visible absorption spectroscopy. A BSA solution was added to a buffered solution of complex **4b** in a 1:1 molar ratio, and the visible spectra were recorded along the time at room temperature (Figure S62). A decrease in intensity, along with changes in the shape of the visible bands, is observed after 24 h, which could be compatible with the formation of new species in solution as a consequence of the reaction between both chromophores.

Proteins are commonly considered primary targets for cytotoxic compounds due to their potential metal-binding sites. Such bonds occur mainly with the donor atoms of the side chains of amino acid residues, such as the thiolate and thioether sulfur atoms of l-cysteine and l-methionine, respectively. It is well established in the literature that gold complexes interact with such biomolecules thanks to the aurophilic nature of thiols and the high affinity of gold(I) to soft bases and nucleophiles.^56,57^ Studying the reactivity between gold derivatives and thiol-rich biomolecules can provide insight into the susceptibility of metallodrugs to side reactions when exposed to thiol-containing biomolecules, which could inactivate the gold complex.

NMR (^1^H and ^31^P{^1^H}) experiments carried out with *N*-acetyl-l-cysteine (NAC) in the presence of the new complexes suggest that no covalent bond has been formed with the coordinating amino acid, as no significant shift in the spectra of most of the derivatives has been observed. However, complexes [Au(L1)(JohnPhos)]SbF_6_ (**1b**), [Au(L2)(JohnPhos)]SbF_6_ (**2b**) with NSAIDs ibuprofen and mefenamic acid react with NAC. New signals appeared in the aromatic region, along with displacements of the NAC peaks in their ^1^H NMR, and new signals in their ^31^P{^1^H} NMR, consistent with a reaction with the biomolecule through NSAID dissociation. Similar results were obtained when the tripeptide glutathione (GSH) was used instead of NAC under the same experimental conditions with complexes **1b** and **2b**.

In the bloodstream, metallodrugs interact with the primary plasma binding protein, albumin, to be delivered to the specific bio target. For example, auranofin displays two types of interaction: the gold coordination to cys-34 and noncovalent interaction with hydrophobic pockets.^58,59^ Complexes **1b** and **2b** can quench the albumin’s intrinsic fluorescence emission, indicating alterations in the microenvironment surrounding the fluorescent tryptophan residues. This fact suggests the interaction of both complexes with subdomains IIA or IB, which contains the fluorescent amino acid residue. [Au(L1)(JohnPhos)]SbF_6_ (**1b**) seems to be more reactive or present greater affinity toward albumin than [Au(L2)(JohnPhos)]SbF_6_ (**2b**). Under the same conditions, complex **1b** led to luminescence saturation at 100 μM of the complex, while complex **2b** could only quench ca. 30% of albumin fluorescence. A higher degree of interaction or binding to BSA could decrease the free drug concentration, thereby affecting its biodistribution.

### Biological Studies

#### Analysis of the Antiproliferative Effect

The cytotoxicity of the new ligands **L1–4** and the corresponding phosphane gold(I) complexes **1–4a** with CyJohnPhos and **1–4b** with JohnPhos was assessed against human colon cancer cells, Caco-2/TC7, using the 2-(4,5-dimethyl-1,3-thiazol-2-yl)-3,5-diphenyl-2,3-dihydro-1H-tetrazol-4-ium bromide (MTT) assay (Table 1). The antiproliferative effect of the reference drug auranofin was included as positive control. The four new ligands exhibit low cytotoxic activity with IC_50_ values above 50 μM. However, the coordination of the gold-phosphane unit to these ligands derived from NSAIDs leads to much more cytotoxic complexes, as can be observed in Table 1 with IC_50_ values in the range of that found in auranofin or even lower values for complexes [Au(L1)(CyJohnPhos)] (**1a**), [Au(L4)(CyJohnPhos)] (**4a**), [Au(L3)(JohnPhos)] (**3b**) and [Au(L4)(JohnPhos)] (**4b**) (in the range 0.24–0.98 μM). Notably, the ligand derived from naproxen has the highest cytotoxicity values. This fact has already been observed previously in related gold complexes with an alkyne ligand derived from naproxen molecule,^43^ which makes naproxen an excellent pharmacophore when coordinated to gold(I).

**Table 1:** Distribution Coefficients and IC_50_ (μM)^b^ Values of the Complexes on Caco-2/TC7 Cancer Cells Compared with Auranofin^a^

compound	log *D*_7.4_	Caco-2/TC7	fibroblasts	SI
[Au(L1)(CyJohnPhos)]SbF_6_ (**1a**)	0.72	1.40 ± 0.55		
[Au(L2)(CyJohnPhos)]SbF_6_ (**2a**)	0.79	0.91 ± 0.18		
[Au(L3)(CyJohnPhos)]SbF_6_ (**3a**)	1.65	1.56 ± 0.95		
[Au(L4)(CyJohnPhos)]SbF_6_ (**4a**)	1.18	0.98 ± 0.65	7.32 ± 2.82	7.47
[Au(L1)(JohnPhos)]SbF_6_ (**1b**)	2.10	1.59 ± 0.45		
[Au(L2)(JohnPhos)]SbF_6_ (**2b**)	1.59	1.46 ± 0.43		
[Au(L3)(JohnPhos)]SbF_6_ (**3b**)	1.29	0.66 ± 0.33	9.91 ± 1.87	15.01
[Au(L4)(JohnPhos)]SbF_6_ (**4b**)	1.91	0.24 ± 0.04	7.94 ± 1.43	33.10
[Au(MeCN)(CyJohnPhos)]SbF_6_		1.24 ± 0.67		
[Au(MeCN)(JohnPhos)]SbF_6_		0.90 ± 0.76		
Auranofin	–2.53	1.80 ± 0.1		

a IC_50_ (M) values on fibroblasts are also presented.

b Mean ± SE of at least three determinations by using MTT method. SI = IC_50_(Fibroblast)/IC_50_ (Caco-2).

Based on the results obtained in this section regarding the antiproliferative effect and selectivity on cancer cells, complexes [Au(L3)(JohnPhos)]SbF_6_**(3b**), [Au(L4)(CyJohnPhos)]SbF_6_ (**4a**) and [Au(L4)(JohnPhos)]SbF_6_ (**4b**) were selected as the most promising complexes and their mechanism of action on undifferentiated Caco-2/TC7 cells was further analyzed. Besides, IC_50_ values on fibroblasts after incubation with these most active complexes showed similar values, nevertheless, the most active complexes **3b** and **4b** display high selectivity index (SI) (15.01 and 33.10, respectively) which establishes a greater selectivity of these complexes against cancer cells.

#### Analysis of the Inhibition of Enzymes COX-1/2

Both naproxen and indomethacin inhibit the activity of the cyclooxygenase-1 (COX-1), which is constitutively expressed and involved in maintaining normal physiological functions, and cyclooxygenase-2 (COX-2) enzymes, which is induced during inflammation and is associated with pain and inflammation responses. Both drugs reduce the production of prostaglandins, which are mediators of inflammation, pain and fever. The therapeutic roles due to COX-1 and COX-2 inhibition are anti-inflammatory, analgesic and antipyretic effects. Nevertheless, the negligent inactivation of COX by these drugs may entail potential side effects, such as gastrointestinal issues and an increased risk of cardiovascular events.^60,61^ Actually, selective COX-2 inhibitors were developed to reduce these gastrointestinal side effects; however, few of them are noted to enhance cardiovascular incident.^62^

**Figure 2:**
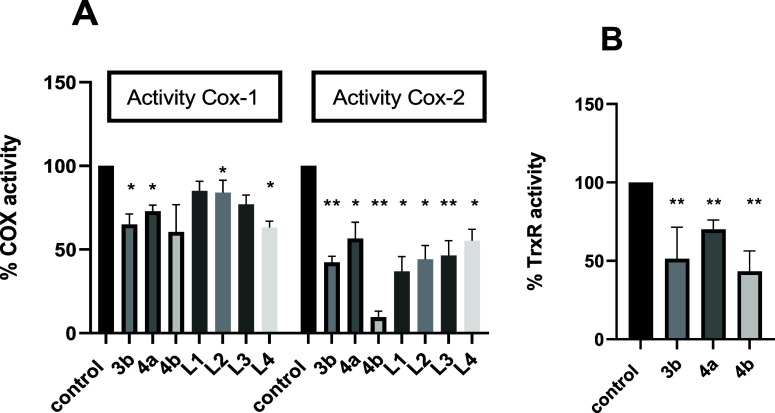
(A) Measurement of COX-1/COX-2 activity after complexes and free ligands **L1–4** incubation, and (B) TrxR activity after complexes incubation. Undifferentiated Caco-2/TC7 cells were incubated for 24 h with IC_50_ of the complexes [Au(L3)(JohnPhos)] (**3b**), [Au(L4)(CyJohnPhos)] (**4a**) and [Au(L4)(JohnPhos)] (**4b**). The results are expressed as mean values ± the standard error of the mean (SEM) (*n* ≥ 3 experiments). (*) p < 0.05; (**) p < 0.01 vs control.

Previous studies have shown that modifications in the structure of the naproxen molecule have led to effective inhibition of COX-2, with a high degree of selectivity compared to the COX-1 isoform.^63,64^ Thus, we have tested the inhibitory activity of the best active compounds [Au(L3)(JohnPhos)] (**3b**), [Au(L4)(CyJohnPhos)] (**4a**), and [Au(L4)(JohnPhos)] (**4b**), along with the free ligands **L1**, **L2**, **L3** and **L4**, against COX-1 and COX-2. For this purpose, a cyclooxygenase activity assay kit starting from Caco-2/TC7 cell lysates was used, aiming to develop a new class of inhibitors that present a balanced COX-1/COX-2 inhibition to control the severe side effects resulting from nonselective COX inhibition. Inhibition of COX enzymes is expressed as the percentage of inhibition relative to control COX-1 or COX-2 activity. Table 2 shows the percentage of inhibition of prostaglandin E2(PGE2) production via COX-1 and COX-2 and Figure 2 reports the corresponding activities of both enzymes. The free ligands **L1–L4** are not selective as they inhibit both isoforms with similar inhibition values, however, the three compounds were found to inhibit COX-2 with a higher selectivity than free ligands. Complex [Au(L4)(JohnPhos)] (**4b**) stands out for its remarkable selectivity for COX-2, achieving over 90% inhibition of COX-2 while inhibiting the COX-1 isoform by less than 40%. This fact underscores the importance of the coordination of the metal center to the NSAID derivative, since the presence of the gold center is essential for achieving selectivity toward the COX-2 isoform, similar to what is observed in other gold compounds for different biomolecules.^65,66^

**Table 2:** Percent Inhibition of COX-1, COX-2 and TrxR (Mean ± SEM)^a^

compound	COX-1	COX-2	TrxR
**L1**	15.2 ± 5.7	63.1 ± 8.9	n.d
**L2**	15.9 ± 7.3	55.8 ± 8.1	n.d
**L3**	22.9 ± 10.7	53.6 ± 14.3	n.d
**L4**	36.9 ± 7.6	44.7 ± 13.4	n.d
[Au(L3)(JohnPhos)] (**3b**)	35.0 ± 10.6	57.6 ± 6.2	48.6 ± 8.09
[Au(L4)(CyJohnPhos)] (**4a**)	27.1 ± 9.1	43.4 ± 23.8	29.9 ± 2.42
[Au(L4)(JohnPhos)] (**4b**)	39.4 ± 28.0	90.42 ± 6.1	56.64 ± 5.21

a Measurement of enzyme activity on undifferentiated Caco-2/TC7 cells upon 24 h incubation with IC_50_ of the complexes and 20 μM of free ligands.

Limited research data regarding the inhibitory effect of gold complexes on COX activity is available. Only inhibition of the COX-2 isoform has been described in previously published gold complexes with NSAID-type ligands.^42,43^ For the reference drug auranofin, inhibition of both isoforms has been documented, though higher concentrations are necessary to achieve this inhibition.^67−69^

#### Analysis of the Inhibition of Redox Enzyme TrxR

Thioredoxin reductase (TrxR) has been extensively studied in the context of cancer due to its crucial role in maintaining cellular redox homeostasis and regulating other activities within the cells. TrxR is often found to be upregulated in various types of cancer cells, including colon cancer.^70,71^ This increased expression is associated with enhanced antioxidant defense mechanisms, which promote cell survival and resistance to chemotherapy and radiation therapy. Consequently, inhibiting TrxR activity has emerged as a potential therapeutic approach for cancer treatment.^72−75^ By disrupting the redox balance and impairing antioxidant defense mechanisms in cancer cells, TrxR inhibitors can induce oxidative stress, inhibit cell proliferation, and promote apoptosis selectively in cancer cells. Most gold complexes can interact with thioredoxin reductase due to the affinity between the gold atom and the selenocysteine residue present on the active site of this enzyme.^76^

Accordingly, we determined the activity of TrxR in culture colon cancer cells treated with our NSAIDs-based complexes by using a thioredoxin reductase assay kit starting from Caco-2/TC7 cell lysates. As shown in Table 2 and Figure 2, our derivatives reduced TrxR activity by 30% to 57% compared to controls, with the naproxen complex **4a** showing the most significant decrease in enzyme activity.

### Determination of ROS Levels

As stated above, thioredoxin reductase plays a crucial role in maintaining cellular redox balance by reducing oxidized proteins and other molecules. By inhibiting TrxR activity, the reduction of oxidized proteins is impaired, leading to an accumulation of ROS. This fact occurs because TrxR is involved in the regeneration of reduced forms of antioxidants, such as glutathione, which help neutralize ROS. Inhibition of TrxR disrupts this antioxidant defense system and ROS accumulation is allowed.^53,77^

The increase in the ROS levels results in oxidative stress that was a consequence of an imbalance between its production and cellular antioxidant defense mechanisms. However, the mitochondria itself is sensitive to excessive ROS burst, which may induce its depolarization and release pro-apoptotic factors that stimulate one or more apoptosis. Such apoptosis activation serves as a protective mechanism to eliminate cells with severe oxidative damage. Inhibition of TrxR and the subsequent increase in ROS levels have been explored as a potential strategy for cancer therapy.^78,79^ Cancer cells are highly sensitive to oxidative stress due to their altered cellular redox balance, resulting from excessive growth rate, low metabolism, or p53 insufficiency. Therefore, inhibition of TrxR and increased levels of ROS can selectively target these cancer cells.

**Figure 3:**
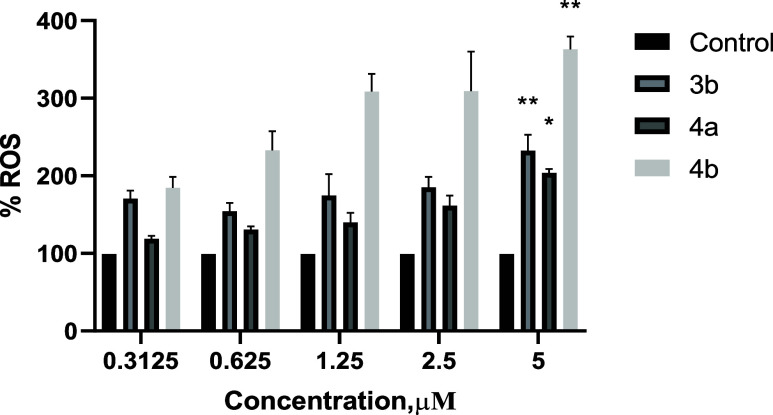
Measurement of ROS production on undifferentiated Caco-2/TC7 cells upon 24 h incubation with increasing concentrations of the complexes, [Au(L3)(JohnPhos)] (**3b**), [Au(L4)(CyJohnPhos)] (**4a**) and [Au(L4)(JohnPhos)] (**4b**). The results are expressed as mean values ± SEM (*n* ≥ 3 experiments). (*) *p* < 0.05; (**) *p* < 0.01 vs control.

Considering that our complexes can inhibit TrxR activity, we have studied their potential impact on ROS production. Given that H_2_O_2_ scavenging is one of the primary antioxidant functions of TrxR, we conducted a fluorometric assay using 6′-acetyloxy-2′,7′-dichloro-3-oxospiro[2-benzofuran-1,9′-xanthene]-3′-yl) acetate (2,7-dichlorodihydrofluorescein diacetate, DCFH-DA), that is converted by H_2_O_2_ to 2′,7′-dichloro-3′,6′-dihydroxyspiro[2-benzofuran-3,9′-xanthene]-1-one (2,7-dichlorofluorescein, DCF) within cells. This assay allowed us to quantify intracellular H_2_O_2_ levels in Caco-2/TC7 cells after treating them with increased concentrations of complexes [Au(L3)(JohnPhos)] (**3b**), [Au(L4)(CyJohnPhos)] (**4a**) and [Au(L4)(JohnPhos)] (**4b**). Treatment with the complexes resulted in an increased ROS generation upon 24 h incubation. As shown in Figure 3, these complexes produced a concentration-dependent pro-oxidant effect, which agrees with the inhibitory effect found on the TrxR enzyme (Figure 2). Indeed, previous studies have demonstrated that many gold complexes not only inhibit thioredoxin reductase activity but also have the potential to increase levels of ROS in cancer cells.^80−85^

### Cell Death Studies

After evaluating the mechanism of action of the multitarget complexes [Au(L3)(JohnPhos)] (**3b**), [Au(L4)(CyJohnPhos)] (**4a**) and [Au(L4)(JohnPhos)] (**4b**), we proceeded to analyze the type of cell death induced by these compounds.

**Figure 4:**
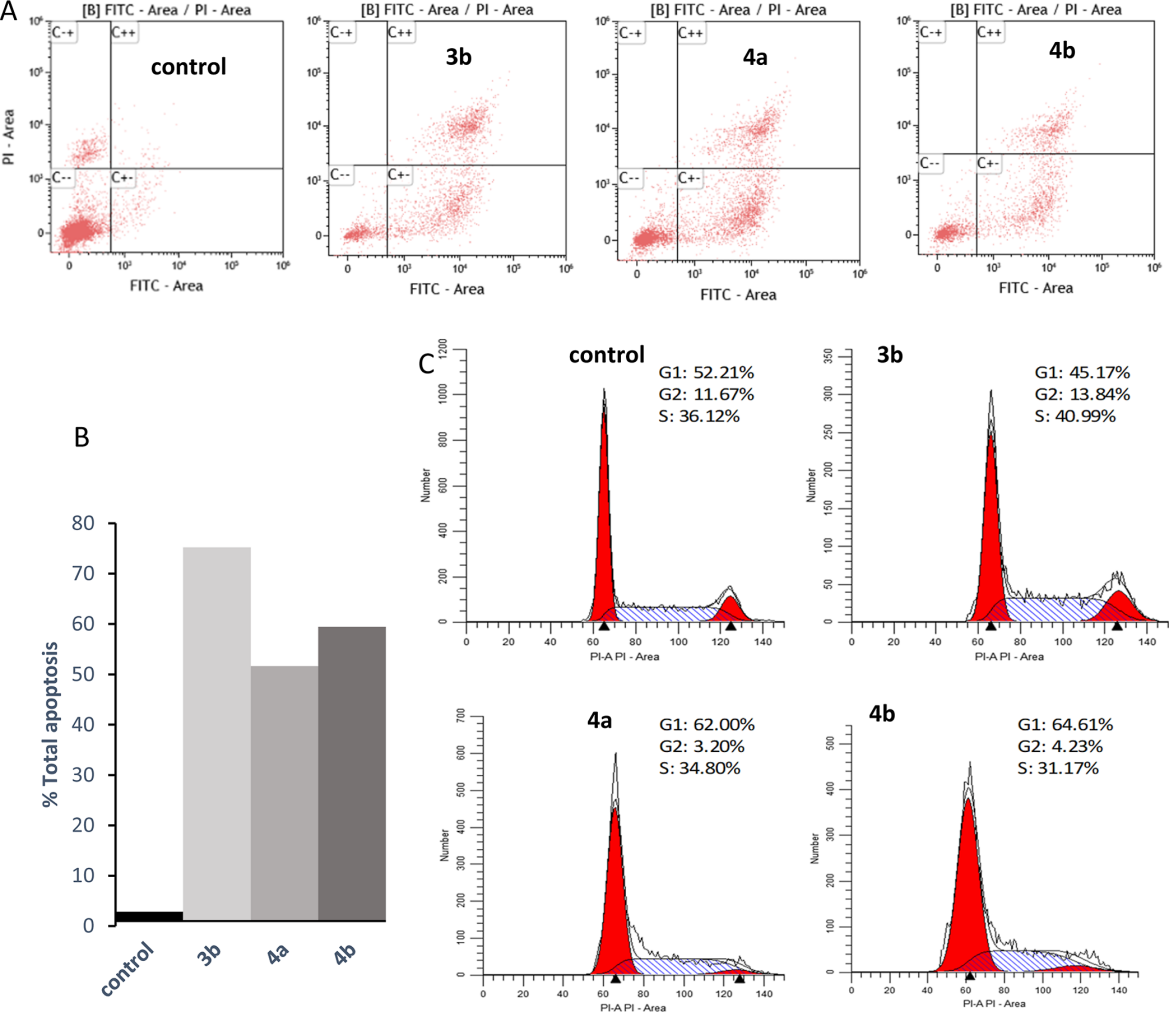
Analysis of the type of cell death induced on Caco-2/TC7 cells after 48 h incubation with [Au(L3)(JohnPhos)] (**3b**), [Au(L4)(CyJohnPhos)] (**4a**) and [Au(L4)(JohnPhos)] (**4b**) (4 × IC_50_ μM). (A) Fluorescence histograms of the distribution of cell populations in different stages: necrosis (C–+), living cells (C–−), late apoptosis (C++) and early apoptosis (C+–), in control and gold(I) complexes treated Caco-2/TC7 cells. (B) Apoptosis values, early + late, (%) of cells treated with gold(I) complexes, (C) cell cycle analysis. Percentages of each cell population are included.

Apoptosis is an innate process that enables tissue homeostasis by eliminating degenerated or misguided cells.^86^ In most cases, the dysregulation of apoptosis is commonly observed in cancer leading to uncontrolled cell growth and survival. Therefore, inducing apoptosis in cancer cells offers a promising area for developing more effective targeted therapies with reduced side effects. We have studied the ability of the three compounds to induce apoptosis by flow cytometry by using a combination of the apoptotic markers 3,8-diamino-5-{3-[diethyl(methyl)ammonio]propyl}-6-phenylphenanthridinium diiodide (propidium iodide, PI) and annexin V and compared to nontreated cells. As depicted in Figures 4A and S63, the three complexes show a considerable increase in the population of Caco-2/TC7 cells undergoing apoptosis (in early and late stages) compared to control after 48 h incubation. It is particularly noteworthy that total apoptosis events are increased a 26-up fold after treatment with complex **3b**, with a significant increase in late apoptotic population (34.33) in comparison with negative control (0.58) (Figure 4B). Besides, no significant changes in the percentage of cells undergoing necrosis were noticed.

Given the inhibitory effect of our complexes on cell proliferation, we sought to investigate if these compounds could induce any disruptions in the normal progression of the cell cycle. In our experiments conducted on cancer Caco-2/TC7 cells treated with the three gold(I) derivatives for 48 h, we observed that complex [Au(L3)(JohnPhos)] (**3b**) with NSAID derived from indomethacin did not have any noticeable effect on the cell cycle. However, the naproxen-derived complexes **4a** and **4b** were found to induce arrest in the G1-phase leading to an increased percentage of cells in the G1-phase and a decreased percentage of cells in the S- and G2/M-phases (Figure 4C), suggesting a specific disruption in DNA replication.

Cell cycle arrest in the G1 phase is essential for cancer development and apoptosis. The G1 phase serves as a critical checkpoint where cells undergo a series of regulatory processes to ensure proper DNA replication and cell division.^87^ There is often a disruption in the normal cell cycle progression leading to an abbreviated cell cycle and circumvention from the G1 checkpoint in cancer cells. Cancer cells can continuously divide and proliferate, contributing to tumor growth. The induction of cell cycle arrest at the G1 phase is an interesting strategy for antitumor therapy since stops cell division and growth, offering an opportunity to restore normal cell cycle regulation preventing cancerous cells from uncontrolled proliferation. Cell cycle arrest might be indicative of DNA damage and is usually considered an apoptotic biomarker as well.^88,89^

It has been reported that COX-2 inhibitors are suggested to cause cell cycle arrest in G1 and S phases.^90,91^ In our case, the complexes act as COX-2 inhibitors, with [Au(L4)(JohnPhos)] (**4b**) being the most active, selective against COX-2 and with the highest G1-phase arrest.

## Conclusion

Despite significant advancements in cancer therapy, the ongoing quest for new and more effective treatments remains crucial. The polypharmacologic approach, which involves designing drugs that target multiple proteins and pathways implicated in cancer development, has emerged as a promising strategy. Such multitarget drugs often outperform single-target therapies by enhancing efficacy and reducing resistance. The design and synthesis of hybrid molecules with multitarget capabilities have recently attracted considerable scientific interest. These chimeric compounds can be finely tuned to maximize therapeutic benefits while minimizing adverse off-target effects.

This study highlights the potential of novel phosphane gold(I) complexes derived from nonsteroidal anti-inflammatory drugs (NSAIDs) as multitarget agents against colon cancer. The synthesized complexes, notably [Au(L3)(JohnPhos)] (**3b**), [Au(L4)(CyJohnPhos)] (**4a**), and [Au(L4)(JohnPhos)] (**4b**), demonstrated significant antiproliferative activity against colon cancer cells, with a marked preference for cancerous cells over noncancerous ones. The anticancer effects of these complexes are primarily due to their ability to inhibit cyclooxygenase enzymes (COX-1/2), modulate reactive oxygen species (ROS) levels by targeting thioredoxin reductase (TrxR), and induce apoptosis in cancer cells. It is worth mentioning that although the new ligands derived from nonsteroidal drugs inhibit both isoforms of the COX enzyme with minimal selectivity the complexes show selectivity for the inhibition of COX-2. This finding highlights the importance of coordinating the ligands to a metal center.

Of particular interest is the complex [Au(L4)(JohnPhos)] (**4b**), which exhibited exceptional selectivity toward COX-2, achieving over 90% inhibition, a substantial improvement over both auranofin, the gold precursor, and the free ligand. This selectivity is crucial considering that COX-2 is activated by inflammation, being a tumor promoter and it is overexpressed in colon cancer as well as in many other types of cancer. Consequently, its inhibition could minimize the side effects typically associated with nonselective COX inhibition. Moreover, the complexes’ inhibition of TrxR underscores the importance of disrupting redox homeostasis in cancer cells, further highlighting their potential as targeted cancer therapies.

The stability of these complexes under physiological conditions and their selective reactivity with nucleophilic agents like NAC and the tripeptide glutathione reduced (GSH) that can be representative of some protein binding sites, reinforce their viability as therapeutic candidates. Only complexes **1b** and **2b** react with both biomolecules under the tested conditions, which suggests that the gold compounds undergo various exchange reactions in physiological conditions, particularly with thiol- or selenol-containing enzymes present in the tumor microenvironment, through the release of ligands **L1–4**. This research significantly contributes to medicinal inorganic chemistry by introducing a new class of gold-based compounds with dual mechanisms of action, targeting both inflammation and oxidative stress pathways in colon cancer treatment.

Future research should focus on their anti-inflammatory targeting capabilities, the in vivo evaluations of these complexes, and the exploration of their potential in combination with existing chemotherapeutic agents to enhance efficacy and reduce side effects. The findings of this study open the door for the development of more potent and selective gold(I) complexes as therapeutic agents in the fight against colon cancer.

## Experimental Section

### General

All chemicals and spectroscopic-grade solvents were commercially acquired and utilized without additional purification. Solvent drying and usage followed standard procedures. [AuCl(tht)], [AuCl(JohnPhos)], [AuCl(CyJohnPhos)], [Au(NCMe)(JohnPhos)]SbF_6_, [Au(NCMe)(CyJohnPhos)]SbF_6_^92^ were prepared according to published procedures and their experimental data agrees with that reported somewhere else.

All other reagents were commercially available and used without further purification, ^1^H, ^13^C{^1^H}, and ^31^P{^1^H} were recorded on a Bruker Avance 400 or a Bruker ARX 300 spectrometers, Chemical shifts (δ ppm) were reported relative to the solvent peaks in the ^1^H, ^13^C spectra or external 85% H_3_PO_4_ or CFCl_3_ in ^31^P or ^19^F spectra. IR spectra were recorded in the range 4000–200 cm^–1^ on a PerkinElmer Spectrum 100 spectrophotometer on solid samples using an ATR accessory. C, H, and N analyses were carried out with a PerkinElmer 2400 Series 2 microanalyser. Mass spectra were recorded on a BRUKER ESQUIRE 3000 PLUS, with the electrospray (ESI) or MALDI techniques.

### Synthesis of the Ligands

#### General Procedure for the Preparation of the Ligands (**L1–3**)

To a stirred solution of nonsteroidal anti-inflammatory drugs (1.3, 1 mmol) in DMF (5 mL) was added NEt_3_ (4 mmol), EDCI (1.5 mmol), 1-hydroxidbenzotriazole (1.5 mmol) at 0 °C. The reaction mixture was stirred for 30 min. l-methionine methyl ester (1 mmol) was added and the suspension was stirred for an additional 24 h under argon. The solvent was removed under reduced pressure and after chromatography on silica (Hexane/ethyl acetate 7/3) yielded pure compounds.
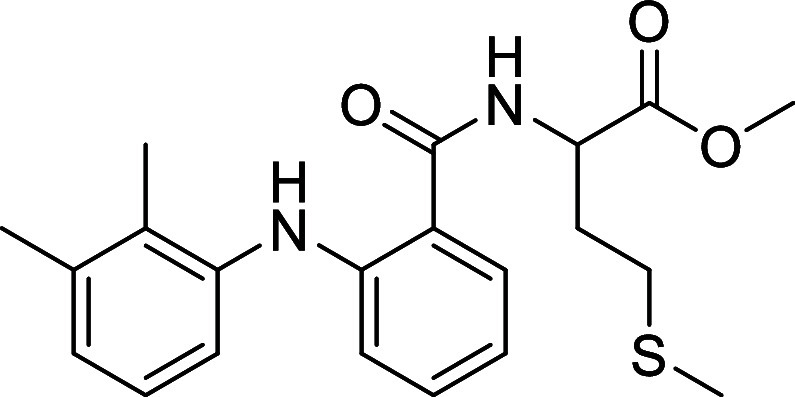


##### Methyl (2-((2,3-Dimethylphenyl)amino)benzoyl)methioninate (**L1**)

Yield 65.3%, pale-yellow solid, ^1^H NMR (400 MHz, CDCl_3_, 20 °C): δ 9.14 (s, 1H), 7.52 (dd, *J* = 7.9, 1.6, 1H), 7.22 (ddd, *J* = 8.6, 7.1. 1.6, 1H), 7.19–7.11 (m, 1H), 7.07 (t, *J* = 7.7, 1H), 6.99–6.94 (m, 1H), 6.93 (d, *J* = 7.7, 1H), 6.88 (dd, *J* = 8.5, 1.1, 1H), 6.71 (ddd, *J* = 8.1, 7.1, 1.1, 1H), 4.92 (td, *J* = 7.2, 5.1, 1H), ^13^C{^1^H} NMR (101 MHz, CDCl_3_, 20 °C): δ 172.62, 169.32, 147.60, 139.32, 138.08, 132.72, 131.28, 127.66, 125.92, 125.80, 121.49, 116.73, 115.71, 114.86, 52.67, 51.81, 31.64, 30.10, 20.66, 15.57, 13.95. IR ν_max_/cm^–1^: 3429, 3259 (NH); 2987, 2901(C_sp3_–H); 1732, 1640(CO); ESI-MS *m*/*z* (%): 409.1555 [M + Na]^+^. Anal. Calcd, (%) for C_21_H_26_N_2_O_3_S (86.16): C, 72.74; H, 9.57; N, 4.59; S, 5.25. Found: C, 72.64; H, 9.63; N, 4.60; S, 5.28.
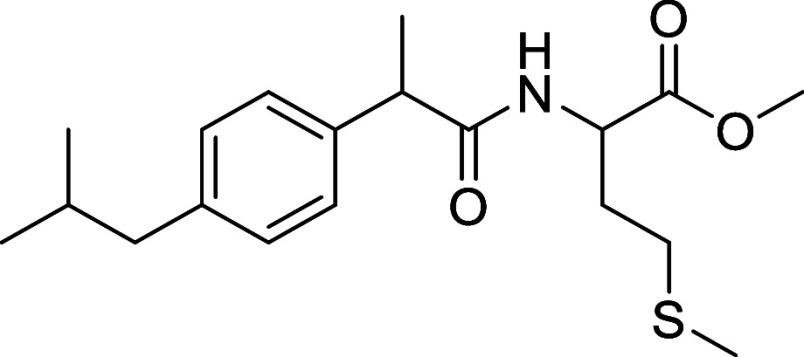


##### Methyl (2-(4-Isobutylphenyl)propanoyl)methioninate (**L2**)

Yield 89.2%; White Solid ^1^H NMR (400 MHz, DMSO-*d*_6_) δ: 8.35 (d, *J* = 7.6, 1H), 7.21 (d, *J* = 8.1, 2H), 7.08 (d, *J* = 8.1, 2H), 4.36 (ddd, *J* = 9.1, 7.5, 5.0, 1H), 3.65 (q, *J* = 7.0, 1H), 3.55 (s, 3H), 2.47 (m, 2H), 2.41 (d, *J* = 7.2, 2H), 2.02 (s, 3H),1.92 (m, 2H), 1.79 (h, *J* = 6.2 Hz, 1H), 1.30 (d, *J* = 7.1 Hz, 3H), 0.85 (d, *J* = 6.7 Hz, 6H) ppm. ^13^C{^1^H} NMR (101 MHz, CDCl_3_, 20 °C): δ:173.69, 172.19, 139.14, 139.09, 127.01, 51.73, 50.94, 44.24, 30.46, 29.55, 22.17, 22.15, 18.69, 14.56. IR ν_max_/cm^–1^: 3404 (NH); 2970, 2901 (C_sp3_–H); 1741; 1675 (CO), ESI-MS *m*/*z* (%):374.1758 [M + Na]^+^. Anal. Calcd (%) for C_19_H_29_NO_3_S (351.18): C, 64.92; H, 8.32; N, 3.98; O, 13.65; S, 9.12. Found: C. 64.83; H. 8.27; N. 3.97; S. 9.08.
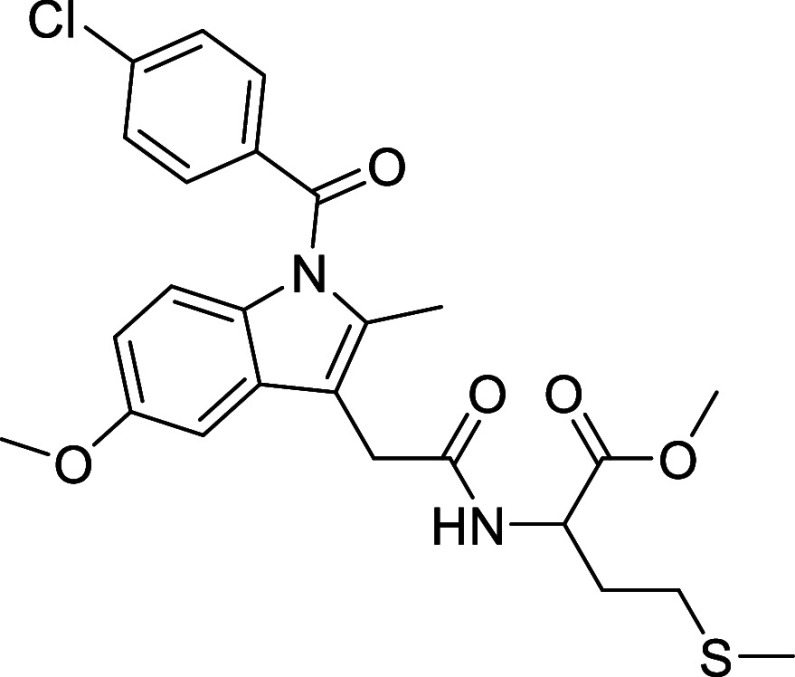


##### (2-(1-(4-Chlorobenzoyl)-5-methoxy-2-methyl-1*H*-indol-3-yl)acetyl)methioninate (**L3**)

Yield 59.6%; yellow solid ^1^H NMR (400 MHz, CDCl_3_, 20 °C): δ 7.67 (d, *J* = 8.8, 1H), 7.48 (d, *J* = 2.5, 1H), 6.95 (d, *J* = 9.1, 1H), 6.91 (d, *J* = 2.5, 1H), 6.71 (dd, *J* = 9.0, 2.5, 1H), 6.46 (d, *J* = 8.0, 1H), 4.71 (td, *J* = 7.5, 7.5, 4.9, 1H), 2.83 (s, 3H), 3.70 (s, 3H), 3.67(s, 2H), 2.36 (m, 5H), 2.08 (m, 1H), 1.90 (m, 4H) ppm. ^13^C{^1^H} NMR (101 MHz, CDCl_3_, 20 °C): δ 172.05, 169.92, 168.33, 156.30, 139.49, 136.32, 133.70, 131.25, 130.99, 130.25, 129.22, 115.17, 112.55, 112.42, 100.78, 55.75, 52.50, 51.71, 32.16, 30.77, 29.97, 15.25, 13.38. IR ν_max_/cm^–1^: 3317 (NH), 2969, 2927 (C_sp3_–H), 1743, 1665, 1645 (CO). ESI-MS *m*/*z* (%): 525.1221 [M + Na]^+^. Anal. Calcd (%) for C_25_H_27_ClN_2_O_5_S (502.13):C, 59.70; H, 5.41; Cl, 7.05; N, 5.57; O, 15.90; S, 6.37. Found C. 59.77; H. 5.44; N. 5.56; S.6.41.
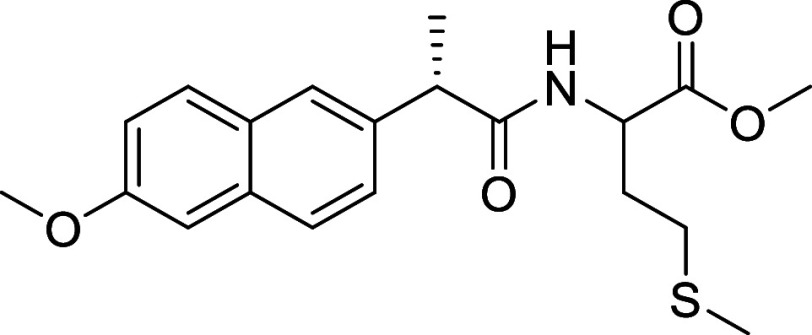


##### Synthesis of Methyl ((*S*)-2-(6-Methoxynaphthalen-2-yl)propanoyl)methioninate (**L4**)

Naproxen (0.5 mmol) was dissolved in dry CH_2_Cl_2_ (2 mL) with 3 drops of DMF at 0 °C. Oxalyl chloride (1.03 mmol) was added, and the reaction mixture was stirred for 3 h, under argon. All the volatiles were removed under pressure. We added CH_2_Cl_2_ (3 mL), followed by the dropwise addition of a DIPEA solution (2.07 mmol) in CH_2_Cl_2_ (2 mL) to the mixture of acyl chloride at 0 °C. Finally, we added the l-methionine methyl ester (0.75 mmol). The reaction was stirred for 4 h. The reaction mixture was washed with HCl (5 M, 20 mL) and the aqueous was extracted with CH_2_Cl_2_ (2 × 20 mL). The organic phase was dried over anhydrous magnesium sulfate. The product was purified by flash column chromatography (6 Hexane/4 ethyl acetate). A white solid was obtained as the final product (0.105 g, 55.8%). ^1^H NMR (400 MHz DMSO): δ 7.76–7.66 (m, 3H), 7.38 (dd, *J* = 8.5, 1.9, 1H), 7.22–7.09 (m, 2H), 6.13 (d, *J* = 7.9, 1H), 4.66 (td, *J* = 7.6, 5.1, 1H), 3.75 (q, *J* = 7.2, 1H), 3.65 (s, 3H), 2.40 (t, *J* = 7.4, 2H), 2.03 (s, 1H), 1.94–1.84 (m, 1H), 1.61 (d, *J* = 7.2, 3H) ppm, ^13^C{^1^H} NMR (101 MHz, CDCl_3_): δ 174.29, 172.22, 157.77, 135.84, 133.82, 129.27, 129.01, 127.55, 126.30, 126.26, 119.16, 105.67, 52.42, 51.69, 46.98, 31.31, 30.00, 18.41, 15.40 ppm, IR ν_max_/cm^–1^: 3333 (NH), 2977, 2914 (C_sp3_–H), 1733, 1651 (CO) ESI- MS *m*/*z* (%): 398.1396 [M + Na]^+^. Anal. Calcd (%) for C_20_H_25_NO_4_S (375.15): C, 63.98; H, 6.71; N, 3.73; S, 8.54. Found: C. 64.06; H. 6.81; N, 3.77; S. 8.57.

#### Synthesis of the Complexes, Synthesis of Gold(I) Complexes **1a–4a** and **1b–4b** (a = CyJohnPhos, b = JohnPhos)

To a solution of the desired methioninate (0.1 mmol) in CH_2_Cl_2_ (5 mL) was treated with the corresponding gold(I) precursor ([Au(NCMe)(CyJohnPhos)]SbF_6_ (**a**) or [Au(NCMe)(JohnPhos)]SbF_6_ (**b**), 0.1 mmol). After 6 h of reaction, the solvent was evaporated to a minimum volume under vacuum and a precipitate was obtained by the addition of cold hexane. The solid was filtered, washed with hexane and dried.

##### [Au(L1)(CyJohnPhos)] (**1a**)

60% yield, pale-yellow solid, ^1^H NMR (400 MHz, CDCl_3_): δ 9.22 (s, 1H), 7.62 (d, *J* = 7.9 Hz, 1H), 7.56 (m, 2H), 7.48 (m, 3H), 7.31 (m, 1H), 7.23 (m, 4H), 7.12 (d, *J* = 8.5 Hz, 1H), 7.05 (t, *J* = 7.7 Hz, 1H), 6.95 (d, *J* = 7.4 Hz, 1H), 6.86 (dd, *J* = 8.5, 1.1 Hz, 1H), 6.77 (t, *J* = 7.5, 7.5 Hz, 1H), 4.81 (q, *J* = 6.8, 6.8, 6.7 Hz, 1H), 3.80 (s, 3H), 3.01 (m, 2H), 2.43 (s, 3H), 2.31 (m, 5H), 2.16 (s, 3H), 2.02 (m, 2H), 1.80 (m, 4H), 1.66 (m, 4H), 1.57 (s, 3H), 1.20 (m, 12H) ppm. ^31^P{^1^H} NMR (162 MHz, CDCl_3_): δ 41.74. ppm. ^13^C{^1^H} NMR (101 MHz, CDCl_3_): δ 171.56, 169.70, 148.98, 148.86, 147.60, 139.43, 138.05, 132.92, 132.55, 131.38, 131.16, 129.90, 128.99, 128.08, 125.85, 125.81, 121.45, 117.22, 115.16, 114.73, 55.90, 52.92, 51.49, 36.10, 35.77, 31.70, 31.09, 29.37, 26.52, 26.39, 26.23, 25.61, 20.64, 13.93. ppm, IR ν_max_/cm^–1^: 3417 (NH), 2987,2971,2900 (C_sp3_–H), 1740, 1639, (CO) 705, 655(Sb–F), MALDI MS *m*/*z* (%): 932.839 (100) [M–SbF_6_]^+^. Anal. Calcd (%) for C_45_H_57_AuF_6_N_2_O_3_PSSb (1168.24): C, 46.21; H, 4.91; N, 2.39; S, 2.74. Found: C, 46.14; H, 4.94; N, 2.48; S, 2.79.

##### [Au(L2)(CyJohnPhos)] (**2a**)

55% yield, yellow solid, ^1^H NMR (400 MHz, DMSO-*d*_6_): δ 8.40 (d, *J* = 7.8 Hz, 1H), 7.92 (m, 1H), 7.65 (m, 2H), 7.51 (s, 3H), 7.34 (q, *J* = 4.8, 4.5, 4.5 Hz, 1H), 7.26 (d, *J* = 6.5 Hz, 2H), 7.20 (d, *J* = 8.1 Hz, 2H), 7.08 (d, *J* = 8.0 Hz, 2H), 4.38 (td, *J* = 8.5, 8.5, 4.9 Hz, 1H), 3.65 (q, *J* = 6.9 Hz, 1H), 3.56 (s, 3H), 2.82 (m, 2H), 2.59 (d, *J* = 11.2 Hz, 2H), 2.40 (d, *J* = 7.1 Hz, 2H), 2.36 (s, 3H), 2.00 (m, 5H), 1.79 (m, 3H), 1.66 (m, 6H), 1.29 (m, 9H), 1.14 (t, *J* = 11.9, Hz, 4H), 0.85 (d, *J* = 6.6 Hz, 7H) ppm. ^31^P{^1^H} NMR (162 MHz, CDCl_3_): δ 41.87 ppm. 13C{1H} NMR (101 MHz, CDCl_3_): δ 173.70, 171.55, 148.30, 148.18, 141.67, 141.60, 139.24, 138.97, 133.17, 131.99, 131.91, 131.38, 129.56, 128.74, 128.70, 128.26, 127.91, 126.97, 51.90, 50.67, 44.30, 44.19, 35.27, 34.94, 32.87, 30.68, 29.60, 29.10, 25.86, 25.74, 25.70, 25.23, 22.14, 22.12, 18.67, 17.95 ppm. IR ν_max_/cm^–1^: 3404 (NH), 2990, 2970, 2901 (C_sp3_–H), 1741, 1675, (CO) 704, 654(Sb–F) MALDI MS *m*/*z* (%): 897.866 (100) [M–SbF_6_]^+^. Anal. Calcd, (%) for C_43_H_60_AuF_6_NO_3_PSSb (1133.26): C, 46.21; H, 4.91; N, 2.39; S. 2.74. Found: C, 45.88; H, 5.47; N, 1.28; S, 2.88.

##### [Au(L3)(CyJohnPhos)] (**3a**)

41% yield, yellow solid, ^1^H NMR (400 MHz, CDCl_3_): δ 7.67 (d, *J* = 8.5 Hz, 2H), 7.57 (d, *J* = 3.0 Hz, 3H), 7.47 (m, 4H), 7.32 (m, 1H), 7.22 (m, 2H), 7.02 (d, *J* = 2.5 Hz, 1H), 6.95 (d, *J* = 9.0 Hz, 1H), 6.80 (d, *J* = 8.2 Hz, 1H), 6.66 (dd, *J* = 9.0, 2.5 Hz, 1H), 4.59 (td, *J* = 7.2, 7.1 Hz, 1H), 3.84 (s, 3H), 3.68 (s, 6H), 2.99 (m, 2H), 2.42 (d, *J* = 1.5 Hz, 3H), 2.37 (s, 3H), 2.21 (m, 2H), 2.02 (m, 2H), 1.78 (m, 6H), 1.58 (s, 4H), 1.29 (ddd, *J* = 54.7, 35.2, 12.9 Hz, 12H) ppm. ^31^P{^1^H} NMR (162 MHz, CDCl_3_): δ 41.76 ppm. ^13^C{^1^H} NMR (101 MHz. CDCl_3_): δ 71.48, 171.29, 168.86, 156.58, 149.28, 142.56, 139.54, 136.69, 134.52, 132.98, 131.90, 131.66, 131.41, 131.15, 130.36, 129.55, 129.43, 128.60, 123.71, 115.50, 113.60, 112.39, 101.63, 56.28, 53.18, 51.70, 36.53, 36.21, 35.48, 32.25, 31.95, 31.53, 29.81, 26.97, 26.84, 26.69, 26.07, 20.45, 13.90 ppm, IR ν_max_/cm^–1^: 3417 (NH), 2987, 2971, 2900 (C_sp3_–H), 1740, 1639, (CO) 705, 655(Sb–F), MALDI MS *m*/*z* (%): 1048.729 (98.93) [M–SbF_6_]^+^. Anal. Calcd (%) for C_49_H_58_AuClF_6_N_2_O_5_PSSb (1284.21): C, 45.76; H, 4.55; N, 2.18; S, 2.49. Found: C, 45.85; H, 4.67; N, 2.21; S, 2.52.

##### [Au(L4)(CyJohnPhos)] (**4a**)

47% Yield, White Solid, ^1^H NMR (400 MHz, DMSO-*d*_6_): δ 8.46 (d, *J* = 7.7 Hz, 1H), 7.91 (dt, *J* = 9.2, 4.7, 4.7 Hz, 1H), 7.75 (dd, *J* = 8.7, 6.8 Hz, 2H), 7.70 (d, *J* = 1.7 Hz, 1H), 7.64 (m, 2H), 7.49 (m, 3H), 7.42 (dd, *J* = 8.6, 1.8 Hz, 1H), 7.32 (q, *J* = 4.8, 4.6, 4.6 Hz, 1H), 7.26 (m, 3H), 7.13 (dd, *J* = 9.0, 2.6 Hz, 1H), 4.40 (td, *J* = 8.5, 8.5, 5.0 Hz, 1H), 3.85 (s, 3H), 3.80 (q, *J* = 7.0, 7.0 Hz, 1H), 3.53 (s, 3H), 2.81 (m, 2H), 2.50 (m, 2H), 2.33 (s, 3H), 1.97 (m, 3H), 1.68 (m, 8H), 1.40 (d, *J* = 7.0 Hz, 2H), 1.29 (m, 7H), 1.13 (m, 4H).ppm, ^31^P{^1^H} NMR (162 MHz, DMSO) δ: 41.42 ppm. ^13^C{^1^H} NMR (101 MHz, DMSO-*d*_6_): δ 173.71, 171.62, 157.03, 148.33, 141.65, 141.61, 136.86, 133.20, 133.13, 132.02, 131.41, 129.58, 129.06, 128.75, 128.34, 128.20, 127.96, 126.54, 126.47, 125.33, 118.59, 105.69, 55.15, 51.96, 50.76, 44.64, 39.20, 38.98, 35.29, 34.96, 32.74, 30.71, 29.13, 25.87, 25.74, 25.26, 18.64, 17.79 ppm, IR ν_max_/cm^–1^: 3332 (NH), 2945, 2928, 2903 (C_sp3_–H), 1734, 1651, (CO) 704, 666 (Sb–F). MALDI MS *m*/*z* (%): 921.842 (100) [M–SbF_6_]^+^. Anal. Calcd, (%) for C_44_H_56_AuF_6_NO_4_PSSb (1157.22): C, 46.01; H, 5.15; N, 1.19; S, 2.73. Found: C. 46,23; H, 5.33; N, 1.24; S, 2. 76.

##### [Au(L1)(JohnPhos)] (**1b**)

40% Yield, Pale-Yellow Solid, ^1^H NMR (400 MHz, CDCl_3_) ^1^H NMR (400 MHz, CDCl_3_): δ 9.26 (s, 1H), 7.83 (m, 1H), 7.64 (d, *J* = 7.9 Hz, 1H), 7.56 (m, 2H), 7.49 (m, 3H), 7.26 (m, 2H), 7.20 (m, 2H), 7.13 (d, *J* = 7.9 Hz, 1H), 7.05 (t, *J* = 7.7 Hz, 1H), 6.94 (d, *J* = 7.4 Hz, 1H), 6.88 (dd, *J* = 8.5, 1.1 Hz, 1H), 6.78 (t, *J* = 7.5, 7.5 Hz, 1H), 4.76 (q, *J* = 6.8 Hz, 1H), 3.80 (s, 3H), 3.04 (m, 2H), 2.47 (s, 3H), 2.37 (m, 2H), 2.31 (s, 3H), 2.16 (s, 3H), 1.57 (s, 3H), 1.38 (dd, *J* = 16.2, 4.1 Hz, 18H). ^31^P{^1^H} NMR (162 MHz, CDCl_3_): δ 63.58 ppm. ^13^C{^1^H} NMR (101 MHz, CDCl_3_) δ: 169.70, 148.98, 148.86, 147.60, 139.43, 138.05, 132.92, 132.55, 131.38, 131.16, 129.90, 128.99, 128.08, 125.85, 125.81, 121.45, 117.22, 115.16, 114.73, 52.92, 51.49, 36.10, 35.77, 31.70, 31.09, 29.37, 26.52, 26.39, 26.23, 25.61, 20.64, 13.93 ppm. IR ν_max_/cm^–1^: 3417, 3276 (NH); 2987, 2971, 2900, (C_sp3_–H), 1740, 1639, (CO) 705, 655(Sb–F). MALDI MS *m*/*z* (%): 880.851 (100) [M–SbF_6_]^+^. Anal. Calcd, (%) for C_41_H_53_AuF_6_N_2_O_3_PSSb (1116.21): C, 44.06; H, 4.78; N, 2.51; S, 2.87. Found: C, 44.29; H, 4.92; N, 2.56; S, 2.98.

##### [Au(L2)(JohnPhos)] (**2b**)

66% yield, pale-yellow solid ^1^H NMR (400 MHz, DMSO-*d*_6_): δ 8.41 (d, *J* = 7.8 Hz, 1H), 8.03 (m, 1H), 7.69 (m, 2H), 7.52 (m, 3H), 7.31 (m, 1H), 7.26 (m, 1H), 7.22 (d, *J* = 8.1 Hz, 2H), 7.09 (d, *J* = 8.1 Hz, 2H), 4.37 (td, *J* = 8.6, 8.4, 4.9 Hz, 1H), 3.65 (q, *J* = 7.0, Hz, 1H), 3.58 (s, 3H), 2.85 (m, 2H), 2.40 (s, 3H), 2.02 (m, 2H), 1.80 (hept, *J* = 6.7 Hz, 1H), 1.38 (d, *J* = 16.1 Hz, 18H), 1.33 (d, *J* = 7.1 Hz, 3H), 0.86 (d, *J* = 6.6 Hz, 6H). ^31^P{^1^H} NMR (162 MHz, DMSO-*d*_6_) δ: 62.5 ppm. ^13^C NMR (101 MHz, DMSO-*d*_6_) δ: 173.94, 171.66, 148.65, 148.52, 142.67, 142.60, 139.47, 139.17, 134.34, 132.89, 132.82, 131.74, 129.72, 129.10, 129.07, 128.94, 128.14, 128.07, 127.93, 127.22, 124.39, 123.93, 52.17, 50.83, 44.54, 44.42, 38.03, 37.79, 33.59, 31.15, 31.08, 30.55, 30.53, 30.49, 30.47, 29.82, 22.26, 18.90 ppm. IR ν_max_/cm^–1^: 3335 (NH), 2971, 2921, (C_sp3_–H), 1732, 1639, (CO) 703, 654 (Sb–F). MALDI MS *m*/*z* (%): 880.828 (100) [M–SbF_6_]^+^. Anal. Calcd, (%) for C_39_H_56_AuF_6_NO_3_PSSb (1081.23): C, 43.27; H, 5.21; N, 1.29; S, 2.96. Found: C, 43.51; H, 5.43; N, 1.33; S, 3.10.

##### [Au(L3)(JohnPhos)] (**3b**)

56% yield, yellow solid, ^1^H NMR (400 MHz, CDCl_3_): δ 7.85 (m, 1H), 7.67 (m, 2H), 7.58 (q, *J* = 7.0, 7.0, 6.8 Hz, 1H), 7.48 (m, 3H), 7.46 (s, 1H), 7.30 (m, 1H), 7.20 (dd, *J* = 6.5, 2.9 Hz, 2H), 7.01 (d, *J* = 2.5 Hz, 1H), 6.95 (d, *J* = 9.0 Hz, 1H), 6.80 (m, 1H), 6.66 (dd, *J* = 9.0, 2.5 Hz, 1H), 4.56 (m, 1H), 3.84 (s, 2H), 3.69 (s, 2H), 3.67 (s, 2H), 2.92 (m, 2H), 2.37 (s, 3H), 2.19 (d, *J* = 12.1 Hz, 2H), 1.59 (s, 3H), 1.41 (dd, *J* = 16.2, 1.6 Hz, 18H).ppm. ^31^P{^1^H} NMR (162 MHz, CDCl_3_): δ = 63.55 ppm, ^13^C{^1^H} NMR (101 MHz. CDCl_3_): δ 170.89, 168.41, 156.16, 148.98, 148.86, 142.72, 142.66, 139.14, 136.27, 134.04, 133.52, 133.49, 133.32, 133.24, 131.53, 131.20, 130.97, 130.67, 129.82, 129.79, 129.11, 128.11, 127.68, 127.61, 124.73, 124.28, 115.05, 113.07, 111.97, 101.19, 55.82, 52.71, 51.31, 38.45, 38.21, 31.80, 31.51, 30.97, 30.95, 30.91, 30.88, 13.45 ppm. IR ν_max_/cm^–1^: 3401 (NH), 2973, 2928 (C_sp3_–H), 1738, 1675, (CO) 704, 655(Sb–F). MALDI MS *m*/*z* (%): 997.2840 (100%). Anal. Calcd, (%) for C_45_H_54_AuClF_6_N_2_O_5_PSSb (1232.17): C, 43.80; H, 4.41; N, 2.27; S, 2.60. Found: C, 43.93; H, 4.62; N, 2.34; S,2.82.

##### [Au(L4)(JohnPhos)] (**4b**)

56% yield, white solid, ^1^H NMR (400 MHz, DMSO-*d*_6_): δ 8.47 (d, *J* = 7.7 Hz, 1H), 8.02 (m, 1H), 7.77 (dd, *J* = 8.7, 7.0 Hz, 2H), 7.72 (d, *J* = 1.4 Hz, 1H), 7.68 (m, 2H), 7.51 (m, 3H), 7.44 (dd, *J* = 8.6, 1.8 Hz, 1H), 7.29 (m, 1H), 7.24 (m, 2H), 7.15 (dd, *J* = 8.9, 2.6 Hz, 1H), 4.38 (td, *J* = 8.7, 8.5, 4.9 Hz, 1H), 3.86 (s, 3H), 3.81 (q, *J* = 7.0, 7.0 Hz, 1H), 3.56 (s, 3H), 2.83 (m, 2H), 2.37 (s, 3H), 2.01 (m, 2H), 1.42 (d, *J* = 7.0 Hz, 3H), 1.36 (d, *J* = 16.1, 1.5 Hz, 18H). ^31^P{^1^H} NMR (162 MHz, DMSO-*d*_6_) δ = 62.53 ppm. ^13^C{^1^H} NMR (101 MHz, DMSO-*d*_6_) δ: 173.49, 171.28, 156.81, 148.22, 142.23, 142.17, 136.62, 133.93, 132.46, 132.38, 131.30, 129.28, 128.85, 128.64, 127.71, 127.50, 126.34, 126.26, 125.13, 118.36, 105.48, 54.93, 51.76, 50.50, 44.45, 37.59, 37.34, 32.94, 30.61, 30.11, 18.40, 18.18 ppm. IR ν_max_/cm^–1^: 3401 (NH), 2957,2861 (C_sp3_–H), 1741, 1676, (CO) 705, 657(Sb–F), ESI-MS *m*/*z* (%): 870.304 (5.21) [M–SbF_6_]^+^. Anal. Calcd, (%) for C_40_H_52_AuF_6_NO_4_PSSb (1106.60): C, 43.80; H, 4.41; N, 2.27; S, 2.60. Found: C, 43.63; H, 4.81; N, 1.31; S, 2.73.

### Solution Chemistry

#### Solution Stability

The stability of the gold complexes in solution was analyzed by UV–vis absorption spectroscopy. UV–vis absorption spectra of the complexes were recorded on a Thermo Scientific Evolution 600 spectrophotometer. First, stock solutions of the new complexes were prepared at 10 mM in DMSO. From these working solutions (10 mL) were prepared at 50 μM in PBS at pH = 7.4. The samples were then incubated at 37 °C and thereafter monitored by measuring the electronic spectra over 24 h.

#### Stability in Solution in the Presence of a Reducing Agent/NAC

^1^H NMR spectra were recorded in a mixture of DMSO-*d*_6_:D_2_O (80:20) containing the gold complexes (0.01 mmol) with an equimolecular amount of NAC, The corresponding NMR spectra were recorded immediately after preparation, at time 0 and after 72 h.

#### Reaction with GSH

^1^H NMR spectra were recorded in a mixture of DMSO-*d*_6_:D_2_O (80:20) containing the gold complexes **1b**, **1b** and **4b** (0.01 mmol) with an equimolecular amount of GSH, The corresponding NMR spectra were recorded immediately after preparation, at time 0 and after 48 h.

#### UV–Vis Absorption Spectroscopy

UV–Vis spectra were recorded on a JASCO V-780 UV–Vis spectrophotometer in the range of 200–800 nm. A 7.5 mM stock solution of the gold complex **4b** was prepared by dissolving the complex in DMSO. The electronic spectra were recorded diluting a small amount of this freshly prepared stock solution and the relative amount of BSA at a stoichiometric ratio of 1:1 (metal-to-protein). The final concentration of the protein and complexes after dilution in phosphate buffered solution, pH 7.4 was 5 × 10^–5^ M. The resulting solution was monitored collecting the electronic spectra over 24 h at room temperature.

#### BSA Quenching Experiments

Gold complexes were dissolved in DMSO to achieve 7.5 mM stock solutions and aliquots of 10 or 20 μL were added to a 50 μM solution of BSA in PBS placed in a quartz cuvette of 1 cm optical path. The final concentrations of gold complexes in the cuvette were 25, 50, 100, 150, 200, 250, and 300 μM. The fluorescence spectra were recorded on a Jobin-Yvon-Horiba fluorolog FL-3-11 spectrometer. The samples were excited at 295 nm and the emission spectra were recorded in a range from 310 to 450 nm with emission slits set to 2 nm. The fluorescence was measured 4 min after every addition of the aliquots of gold complexes.

### Cell Culture

The human Caco-2 cell line (TC7 clone) was kindly provided by Dr. Edith Brot-Laroche (Université Pierre et Marie Curie-Paris 6, UMR S 872, Les Cordeliers, France), Human epithelial fibroblast cells (NHDF-Ad, Lonza, Porriño, Spain) were kindly provided by Dr. Gracia Mendoza (Aragon Health Research Institute, IIS Aragón, Spain). Caco-2 cells (passages 36–48) and fibroblasts (passages 10–20) were maintained in a humidified atmosphere of 5% CO_2_ at 37 °C in Dulbecco’s Modified Eagles medium (DMEM) (Gibco Invitrogen. Paisley. UK) supplemented with 20% fetal bovine serum, 1% nonessential amino acids, 1% penicillin (1000 U/mL), 1% streptomycin (1000 mg/mL) and 1% amphotericin (250 U/mL). The Caco-2 cells were subcultured in 25 cm^2^ plastic flasks at a density of 3 × 10^5^ cells/cm^2^, while the fibroblasts were subcultured on 75 cm^2^ plastic flasks at a density of 1 × 10^4^ cells/cm^2^. The culture medium was replaced every 2 days.

### Cell Viability Assay

The gold complexes were diluted in dimethyl sulfoxide (DMSO) as a 10 mM stock solution and then diluted in a cell culture medium at the desired concentrations. For the cytotoxicity screening assays, Caco-2 cells were seeded in 96-well plates at a density of 4 × 10^3^ cells/well. The culture medium was replaced with a medium containing the drug panel 24 h postseeding, and the cells were incubated for 72 h. An initial range of complex concentrations of 0.097–50 μM in undifferentiated Caco-2 to determine the IC_50_ values, fibroblasts, and culture medium containing metal gold complexes was added 24 h postseeding, and the cells were incubated for 72 h. The antiproliferative effect was measured using an MTT assay, as previously described by Marmol et al.^93^ The absorbance at 540/620 nm was measured using a SPECTROstar Nano (BMG Labtech. Ortenberg. Germany). To determine the selectivity index (SI), the IC_50_ value of the fibroblast cells was divided by the IC_50_ value in undifferentiated Caco-2 obtaining the ratio of the normal/cancerous cells’ toxicity.

### Intracellular TrxR Activity

The Caco-2 cell line (TC7 clone) was seeded in a 25 cm^2^ flask (2 × 10^6^). After 24 h incubation, the IC_50_ concentration of the compounds was added for 24 h. Then the cells were collected and lysated by M-PER Mammalian Protein Extraction Reagent Thermo Fisher Scientific, 78501) following the supplieŕs instructions. The interaction of metal complexes with the enzyme thioredoxin reductase was analyzed using a thioredoxin reductase assay kit (Sigma, CS0170, St Louis, MO, USA). The procedural guidelines of the supplied kit were followed. The reaction started with the introduction of DTNB (100 mM), and the transformation into TNB was observed at 412 nm at 30 s intervals over 5 min, utilizing a SPECTROstar Nano multiplate reader from BMG Labtech in Ortenberg, Germany. The outcomes were presented as a percentage representing the TrxR activity relative to the control.

### Intracellular COX-1/COX-2 Activity

The Caco-2 cell line (TC7 clone) was seeded in a 25 cm^2^ flask (2 × 10^6^). After 24 h incubation, IC_50_ concentration of the compounds was added for 24 h. Then the cells were collected and lysated by M-PER Mammalian Protein Extraction Reagent (Thermo Fisher Scientific, 78501) following the supplieŕs instructions. The interaction of metal complexes with the enzyme cyclooxygenase was analyzed using a cyclooxygenase (COX) Activity Assay Kit (fluorometric) (AB204699 Abcam Inc. Massachusetts, USA). The procedural guidelines of the supplied kit were followed. The reaction commenced with the introduction of AA. The transformation in prostaglandin G2 was observed at (Ex/Em = 535/587) at 5 min intervals over a span of 90 min utilizing a FLUOstar Omega (BMG Labtech. Ortenberg. Germany) multiplate.

### Measurement of Intracellular ROS Levels

Caco-2 cells were seeded in 96-well black plate at a density of 4 × 10^3^ cells/well and intracellular ROS levels were determined with the dichlorofluorescein assay. Cells were exposed to the drug for 24 h and then incubated with 20 μM 2′-7′-dichlorofluorescein diacetate (DCFH-DA) (Merck KGaA, Darmstadt, Germany) in DMEM. The generation of oxidized derivative 2′,7′-dichlorofluorescein (DCF) was monitored by measuring the increase of fluorescence for 1 h at an emission wavelength of 520 nm and excitation of 485 nm, with a FLUOstar Omega (BMG Labtech. Ortenberg. Germany) multiplate reader. Results were expressed as a percentage of fluorescence concerning control, considering fluorescence intensity as a reflection of intracellular ROS levels.

### Apoptosis Assay

Caco-2 cells were seeded in 25 cm^2^ flasks at a density of 1 × 10^6^ cells/cm^2^, then exposed to the 4 × IC_50_ drug concentration for 48 h. The cells were then transferred to flow cytometry tubes and washed twice with PBS; they were then resuspended in 100 μL annexin V binding buffer (100 mM Hepes/NaOH pH 7.4, 140 nM NaCl, 2.5 mM CaCl_2_). In total, 5 μL annexin V-FITC and 5 μL propidium iodide (PI) were added to the tube. After 15 min of incubation at room temperature protected from light, 400 μL annexin binding buffer was added to each sample, and the signal intensity was analyzed within 1 h with Beckman Coulter Gallios (Brea. CA. USA). The data were analyzed with BD FACSDivaTM.

### Statistical Analyses

All results are expressed as means ± SEM of at least three independent experiments. Statistical comparisons were performed using Student’s *t*-test or one-way ANOVA followed by the Bonferroni posttest and the differences between *P*-values <0.05 were considered statistically significant. Statistical analyses were carried out using the Prism GraphPad Program (Prism version 9.0, GraphPad Software, San Diego, CA).

## Supporting Information Available

RMN spectra of all the complexes and UV–visible spectra are included in the Supporting Information (PDF)
