# Supplementary material for: Gold(I) Complexes Based on Nonsteroidal Anti-Inflammatory Derivatives as Multi-Target Drugs against Colon Cancer
Source: Inorg Chem. 2024 Oct 10;63(42):19769–82. doi: 10.1021/acs.inorgchem.4c02988 (PMC11497205; doi:10.1021/acs.inorgchem.4c02988)
Supplement: Supplementary file 1 — ic4c02988_si_001.pdf [file ic4c02988_si_001.pdf]

Gold(I) Complexes Based on Nonsteroidal Anti-Inflammatory Derivatives as Multi-Target Drugs Against Colon Cancer

Javier Saez,[a] Javier Quero,[b] María Jesús Rodriguez-Yoldi,[b] M. Concepción Gimeno,[a] and Elena Cerrada,[a*]*

[a]Departamento de Química Inorgánica. Instituto de Síntesis Química y Catálisis Homogénea-ISQCH. Universidad de Zaragoza-C.S.I.C., 50009 Zaragoza, Spain, E-mail: ecerrada@unizar.es; gimeno@unizar.es

[b]Departamento de Farmacología y Fisiología. Unidad de Fisiología. Universidad de Zaragoza, 50013, Zaragoza, Spain. CIBERobn, IIS Aragón, IA2.

Table of contents

1 NMR spectra for ligands and complexes S1-S33.
2 Mass spectra of compounds S34-S44.
3 Stability assays S46-60.
4 Flow cytometry: S62.

1 NMR spectra for ligands and complexes S1-S33.

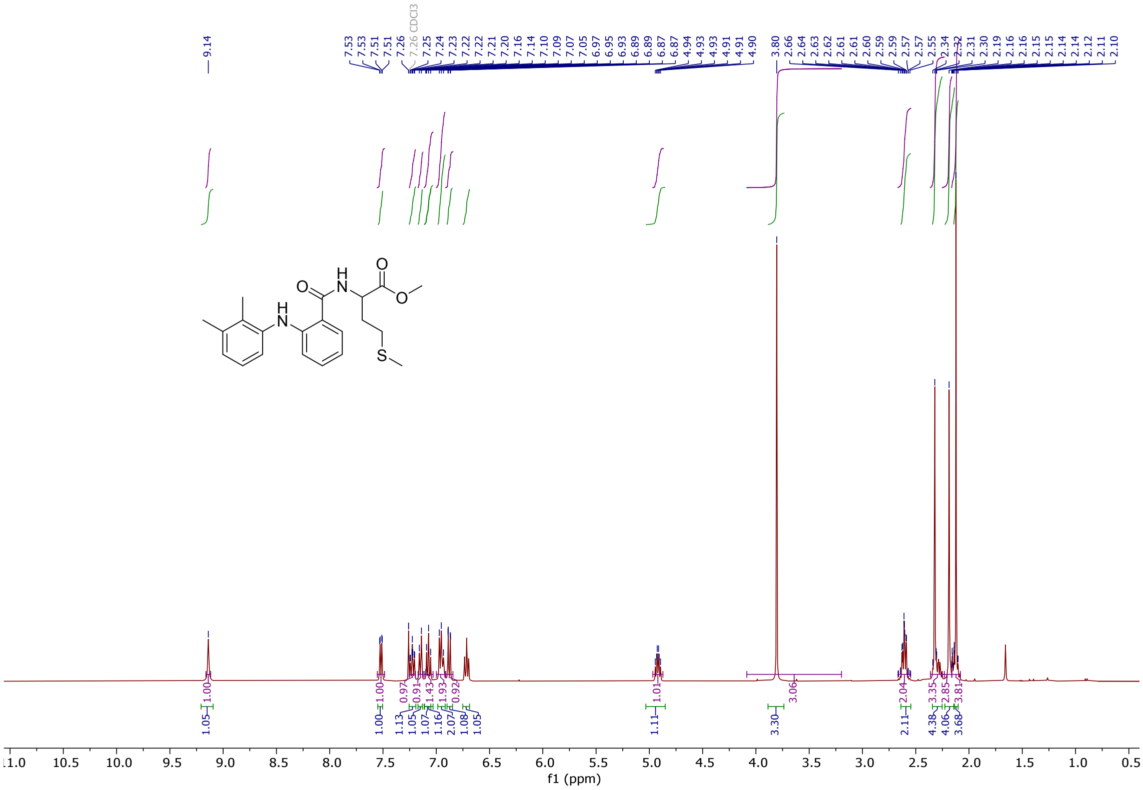

Figure S1. ^{1}H NMR spectrum (400 MHz, $CDCl_3$) of L1.

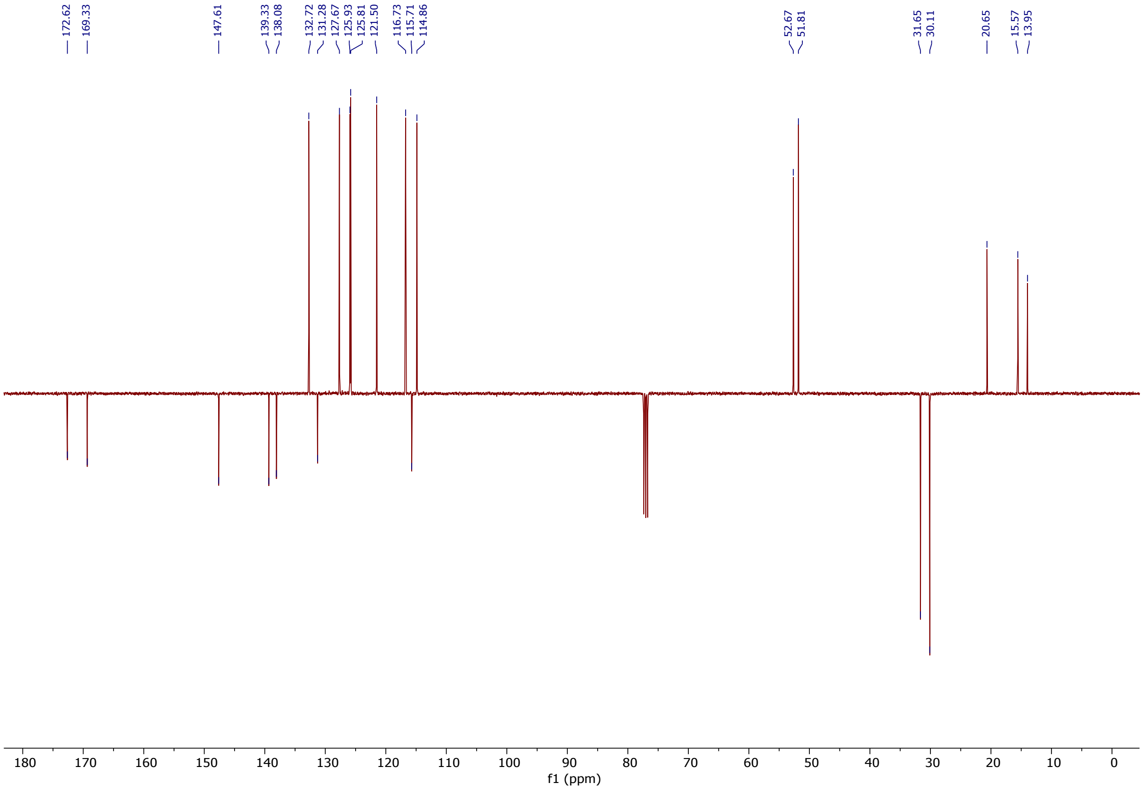

Figure S2. ^{13}C {^{1}H} NMR ((101 MHz, $CDCl_3$) spectrum of compound L1.

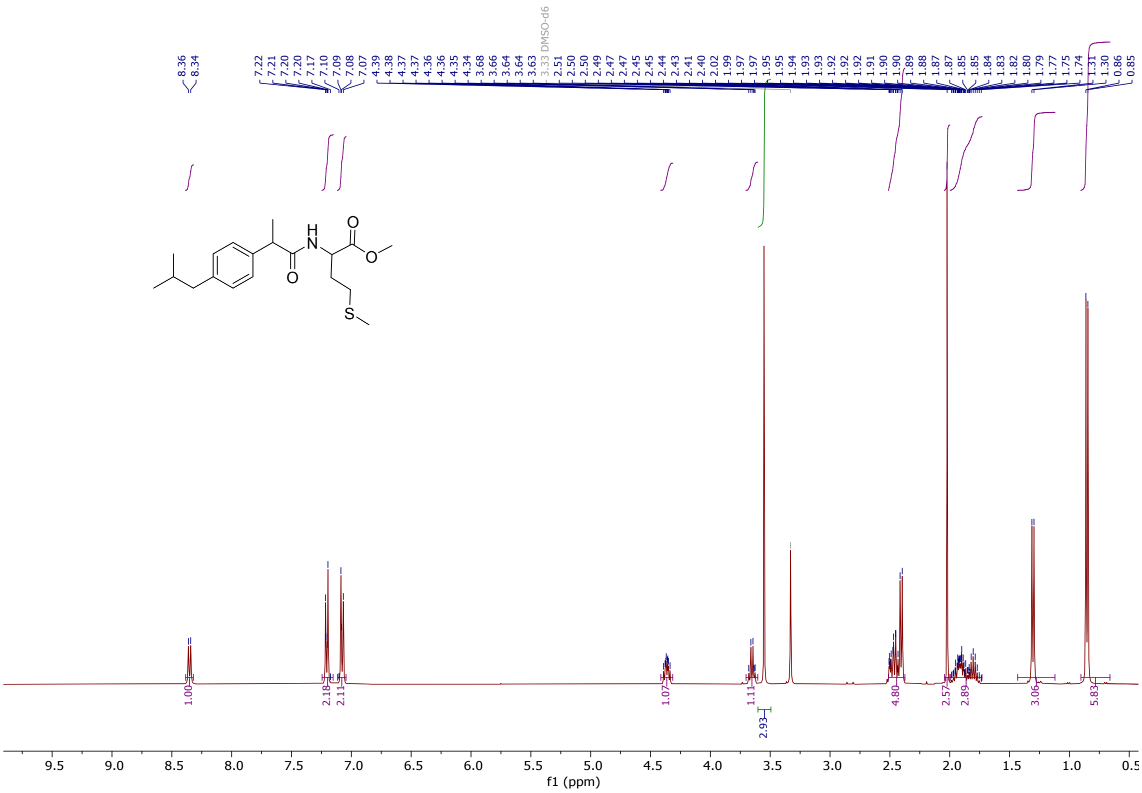

Figure S3. ^{1}H NMR spectrum (400 MHz, DMSO-d$_6$) of L2.

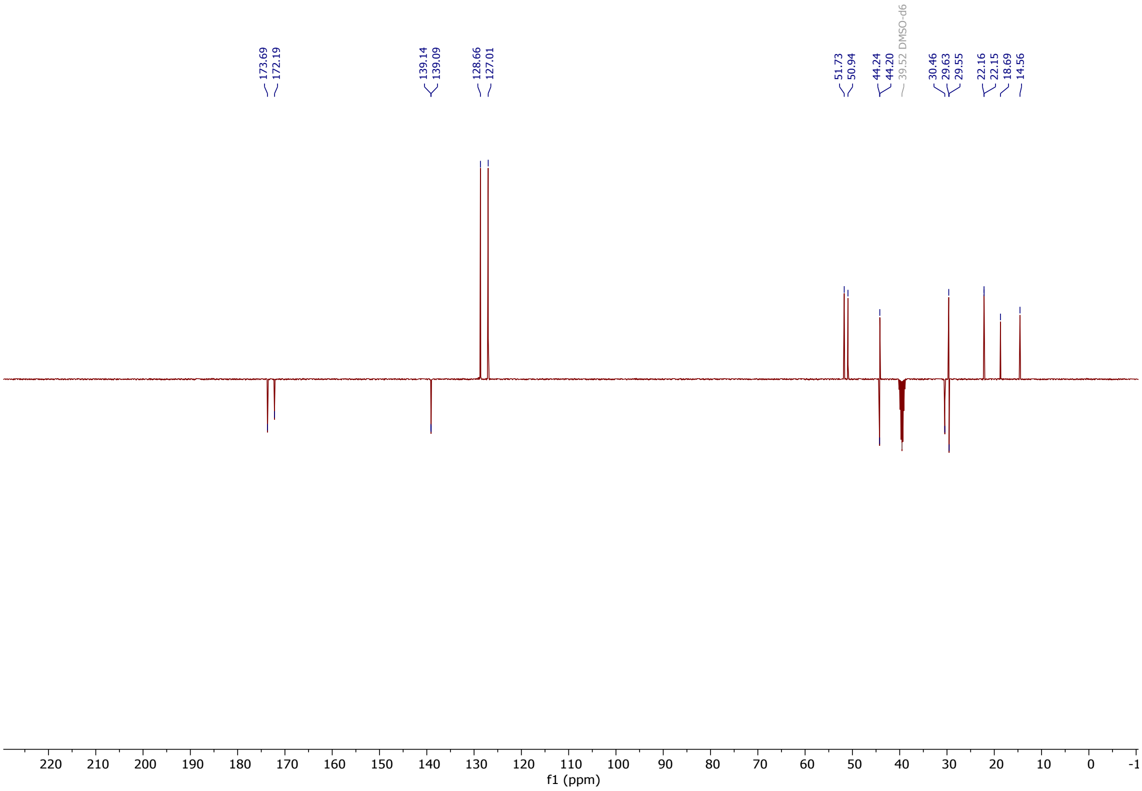

Figure S4. ^{13}C {^{1}H} NMR ((101 MHz, DMSO-d$_6$) spectrum of compound L2.

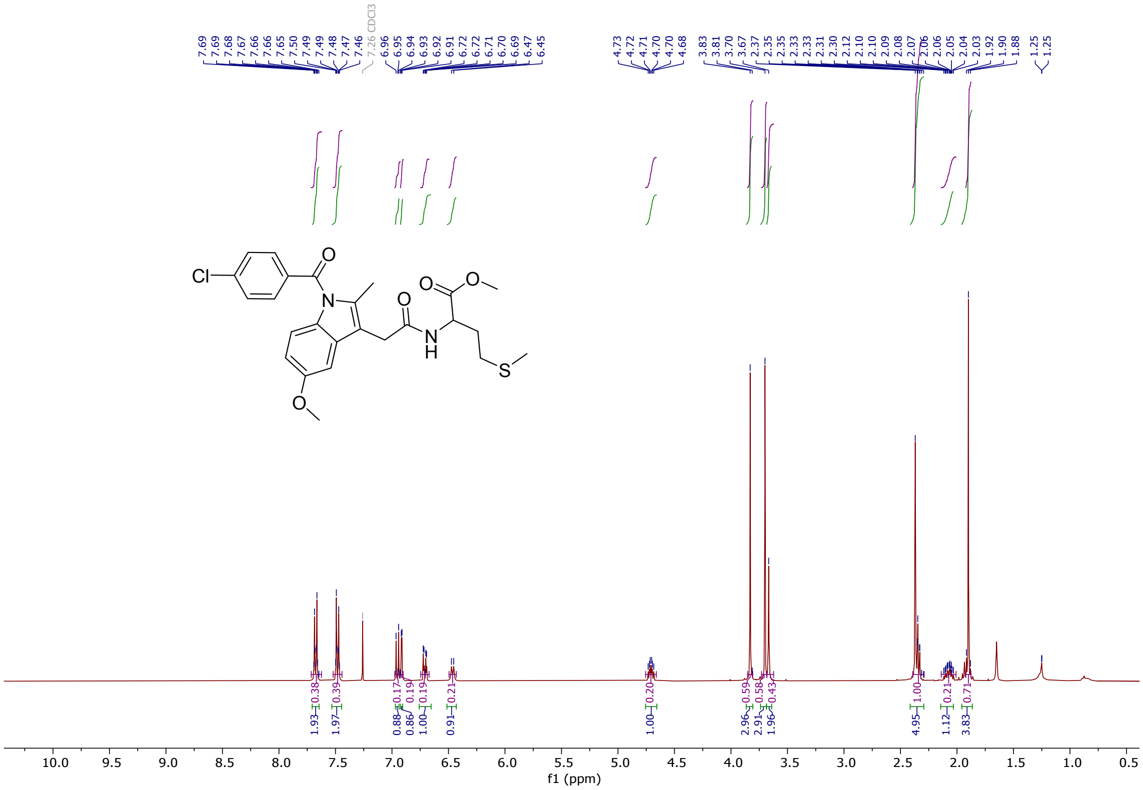

Figure S5 $^{13}C\ \{^1H\}$ NMR ((101 MHz, $CDCl_3$) spectrum of compound L3.

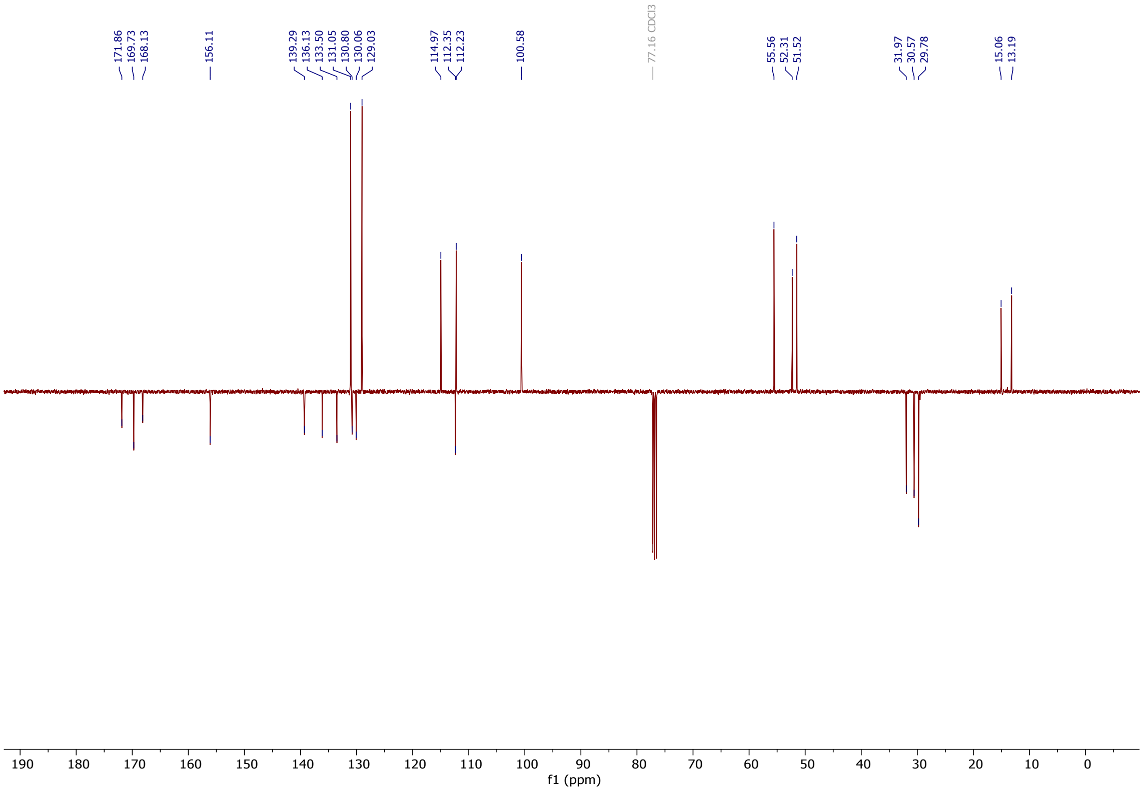

Figure S6. $^{13}C\ \{^1H\}$ NMR ((101 MHz, $CDCl_3$) spectrum of compound L3.

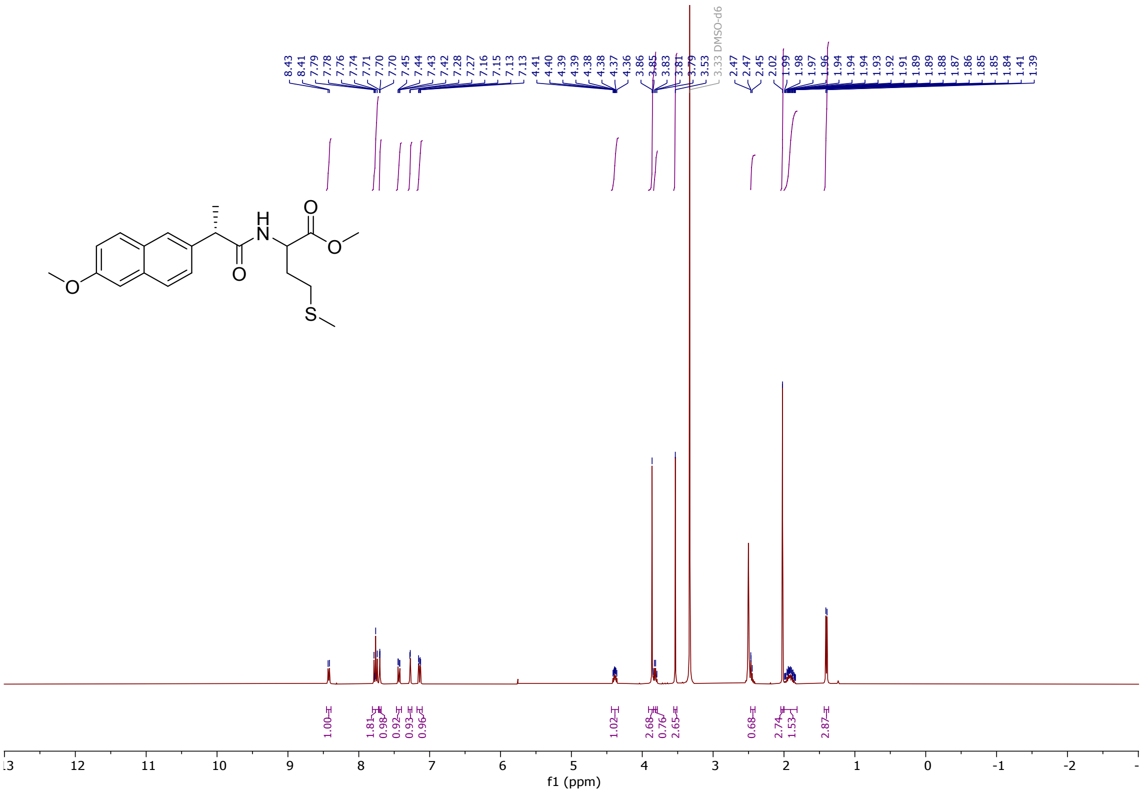

Figure S7. ^{1}H NMR spectrum (400 MHz, DMSO-d_6) of L4.

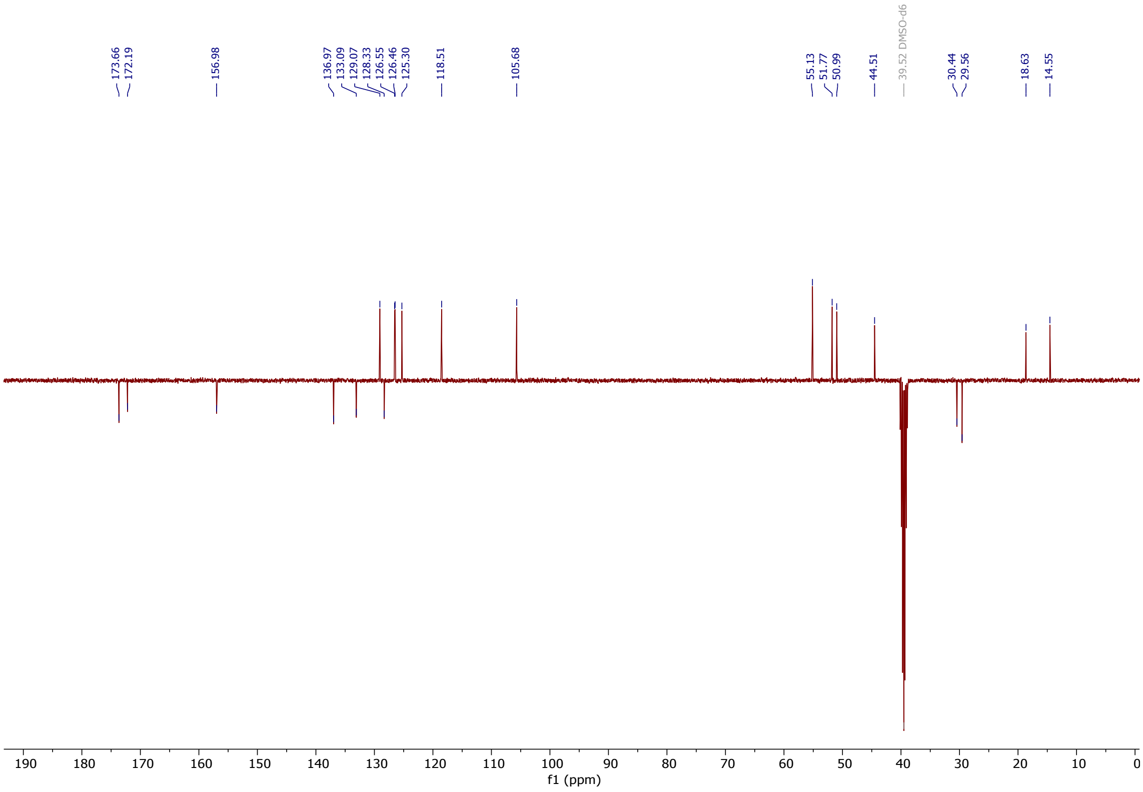

Figure S8. ^{13}C {^{1}H} NMR ((101 MHz, DMSO-d_6) spectrum of compound L4.

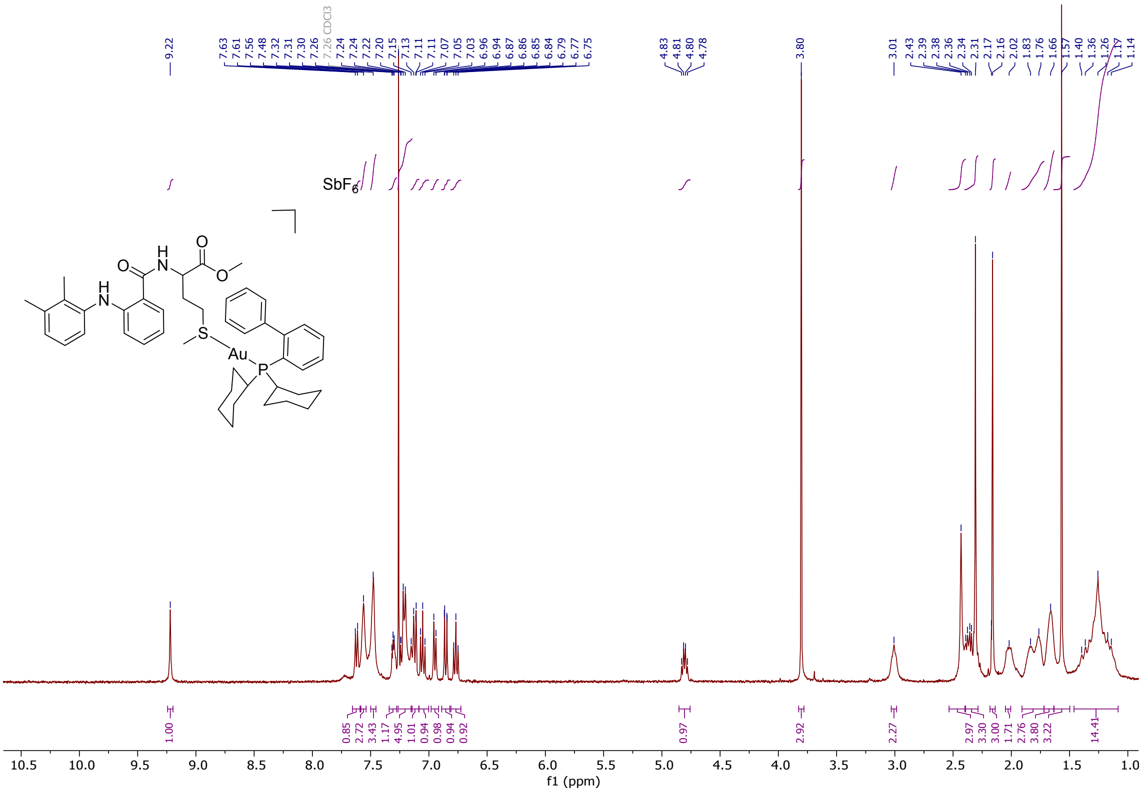

Figure S9. ^{1}H NMR spectrum (400 MHz, $CDCl_3$) of the complex 1a.

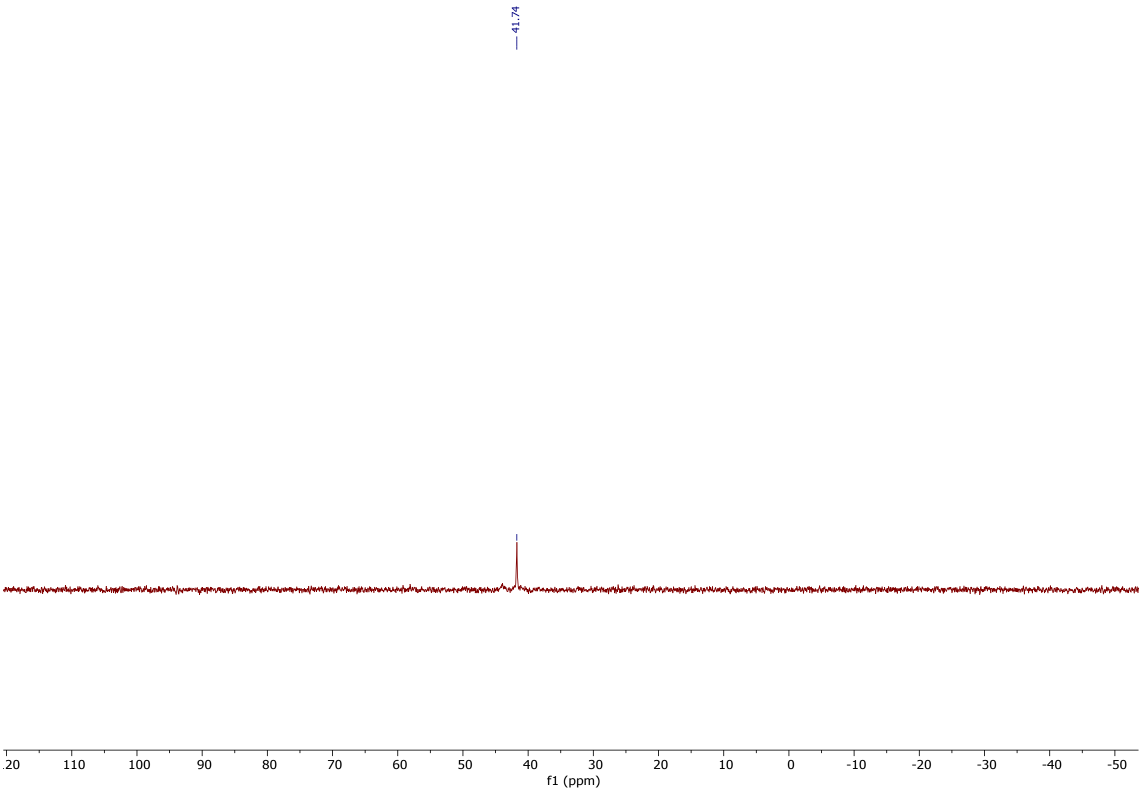

Figure S10. ^{31}P {^{1}H} NMR spectrum (162 MHz, $CDCl_3$) of the complex 1a.

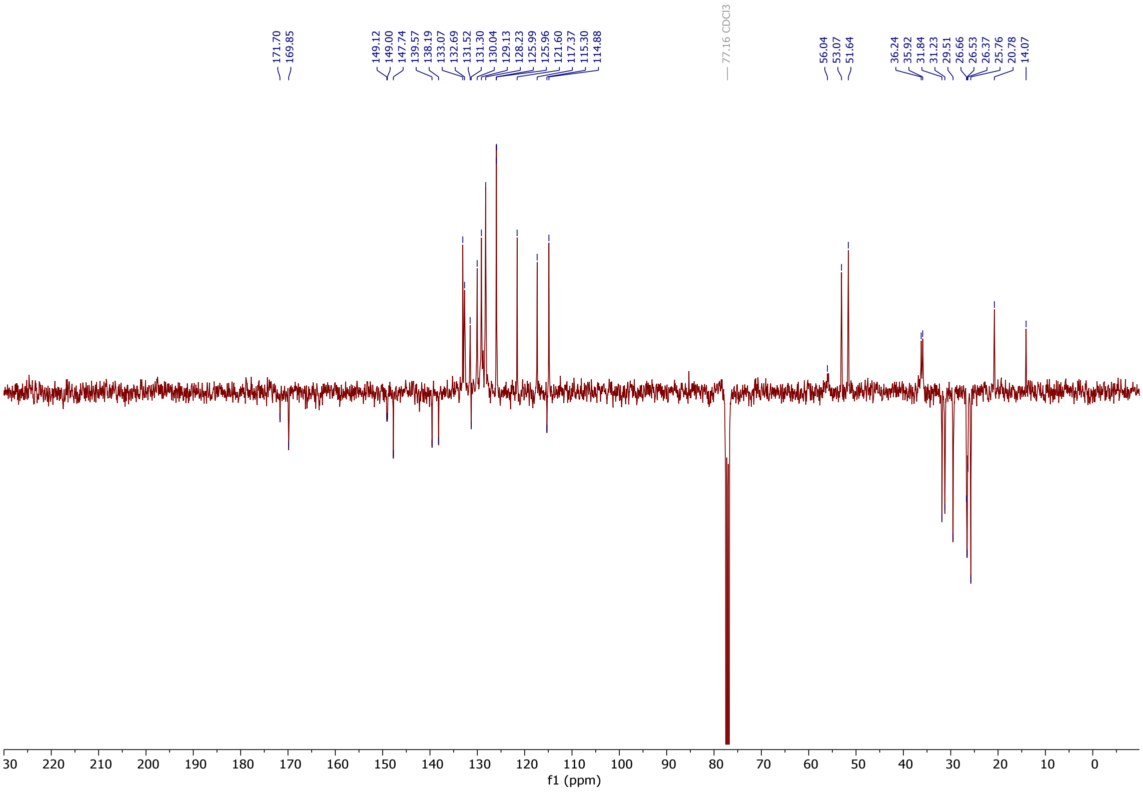

Figure S11 ^{13}C {^{1}H} NMR (101 MHz, $CDCl_3$) spectrum of compound 1a.

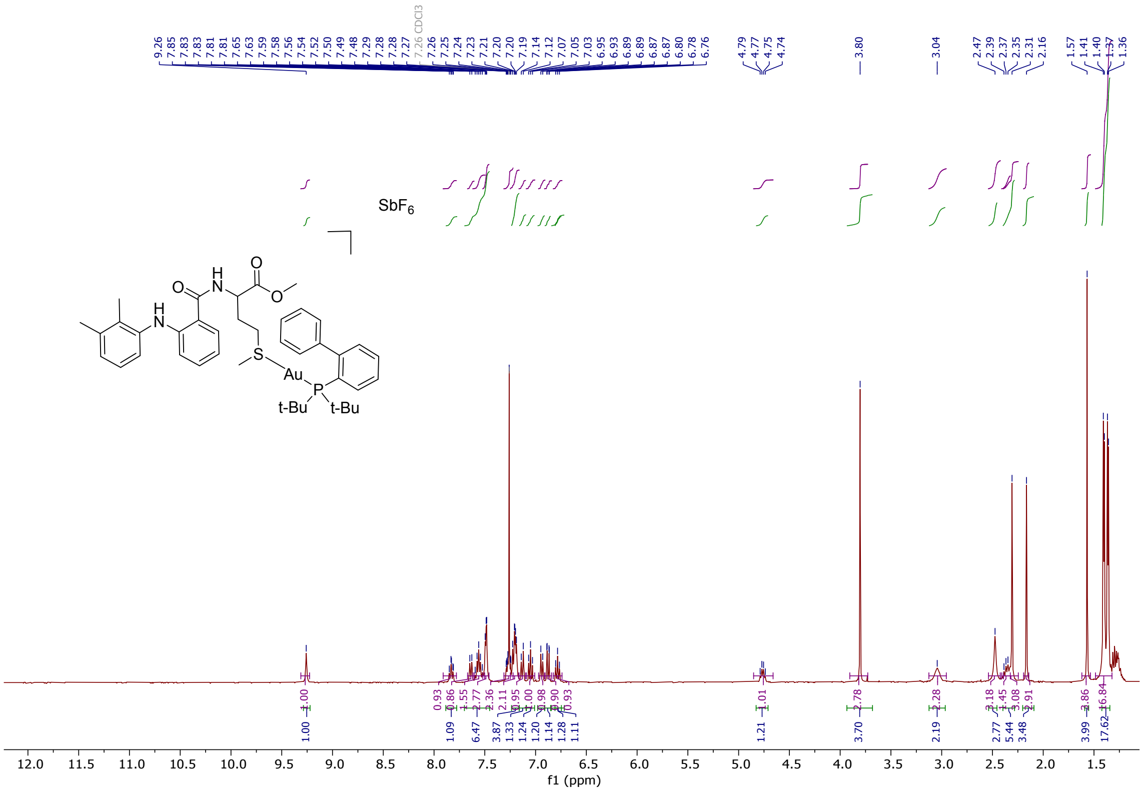

Figure S12. ^{1}H NMR spectrum (400 MHz, $CDCl_3$) of the complex 1b.

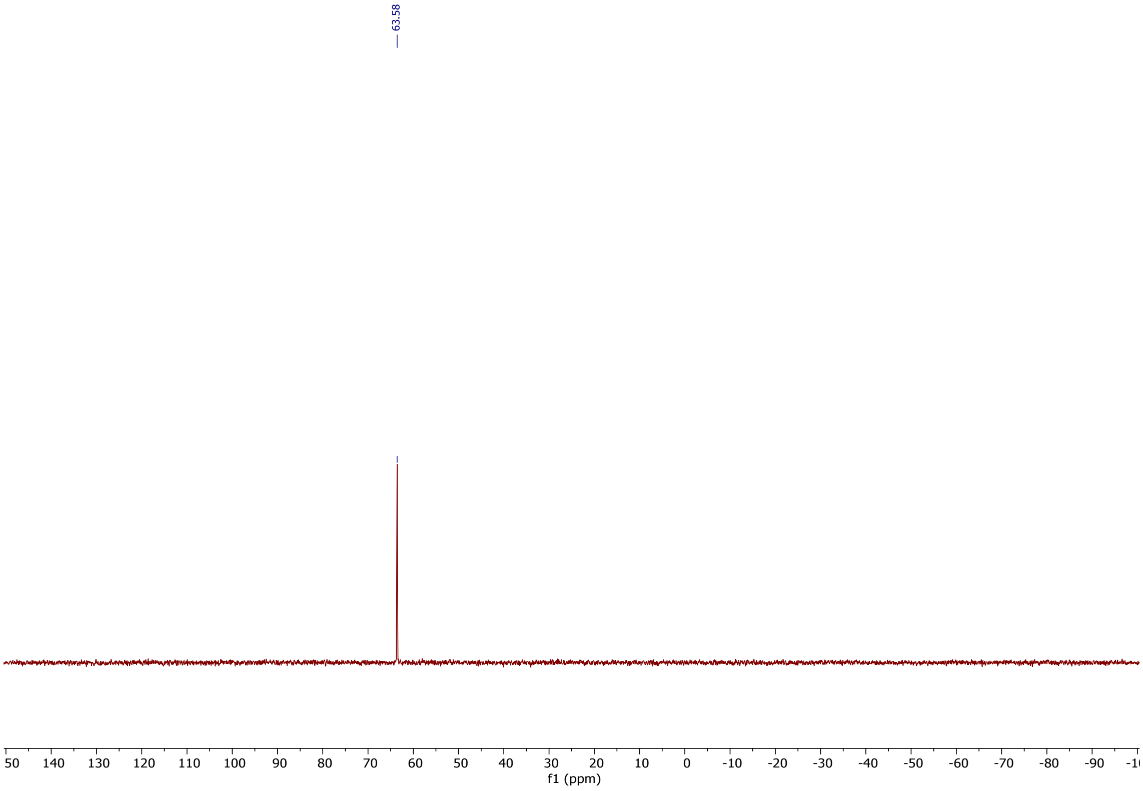

Figure S13. ^{31}P {^{1}H} NMR spectrum (162 MHz, $CDCl_3$) of the complex 1b

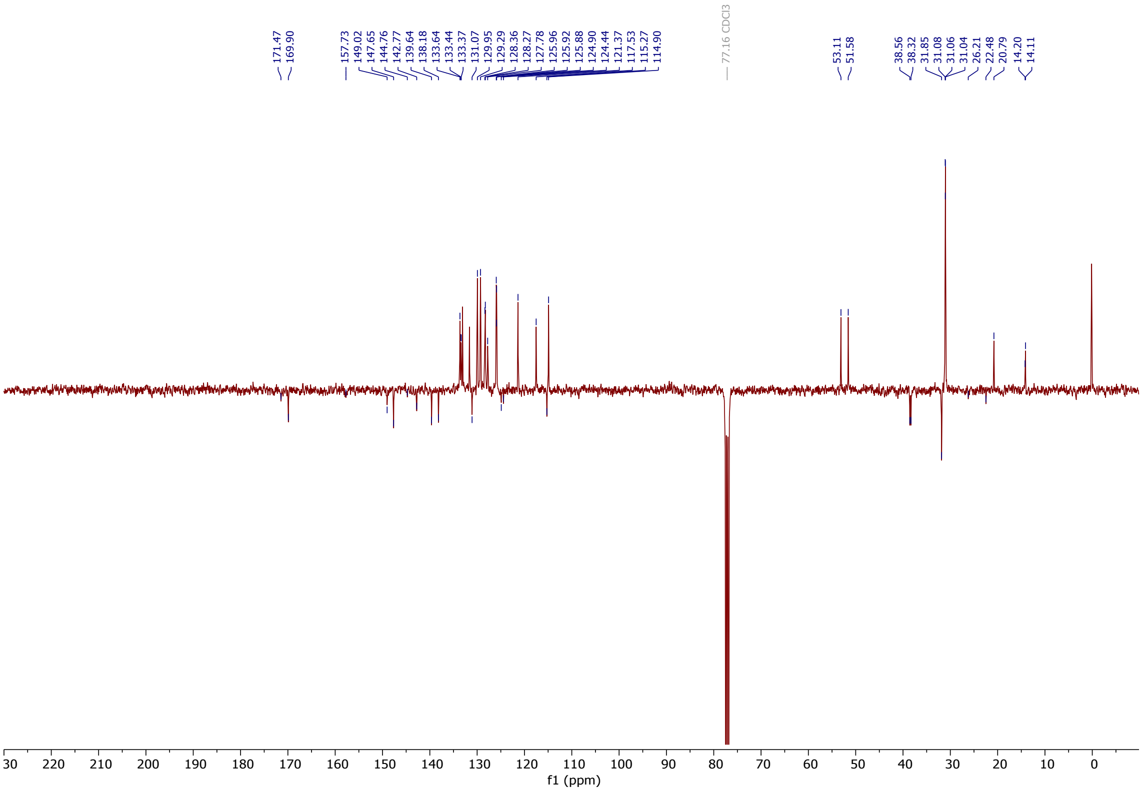

Figure S14 ^{13}C {^{1}H} NMR (101 MHz, $CDCl_3$) spectrum of compound 1b.

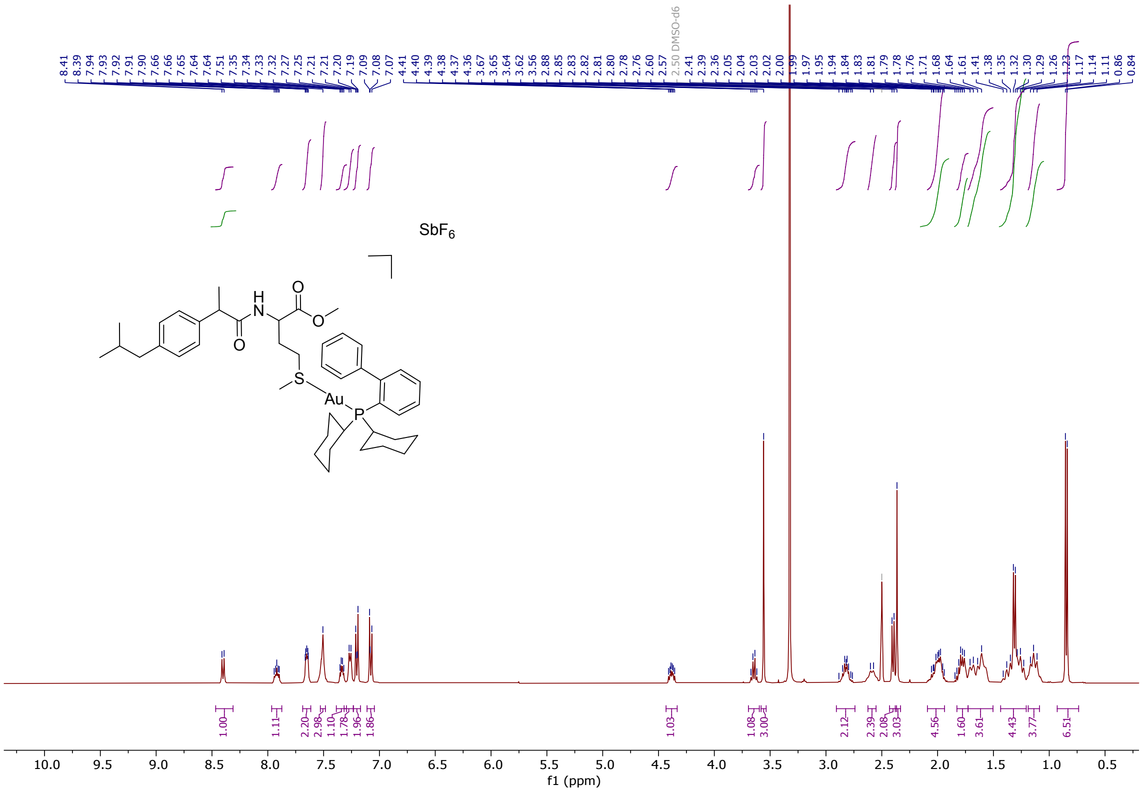

Figure S15. ^{1}H NMR spectrum (400 MHz, DMSO-d_6) of the complex 2a.

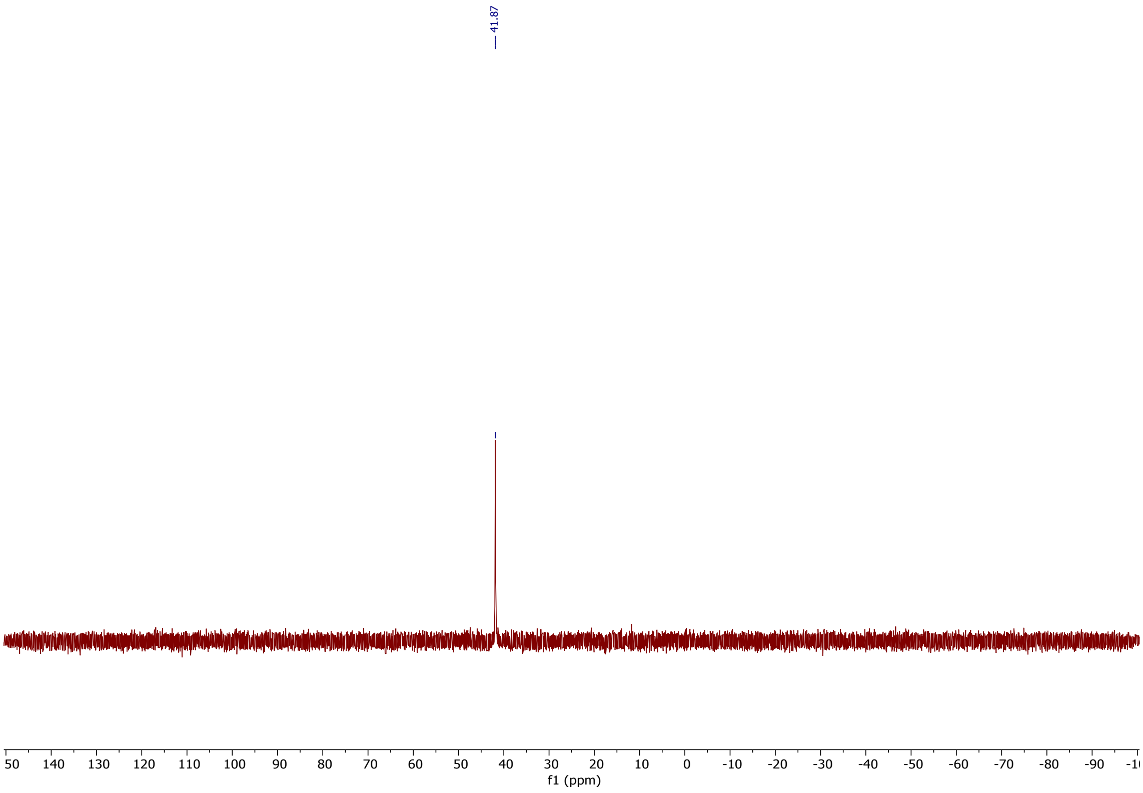

Figure S16. $^{31}P\ \{^{1}H\}$ NMR spectrum (162 MHz, DMSO-d_6) of the complex 2a.

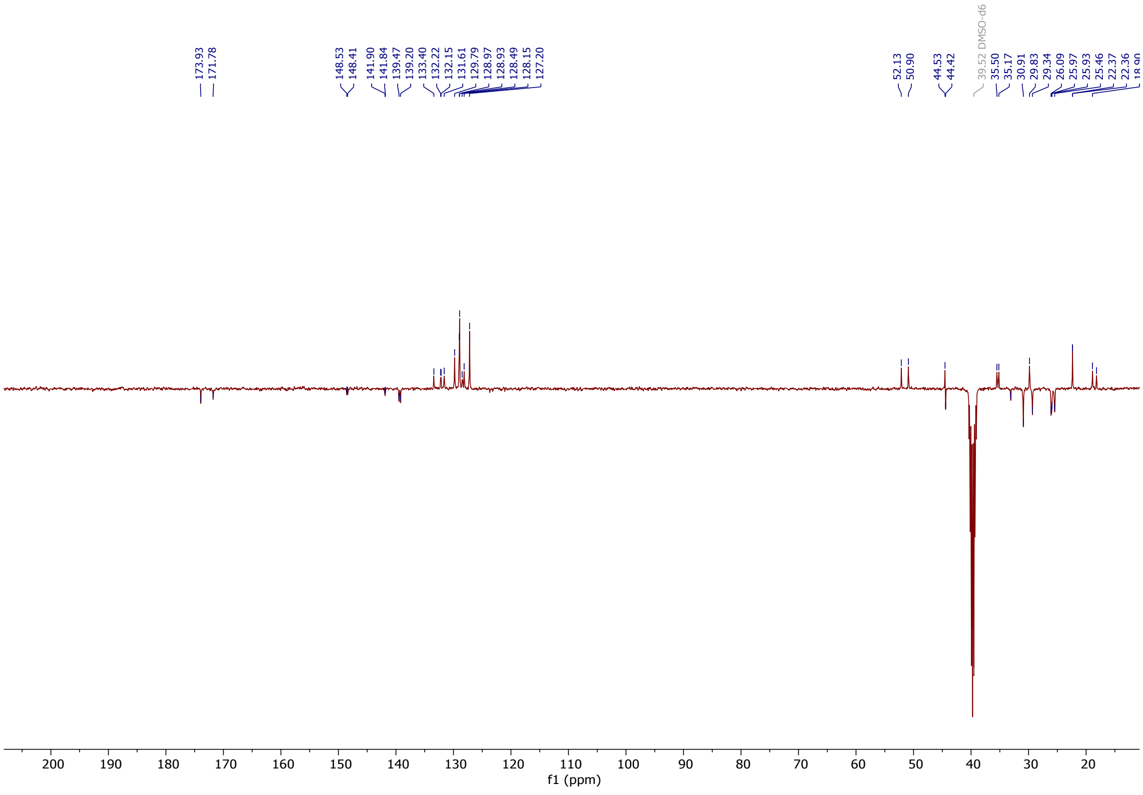

Figure S17. $^{13}C\ \{^{1}H\}$ NMR (101 MHz, DMSO-d_6) spectrum of compound 2a.

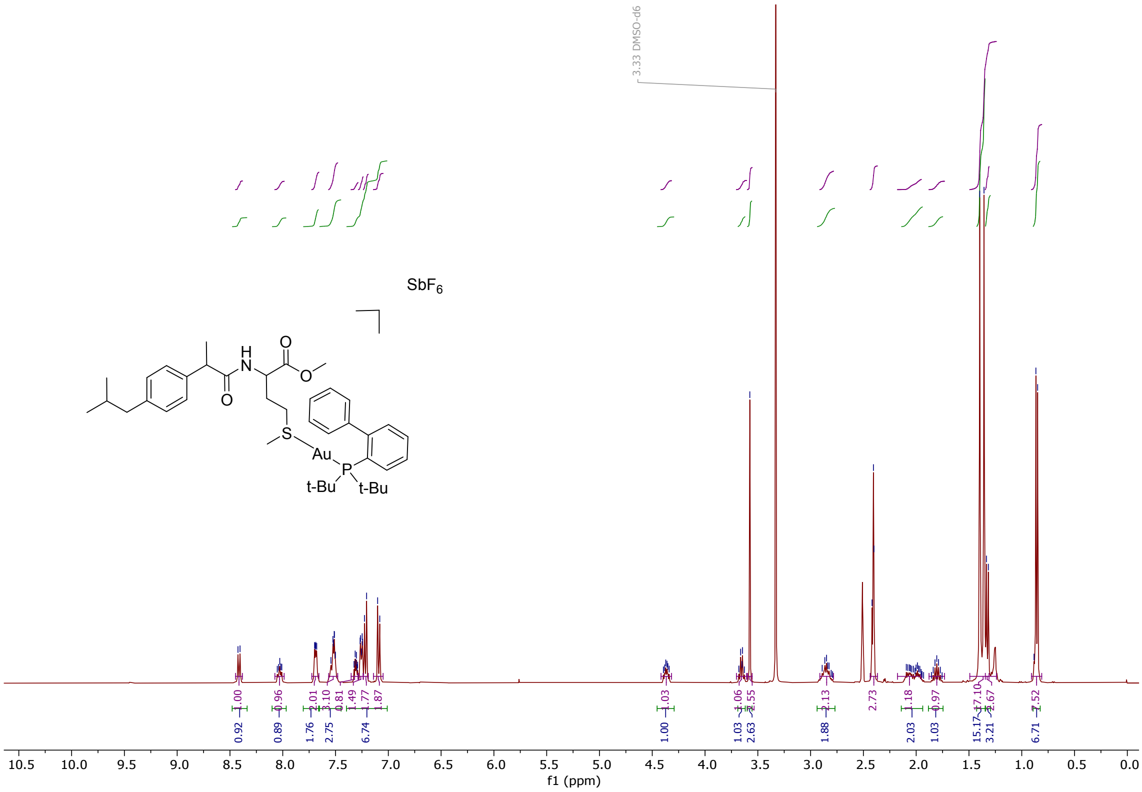

Figure S18. ^{1}H NMR spectrum (400 MHz, DMSO-d_6) of the complex 2b.

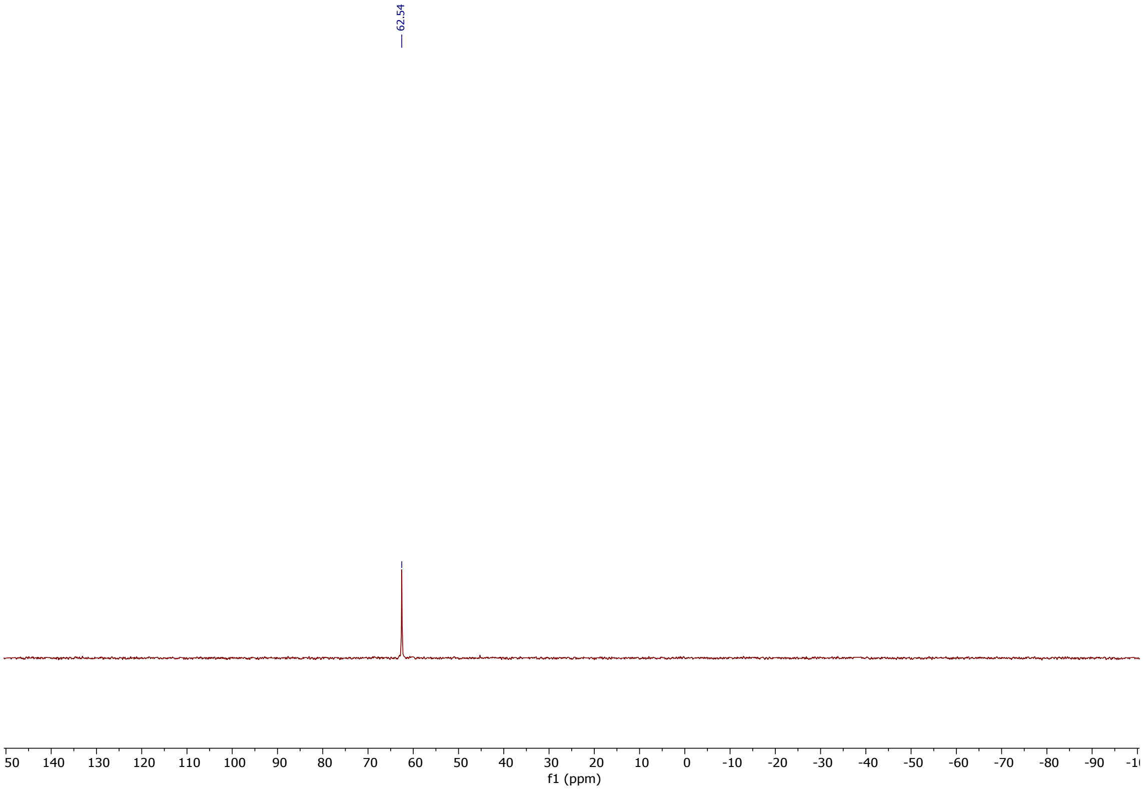

Figure S19. ^{31}P {^{1}H} NMR spectrum (162 MHz, DMSO-d_6) of the complex 2b.

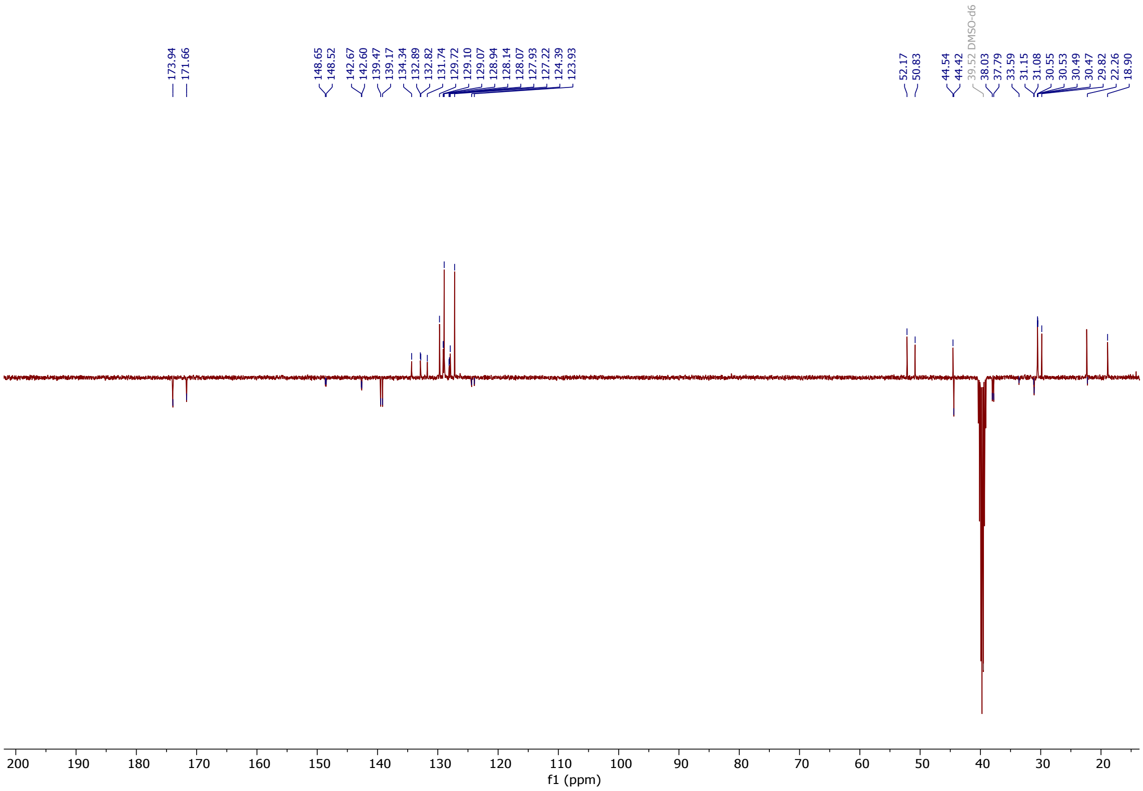

Figure S20. $^{13}C\ \{^{1}H\}$ NMR (101 MHz, DMSO-d$_6$) spectrum of compound 2b.

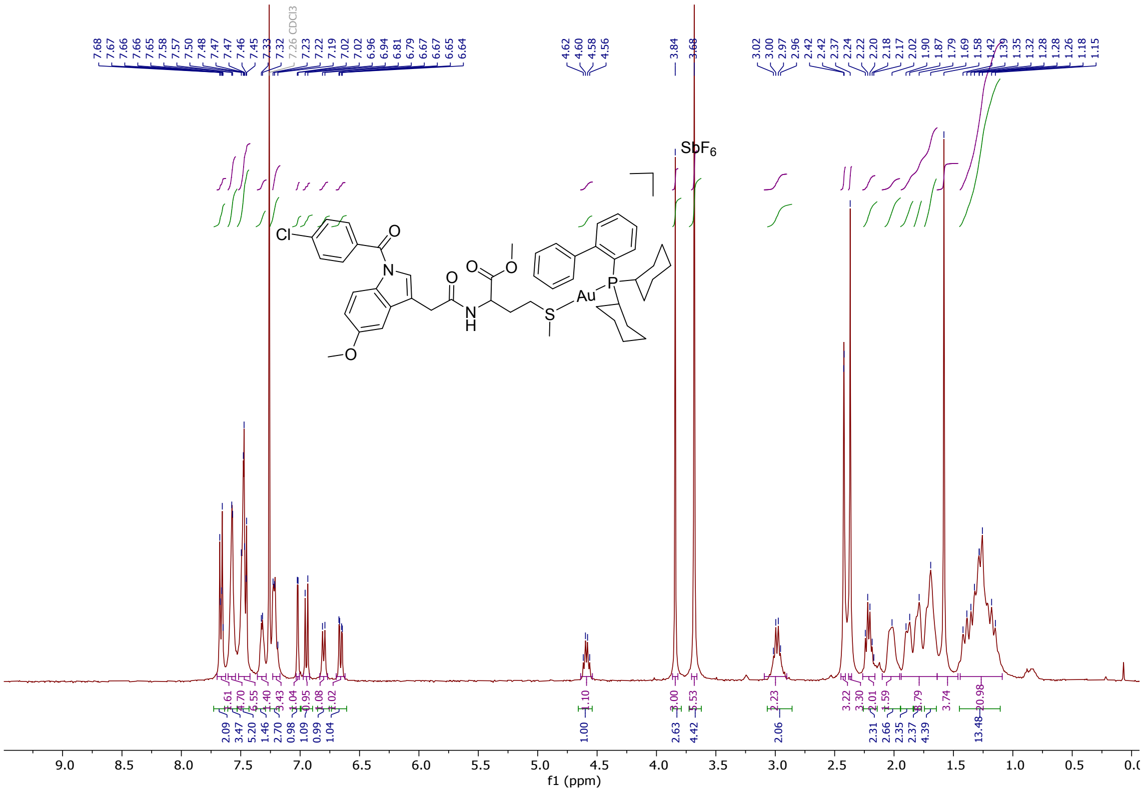

Figure S21. ^{1}H NMR spectrum (400 MHz, DMSO-d$_6$) of the complex 3a.

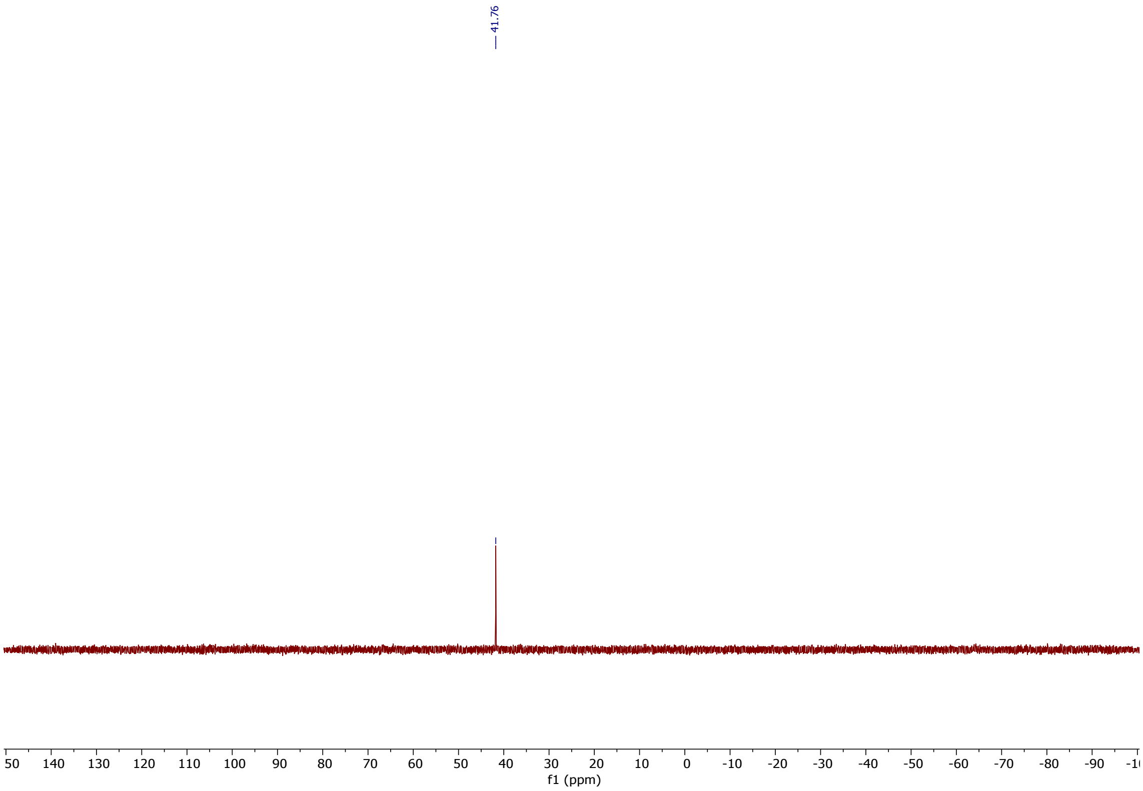

Figure S22. ^{31}P {^{1}H} NMR spectrum (162 MHz, DMSO-d_6) of the complex 3a.

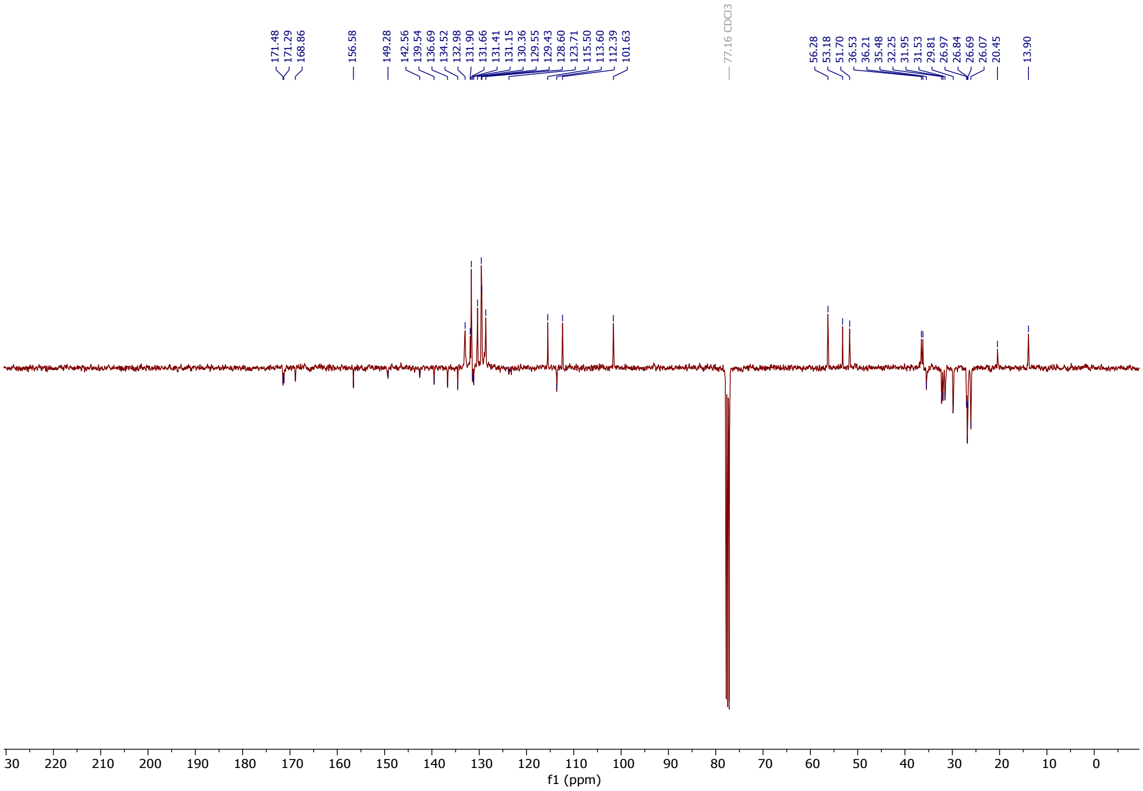

Figure S23 ^{13}C {^{1}H} NMR (101 MHz, DMSO-d_6) spectrum of compound 3a.

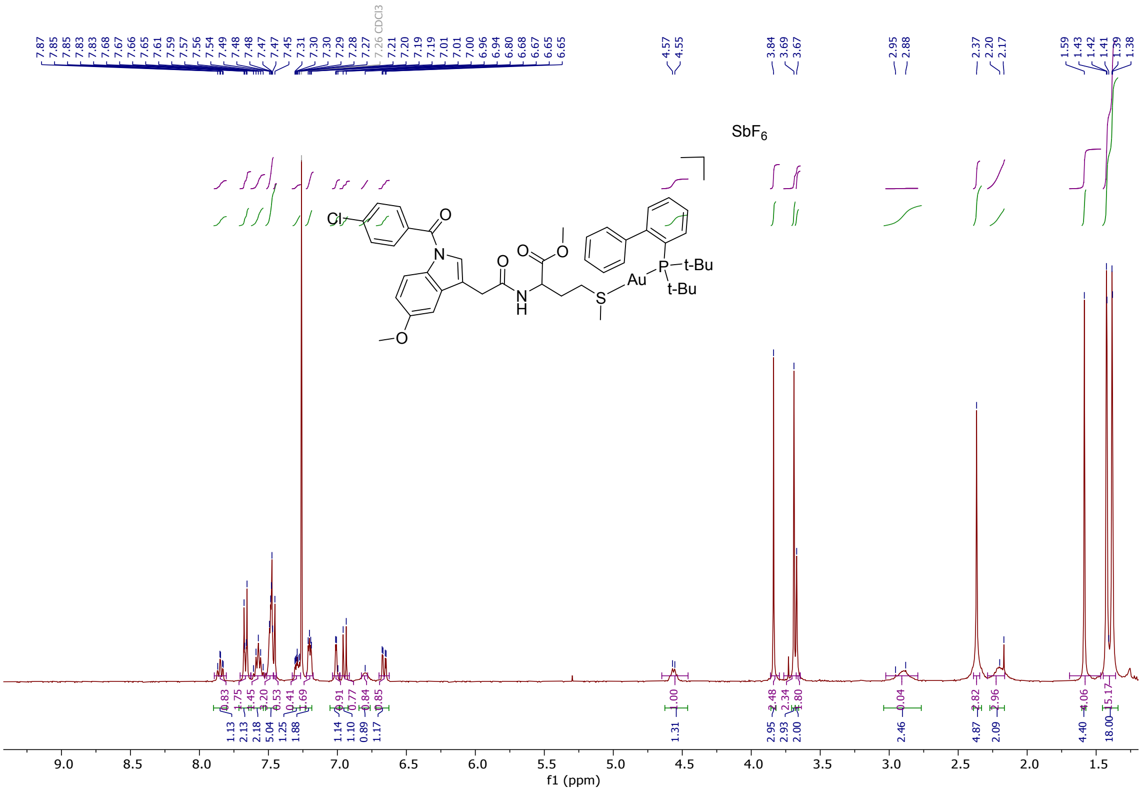

Figure S24. ^{1}H NMR spectrum (400 MHz, $CDCl_3$) of the complex 3b.

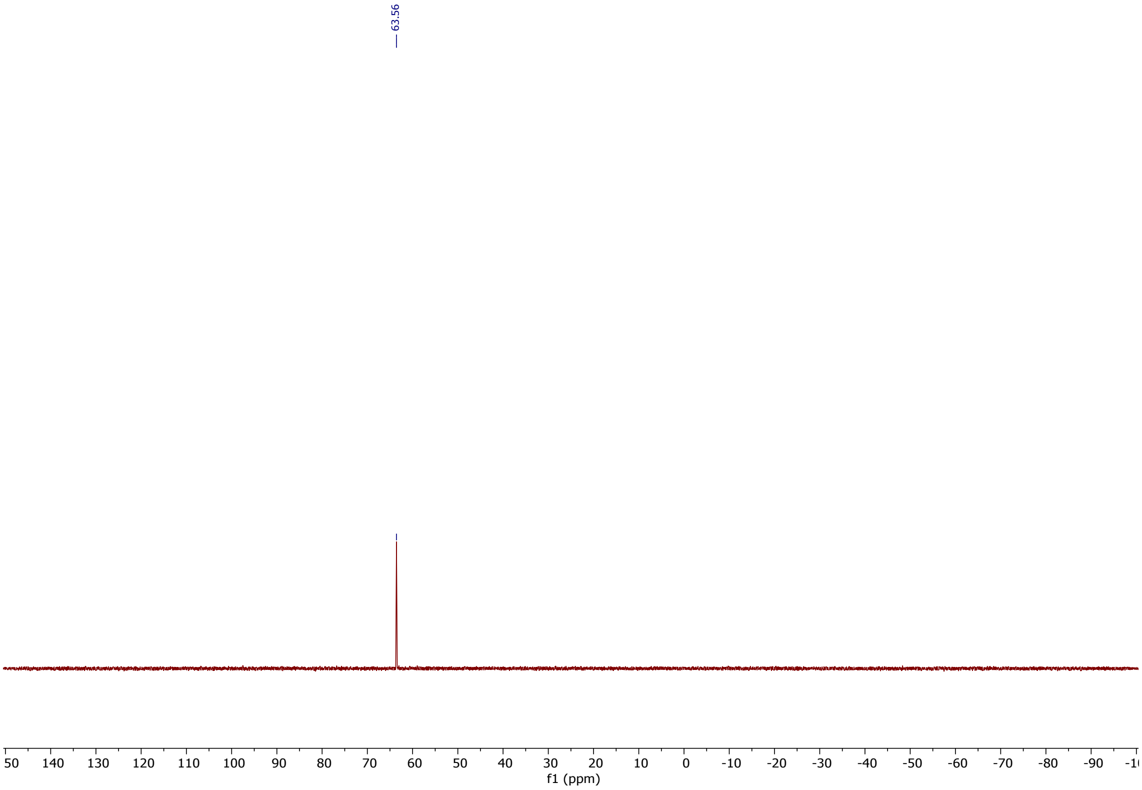

Figure 25. ^{31}P $\{^{1}H\}$ NMR spectrum (162 MHz, $CDCl_3$) of the complex 3b.

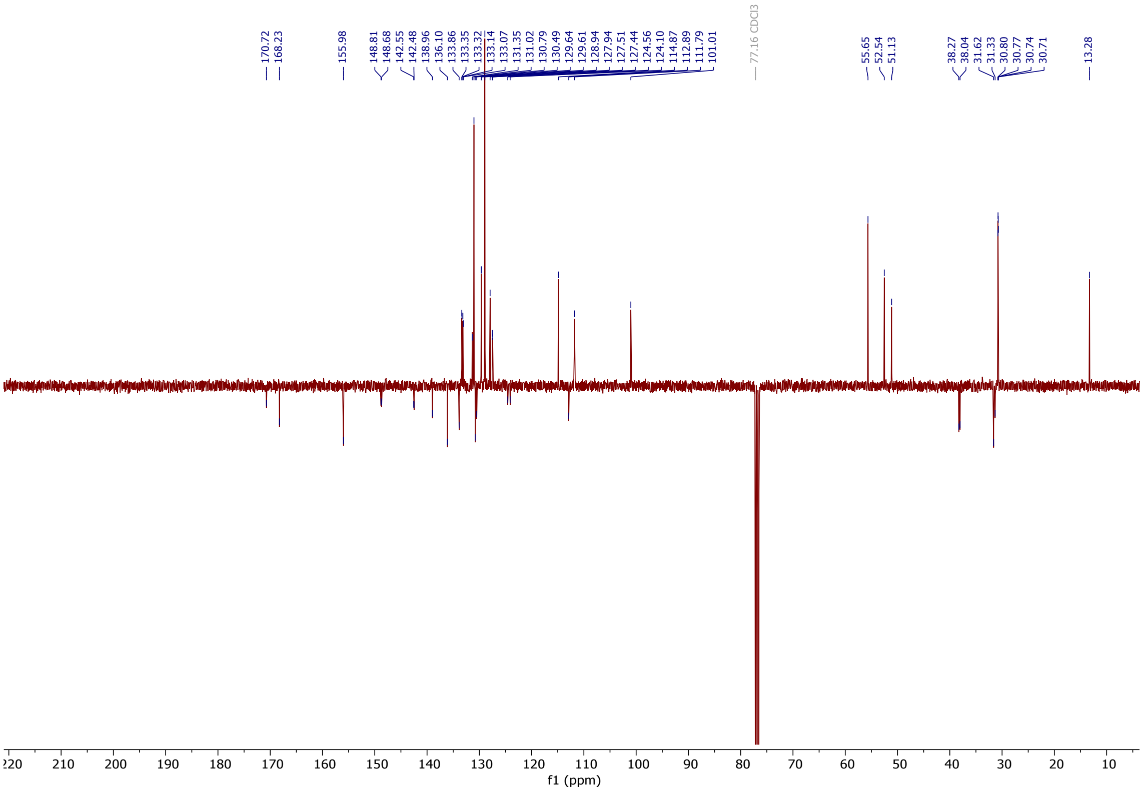

Figure S26. ^{13}C {^{1}H} NMR (101 MHz, $CDCl_3$) spectrum of compound 3b.

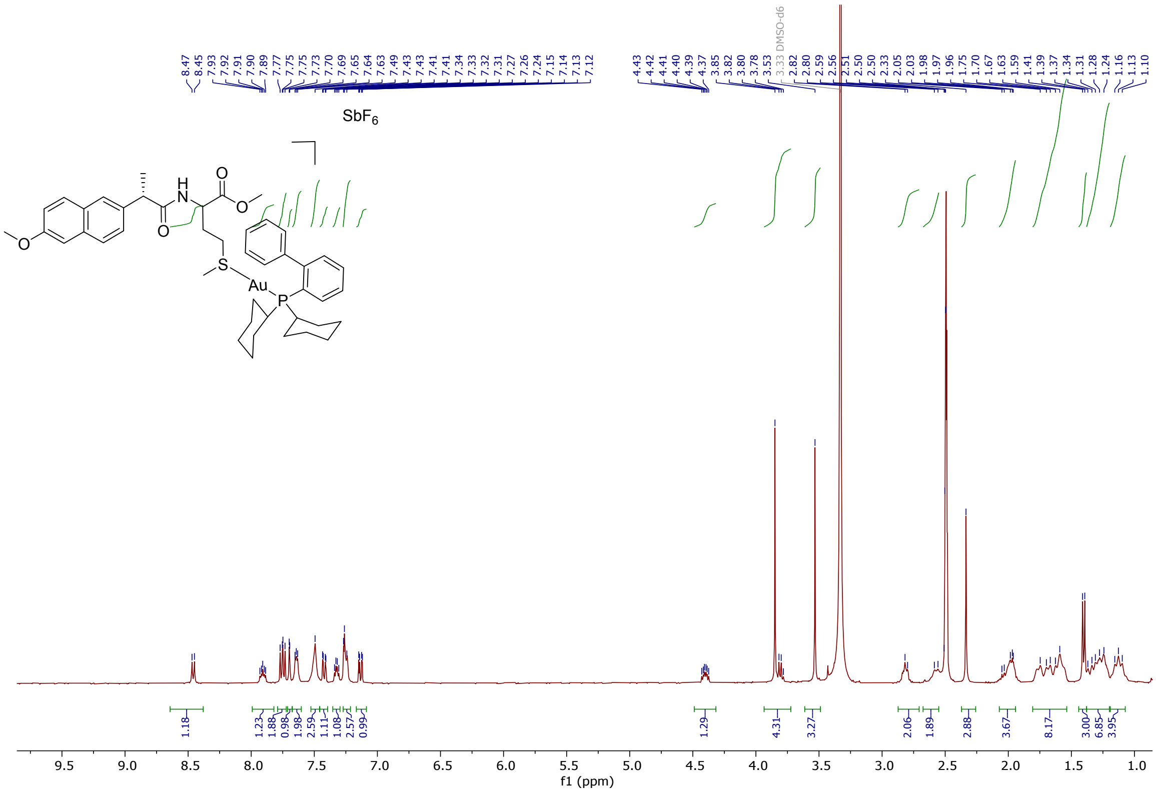

Figure S27. ^{13}C {^{1}H} NMR (101 MHz, DMSO-d_6) spectrum of compound 4a.

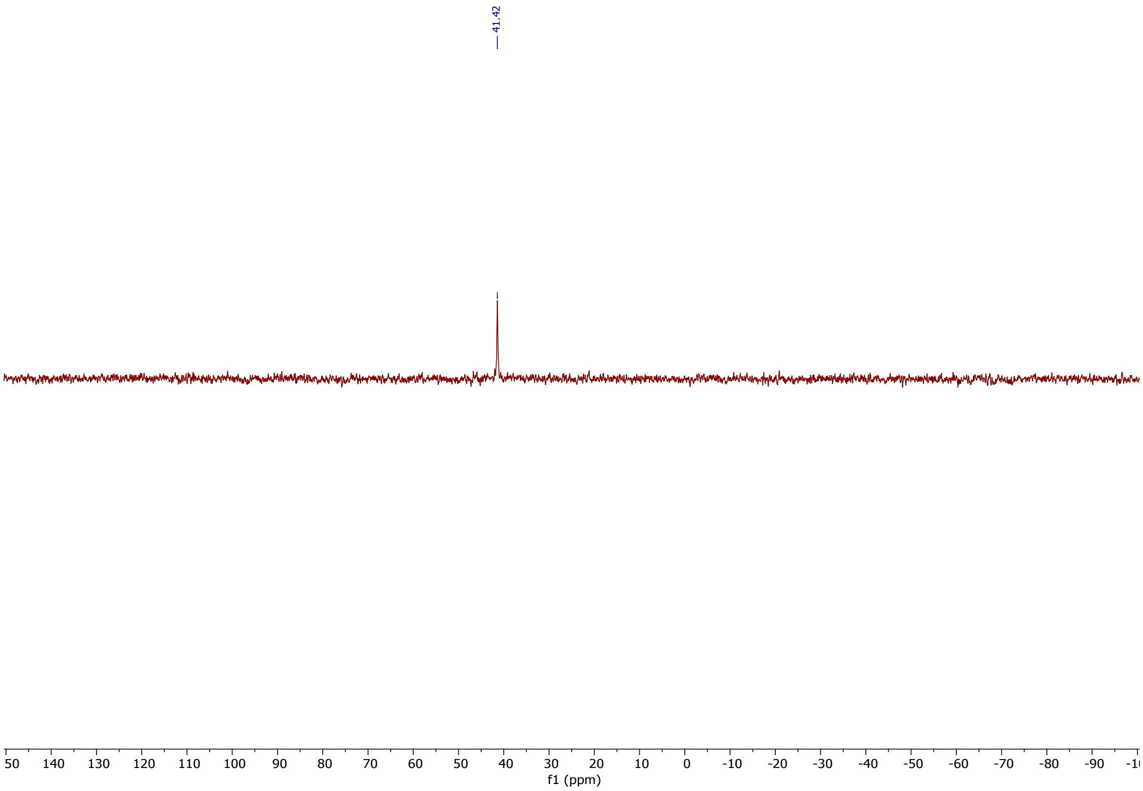

Figure S28. ^{31}P $\{^1H\}$ NMR spectrum (162 MHz, DMSO-d_6) of the complex 4a.

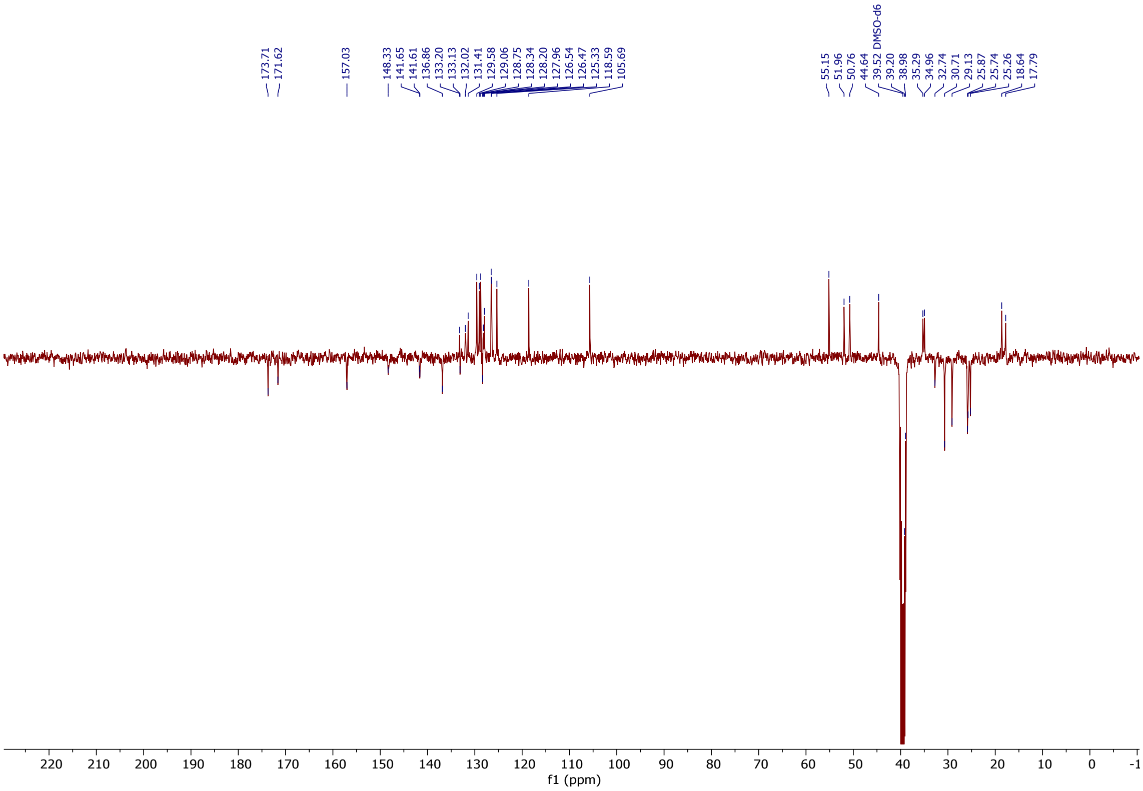

Figure S29. ^{13}C $\{^1H\}$ NMR (101 MHz, DMSO-d_6) spectrum of compound 4a.

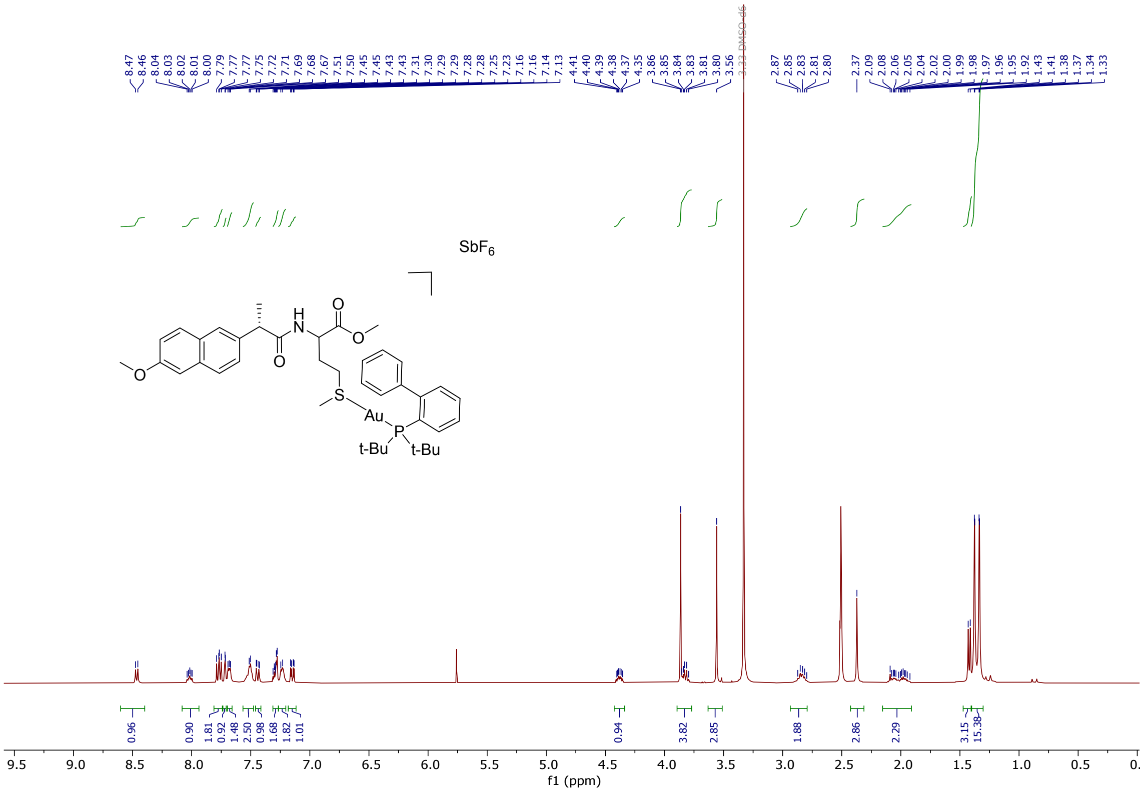

Figure S30. ^{13}C {^{1}H} NMR (101 MHz, DMSO-d$_6$) spectrum of compound 4b.

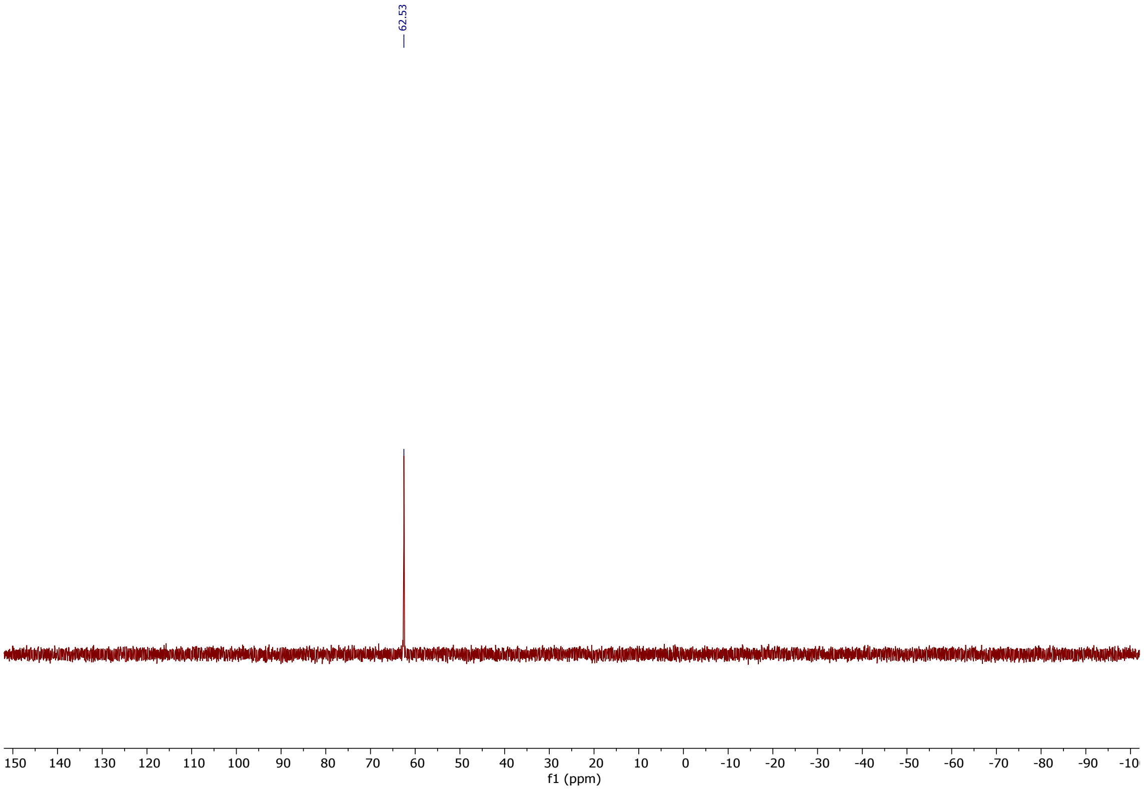

Figure S31. ^{31}P {^{1}H} NMR spectrum (162 MHz, DMSO-d$_6$) of the complex 4b.

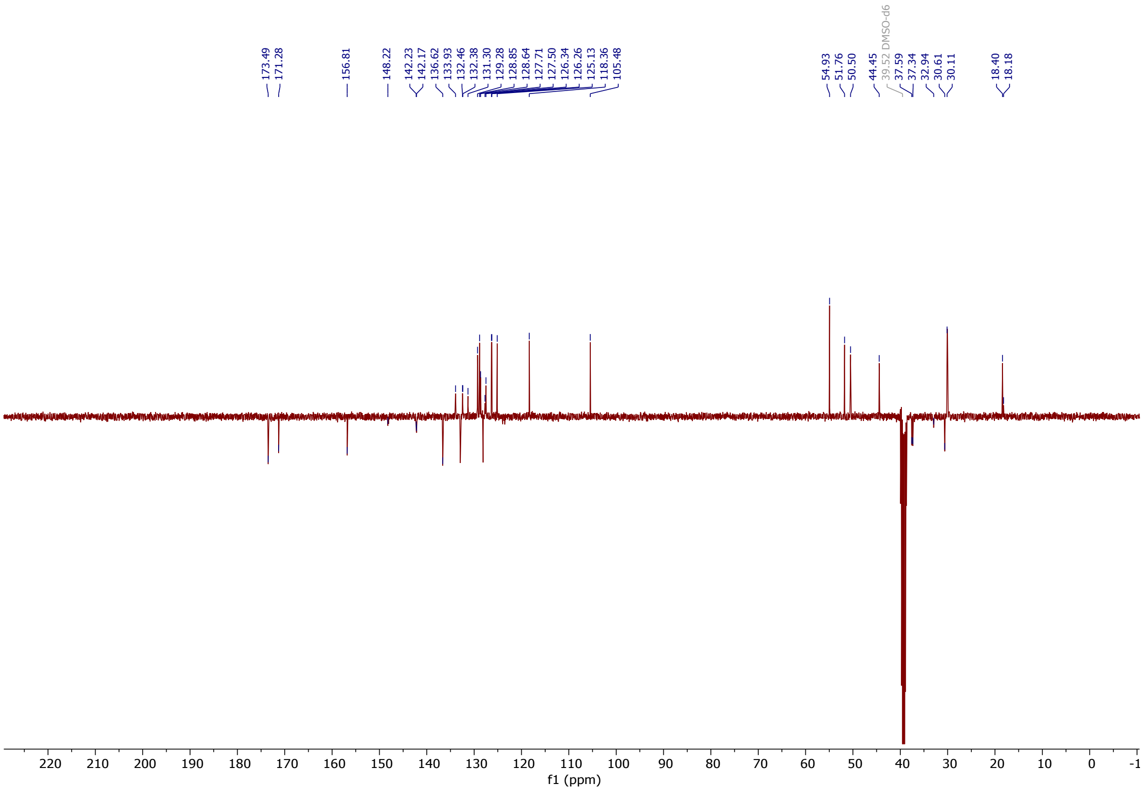

Figure S32. $^{13}C\ \{^{1}H\}$ NMR (101 MHz, DMSO-d_6) spectrum of compound 4a.

2 Mass spectra of compounds S33-S44.

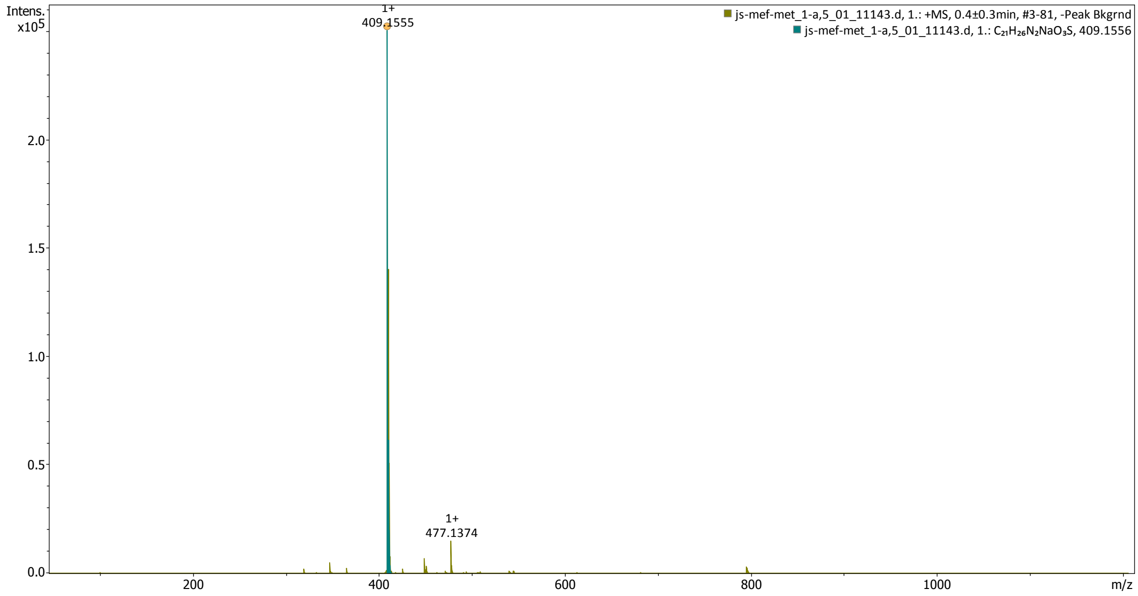

Figure S33. ESI-MS of L1 in Dicloromethane.

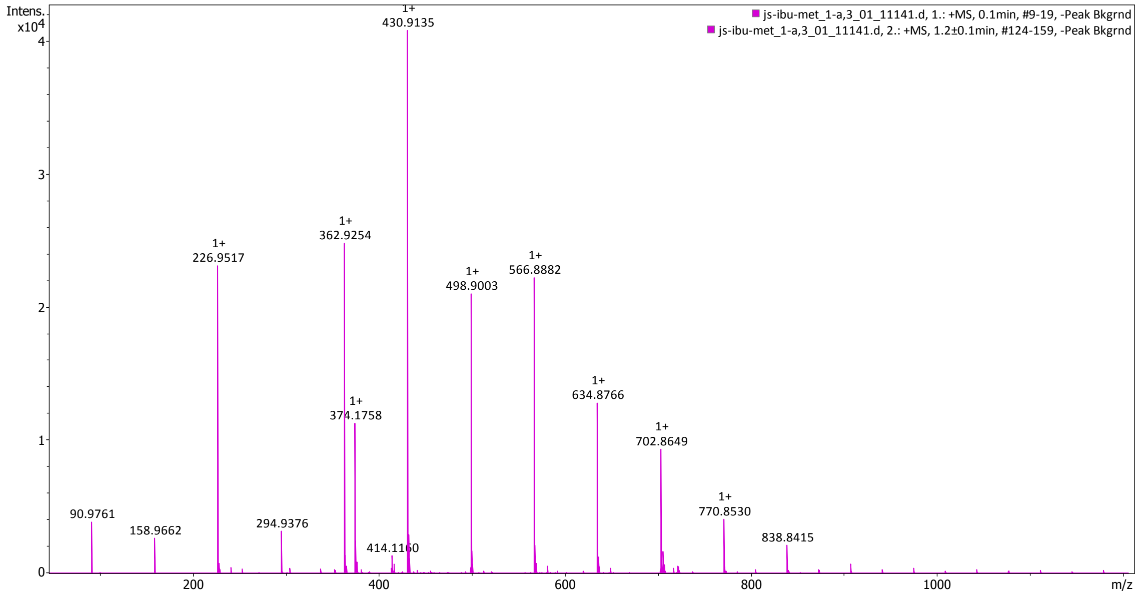

Figure S34. ESI-MS of L2 in Dicloromethane.

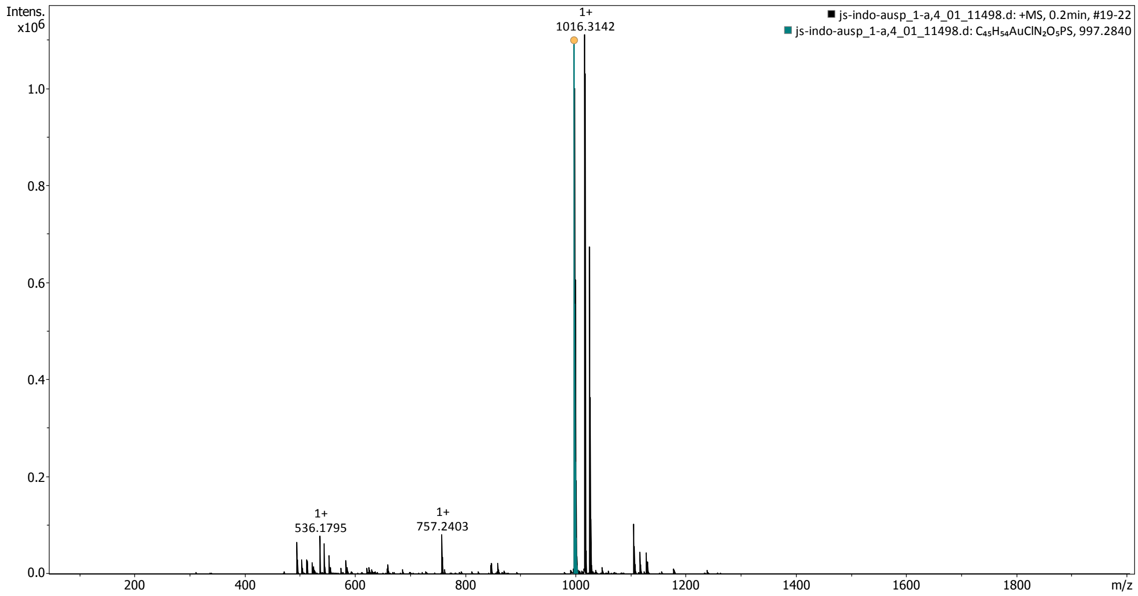

Figure S35. ESI-MS of L3 in Dicloromethane.

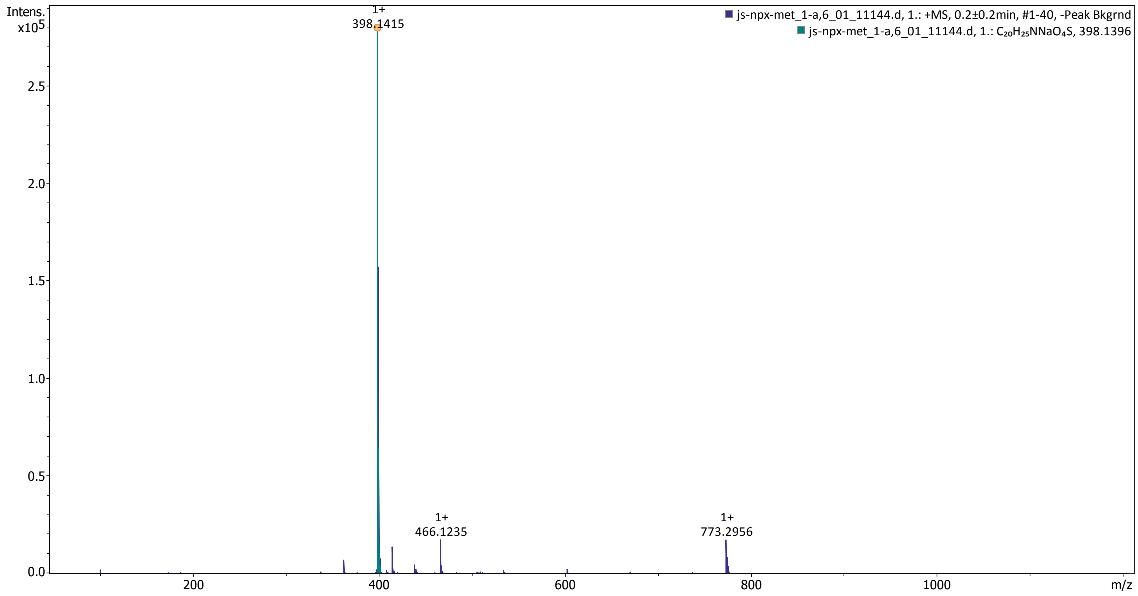

Figure S36. ESI-MS of L4 in Dicloromethane.

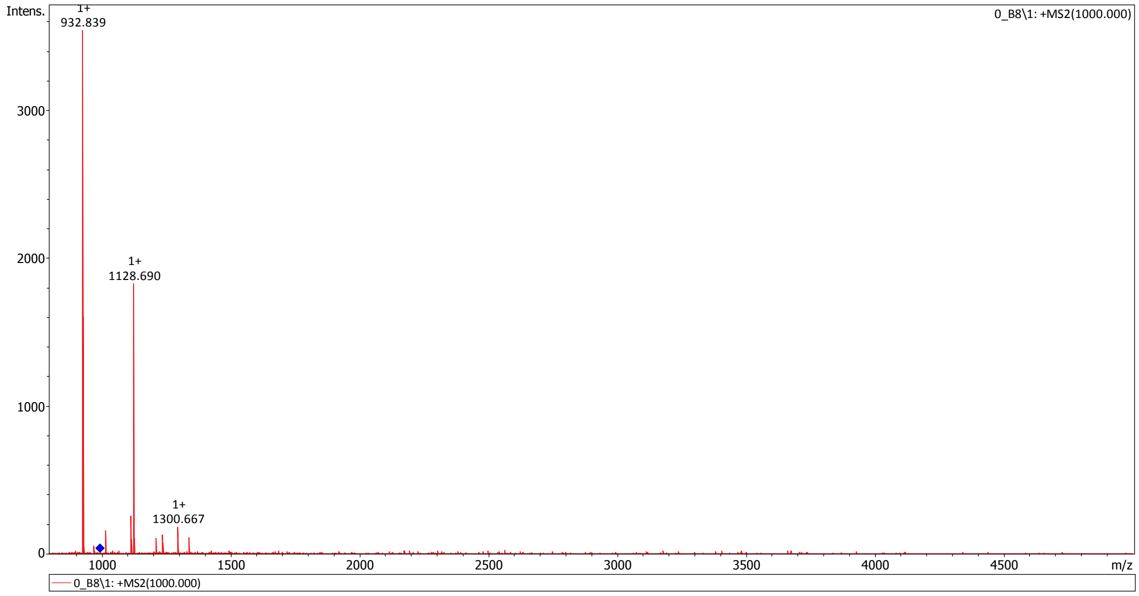

Figure S37. MALDI-MS of 1a in Dicloromethane.

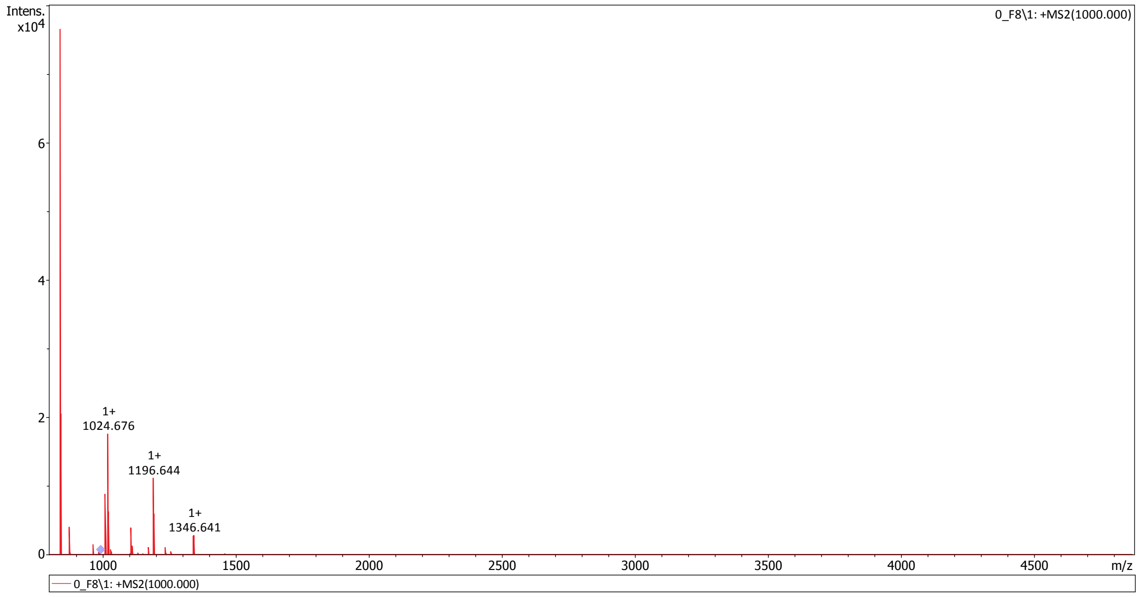

Figure S38. MALDI-MS of 1b in Dicloromethane.

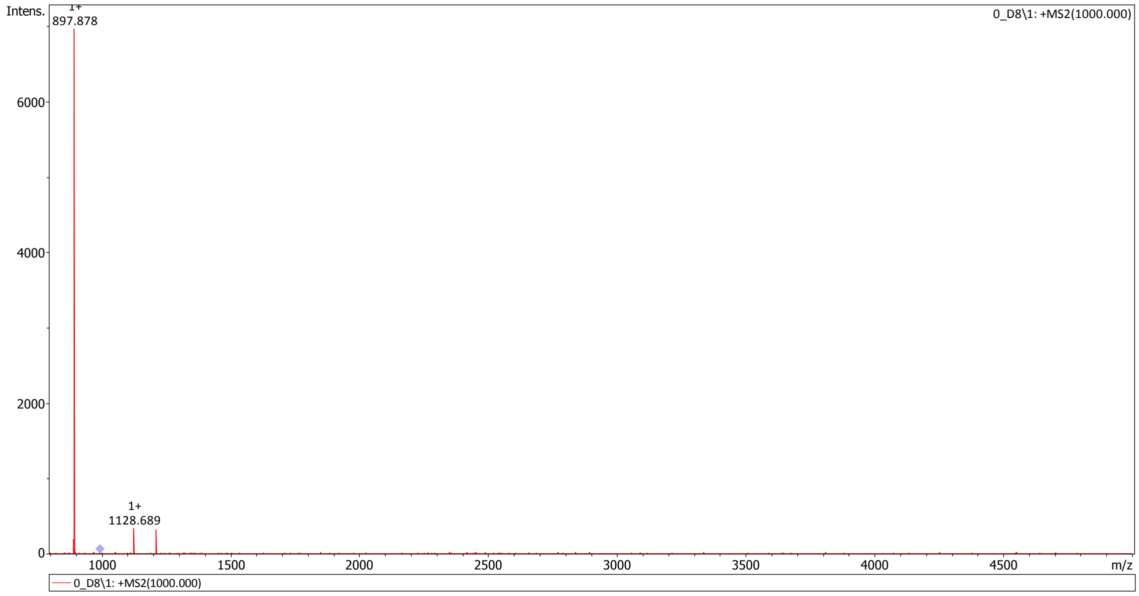

Figure S39. MALDI-MS of 2a in Dicloromethane.

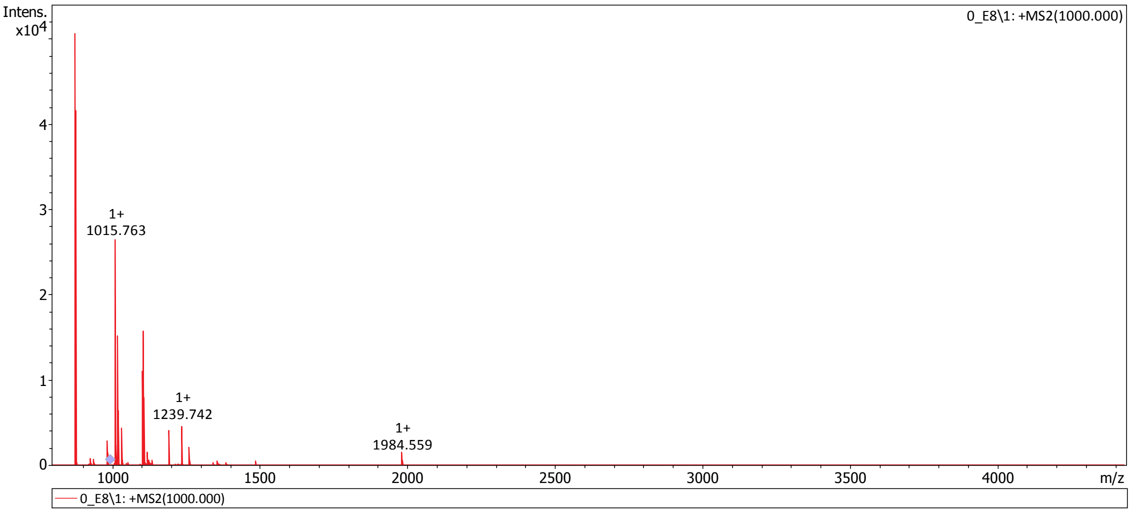

Figure S40. MALDI-MS of 2b in Dicloromethane.

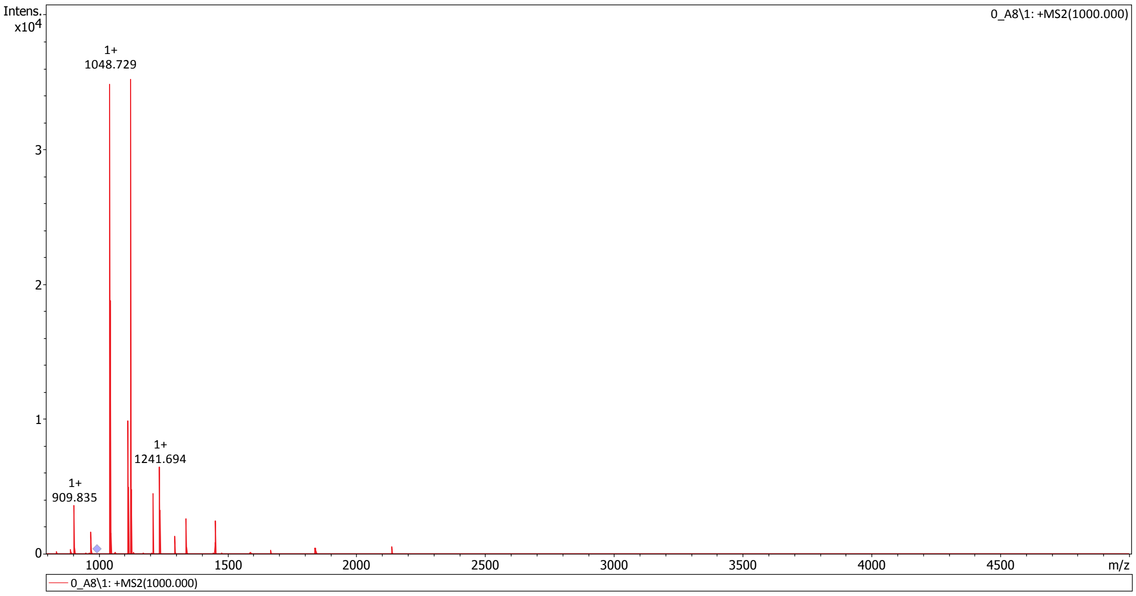

Figure S41. MALDI-MS of 3a in Dicloromethane.

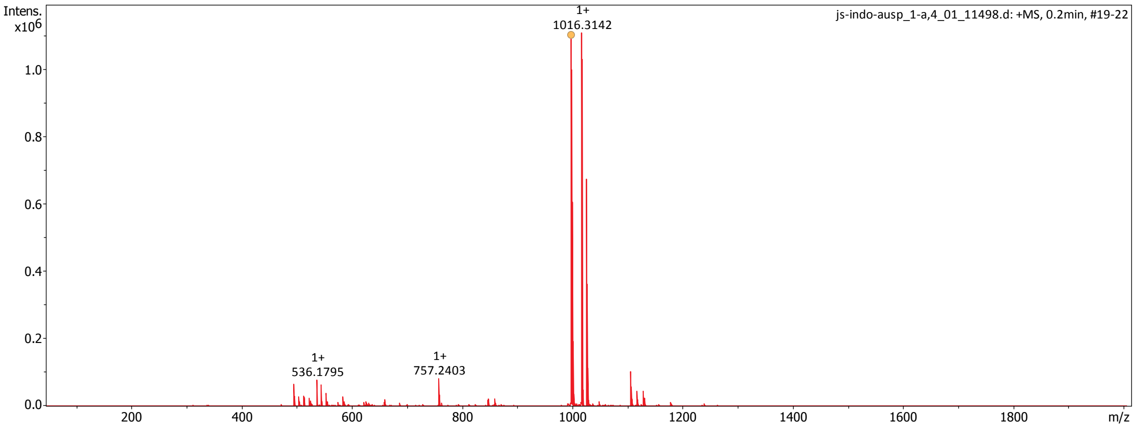

Figure S42. ESI-MS of 3b in Dicloromethane.

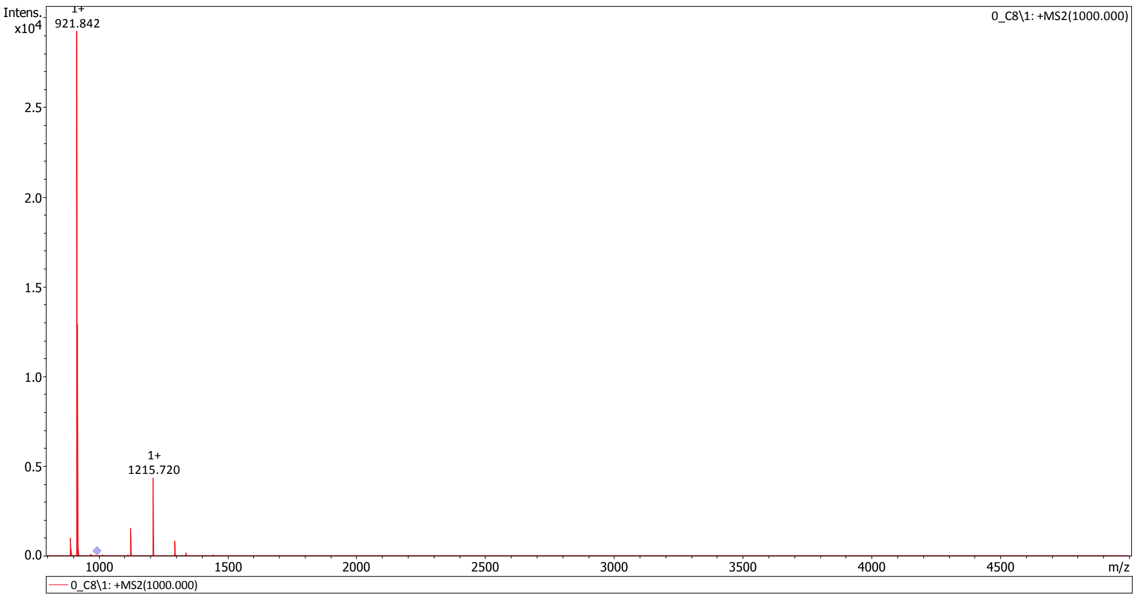

Figure S43. MALDI-MS of 4a in Dicloromethane.

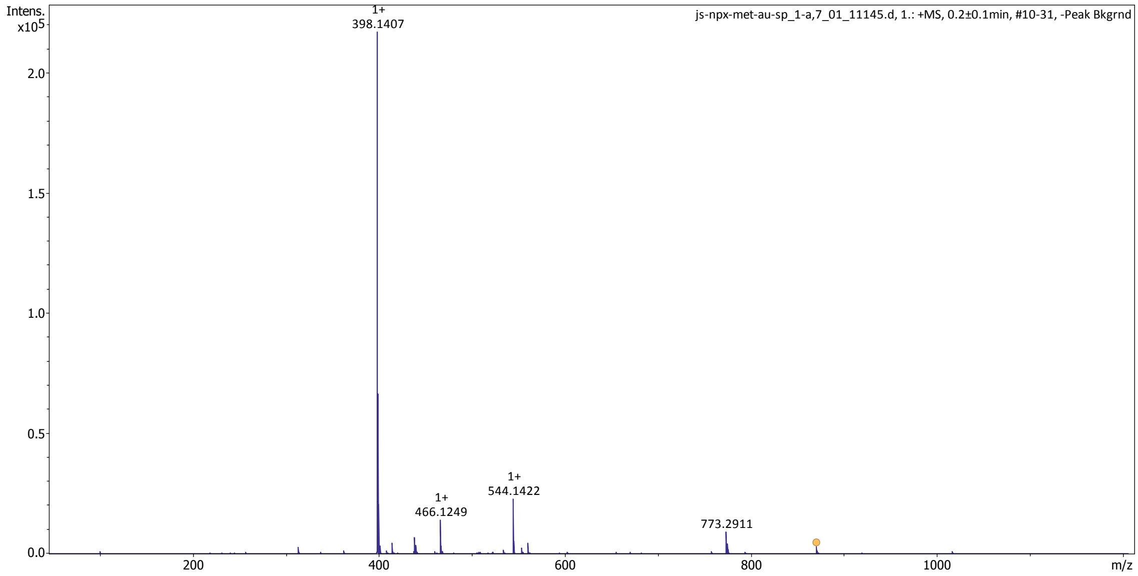

Figure S44. ESI-MS of 4a in Dicloromethane

5 **Stability assays S45-6.**

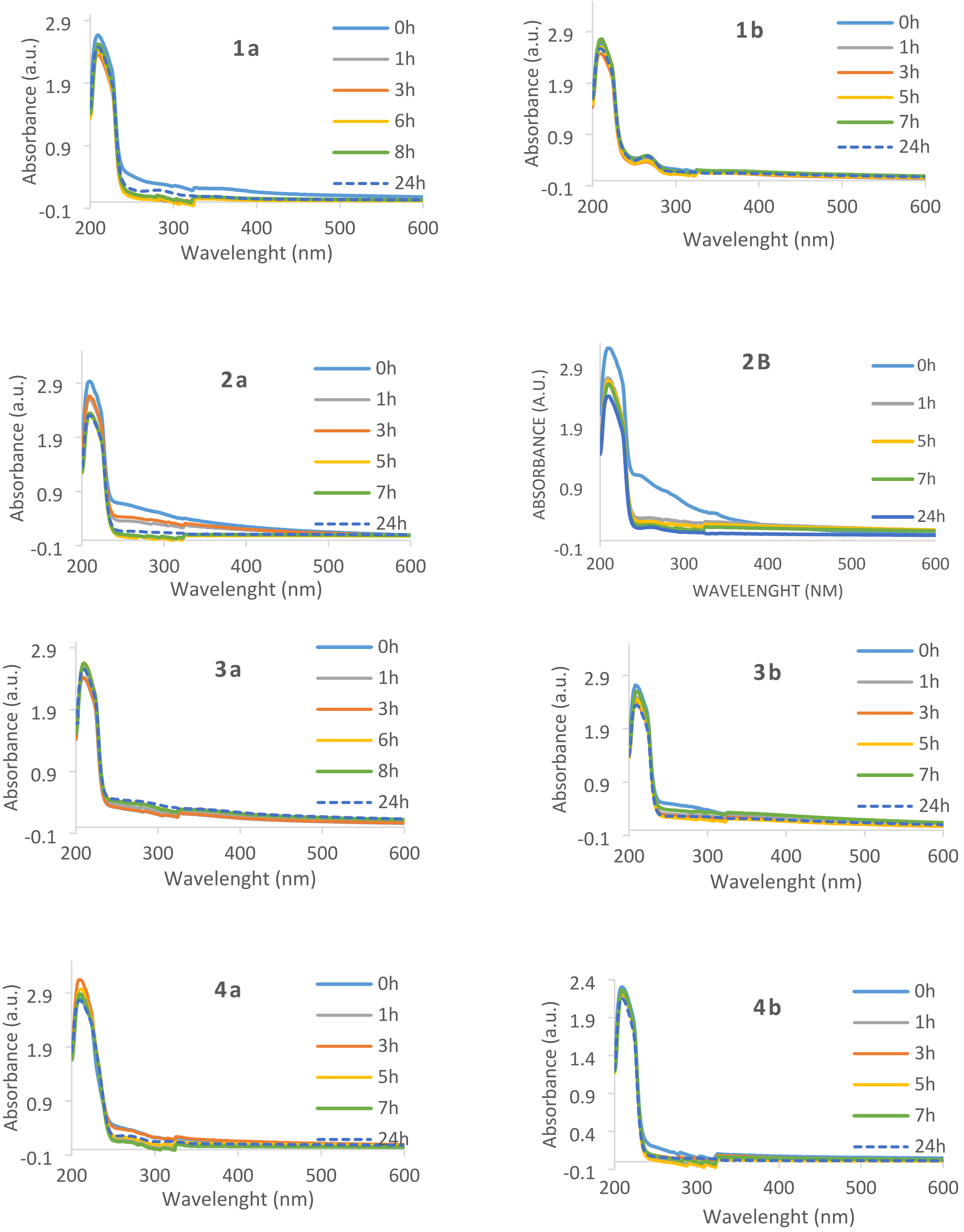

Figure S45 Figure S50. UV-Vis spectra of the gold(I) thioether complexes recorded at different times for 24 h to test their stability under physiological conditions. [complex] = 50 µM diluted in PBS at 37 ºC.

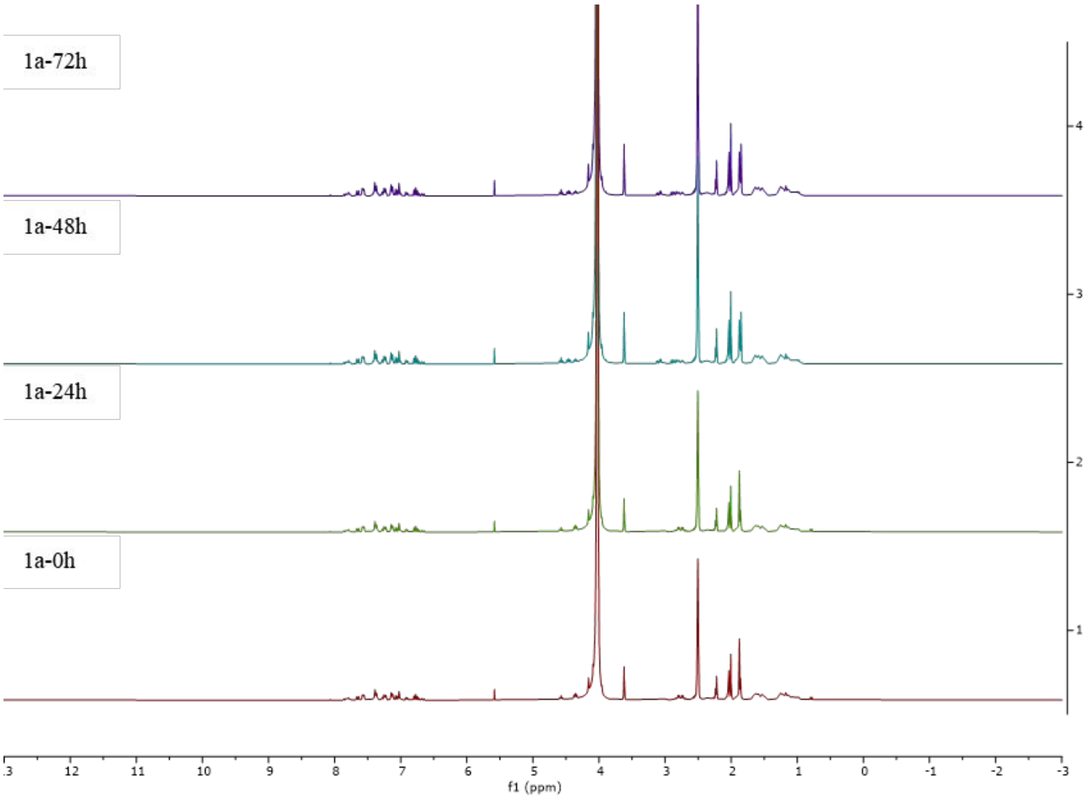

Figure S46. 1H NMR spectrum of 1a over 72 h in DMSO-d6/D_2O (8/2) with a NAC.

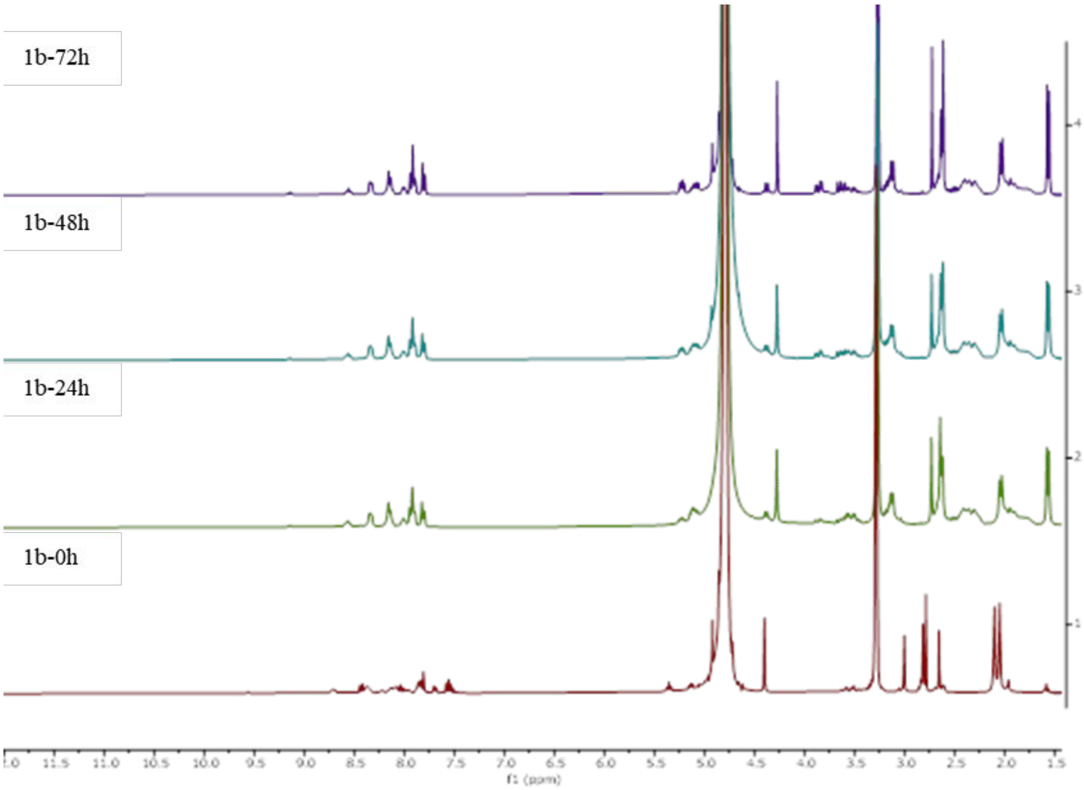

Figure S47. ^{1}H NMR spectrum of 1b over in 72 h DMSO-d6/D_2O (8/2) with a NAC.

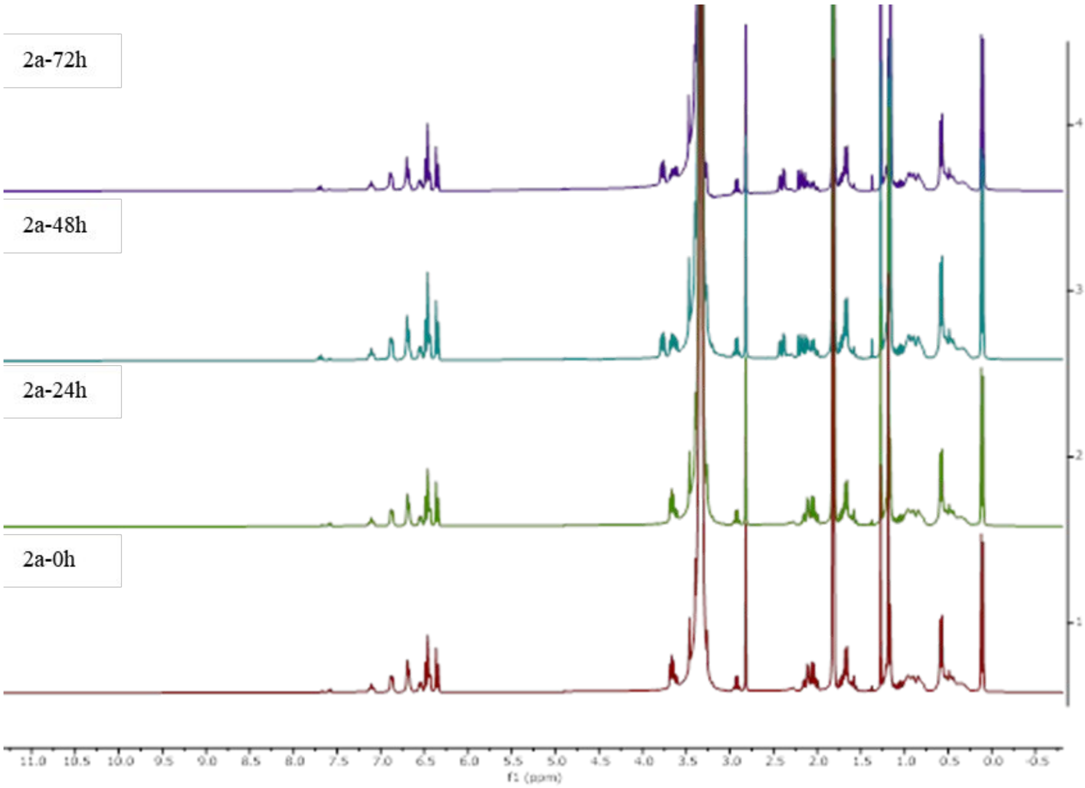

Figure S48. ^{1}H NMR spectrum of 2a in over 72 h in DMSO-d6/D_2O (8/2) with a NAC.

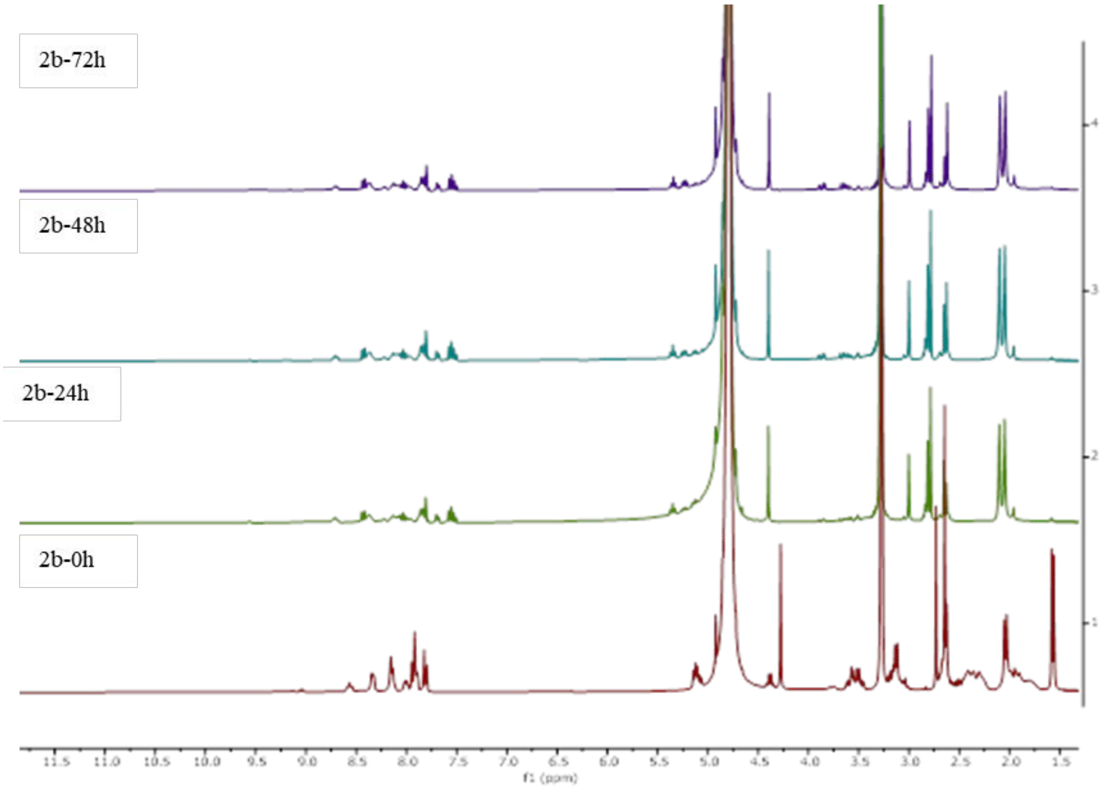

Figure S49. ^{1}H NMR spectrum of 2b over 72h in DMSO-d6/D_2O (8/2) with a NAC.

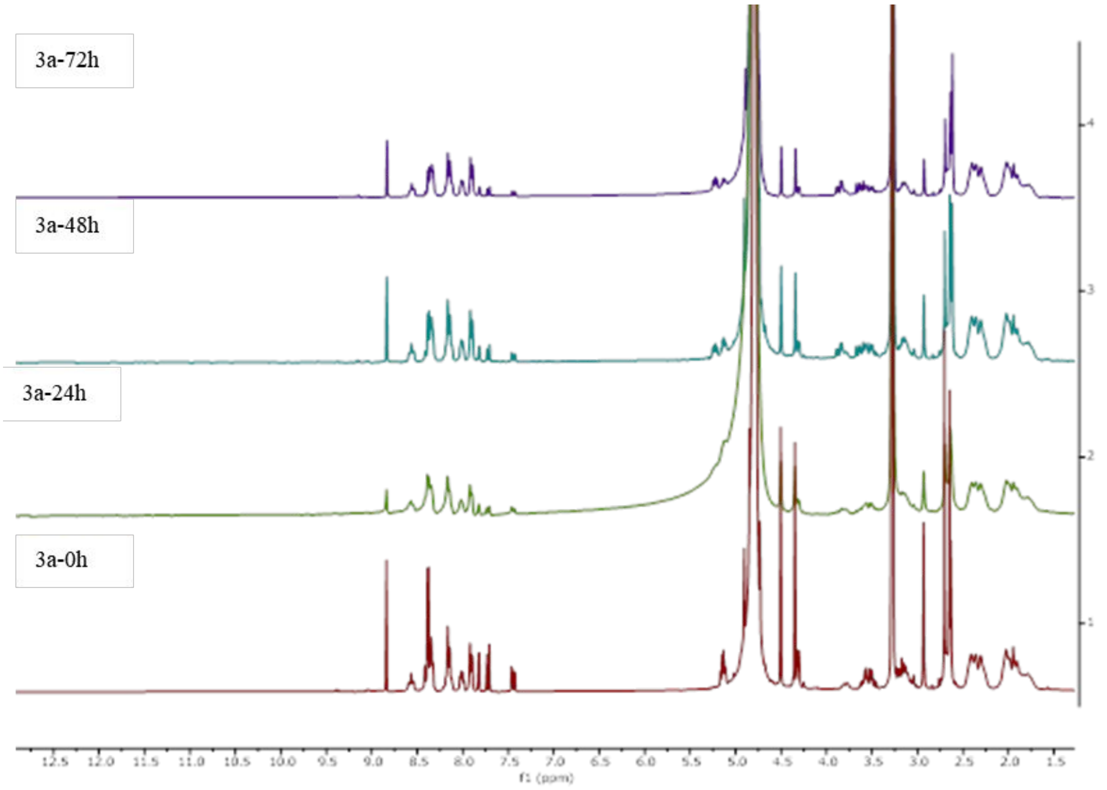

Figure S50. ^{1}H NMR spectrum 3a over 72h in DMSO-d6/D_2O (8/2) with a NAC.

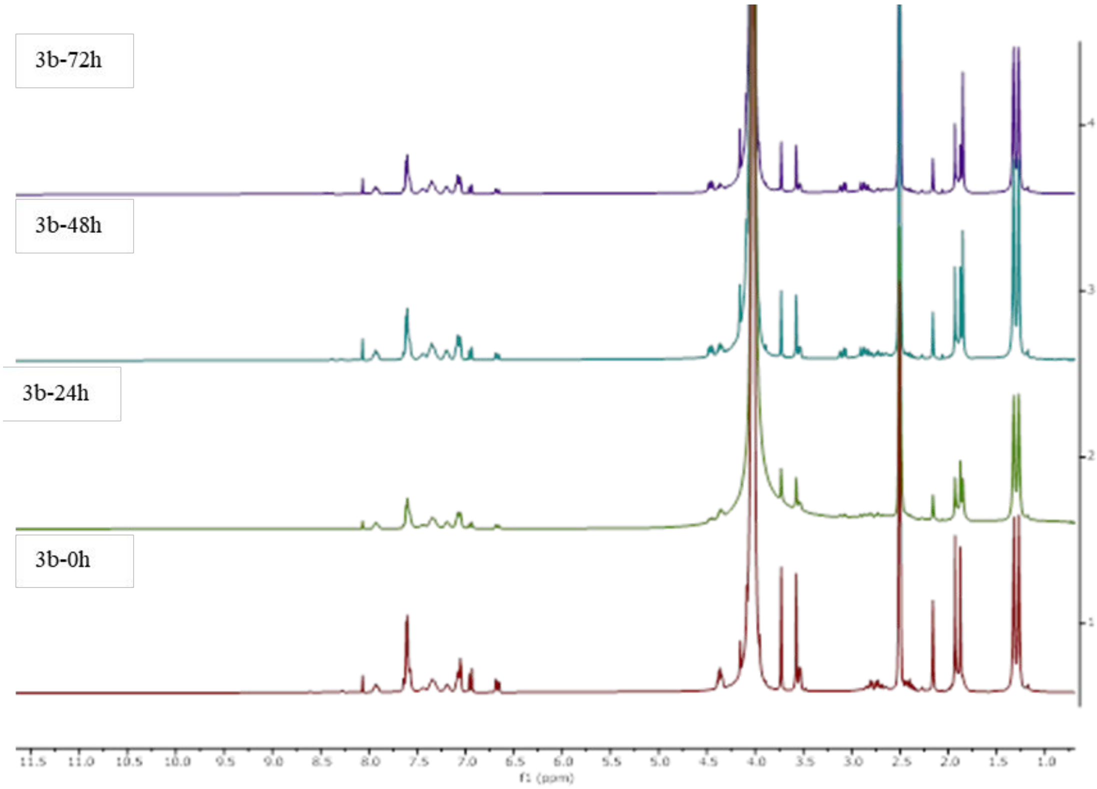

Figure S51. ^{1}H NMR spectrum of 3b over 72h in DMSO-d6/D_2O (8/2) with a NAC.

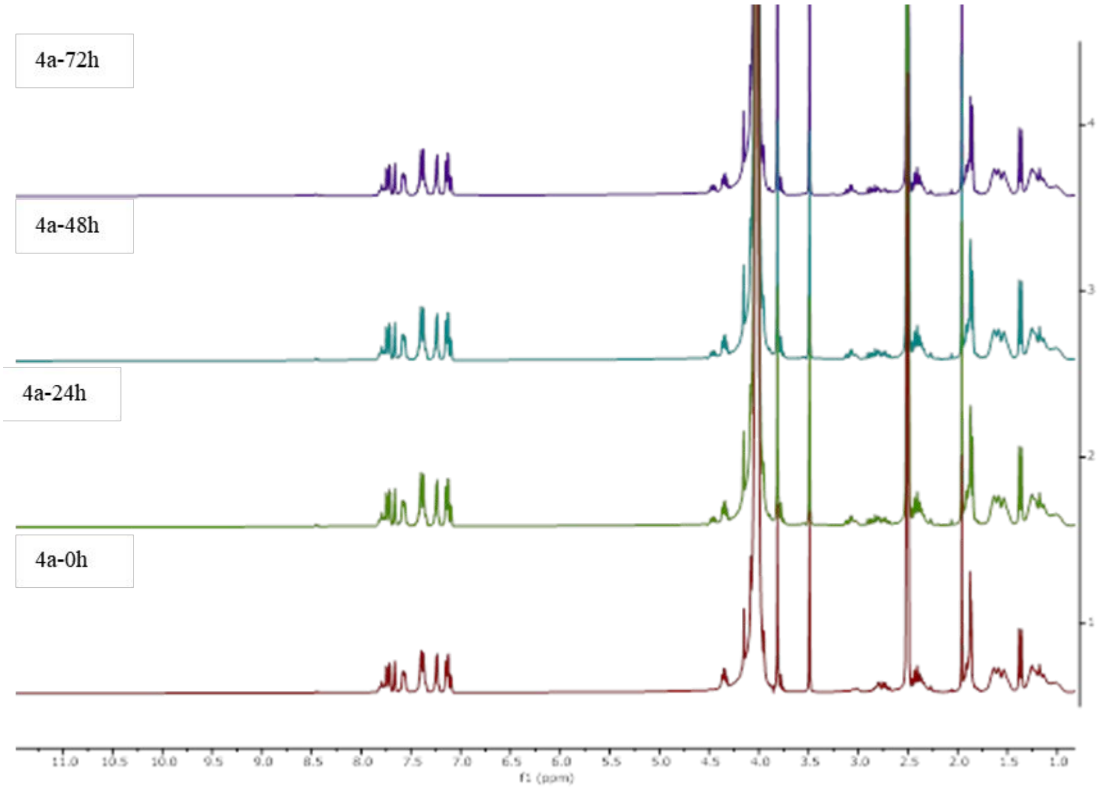

Figure S52. ^{1}H NMR spectrum of 4a over 72h in MSO-d6/D_2O (8/2) with a NAC.

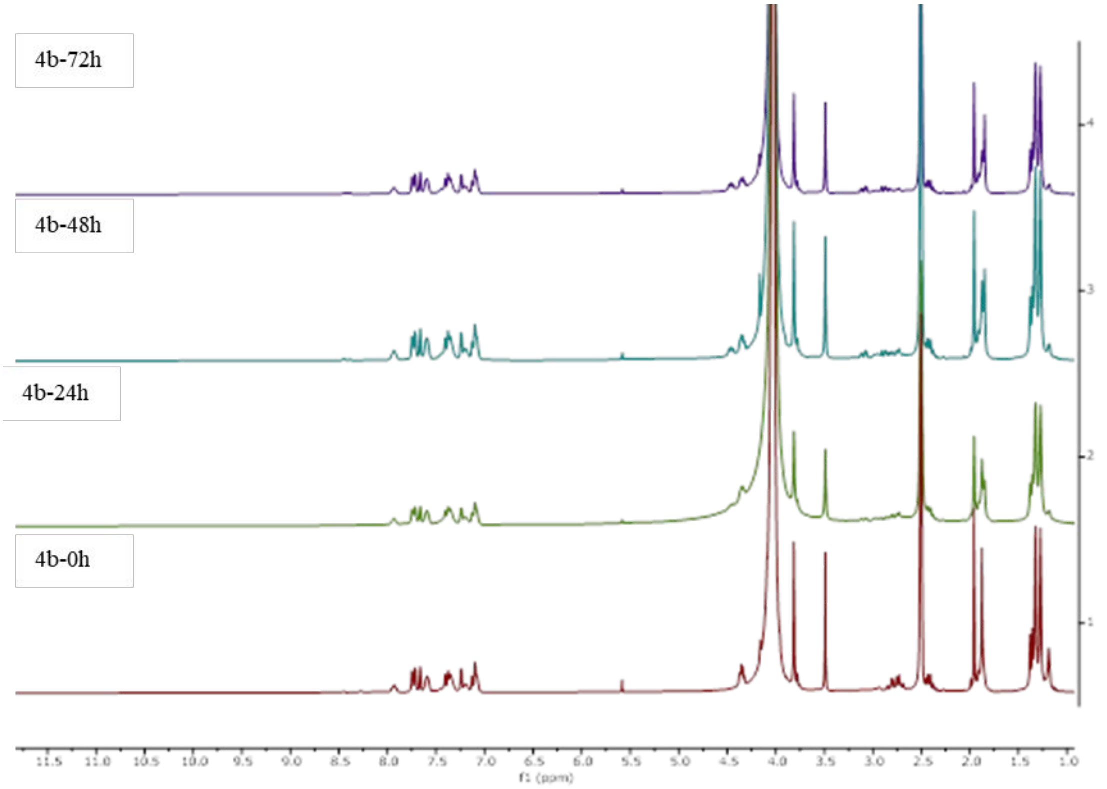

Figure S53. ^{1}H NMR spectrum of 4b over 72h in DMSO-d6/D_2O (8/2) with a NAC.

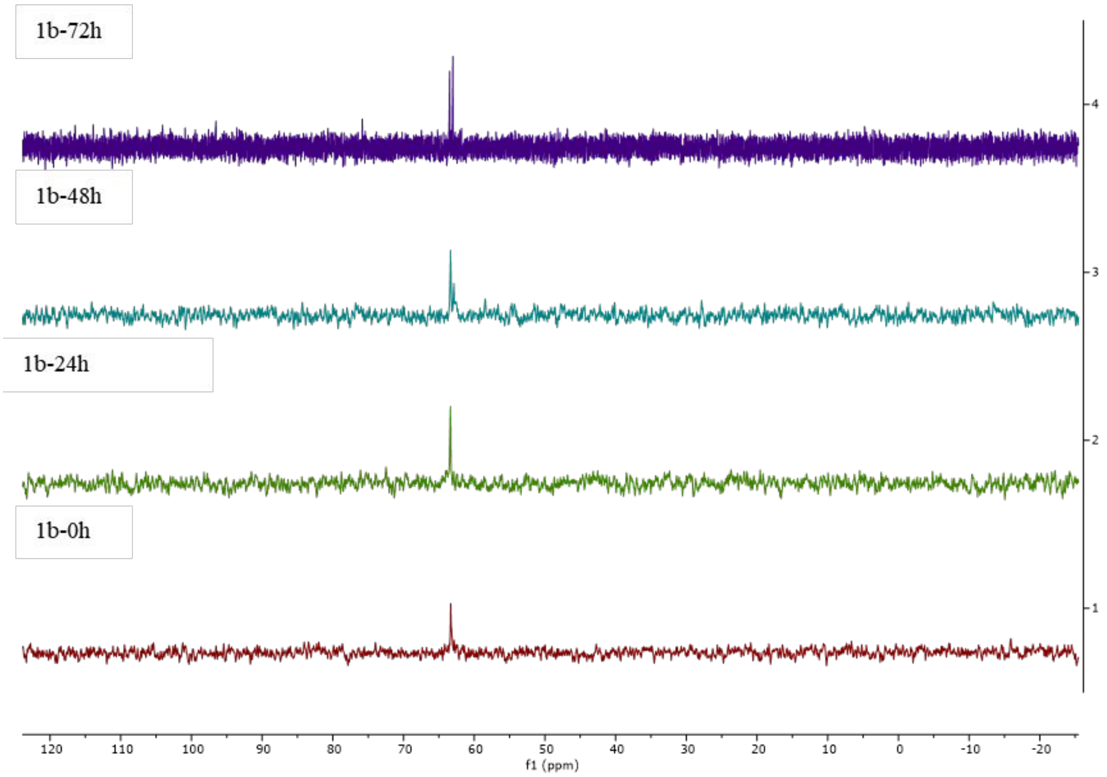

Figure S54.^{31}P {^{1}H} NMR spectrum of 1b over 72h in DMSO-d6/D_2O (8/2) with a NAC.

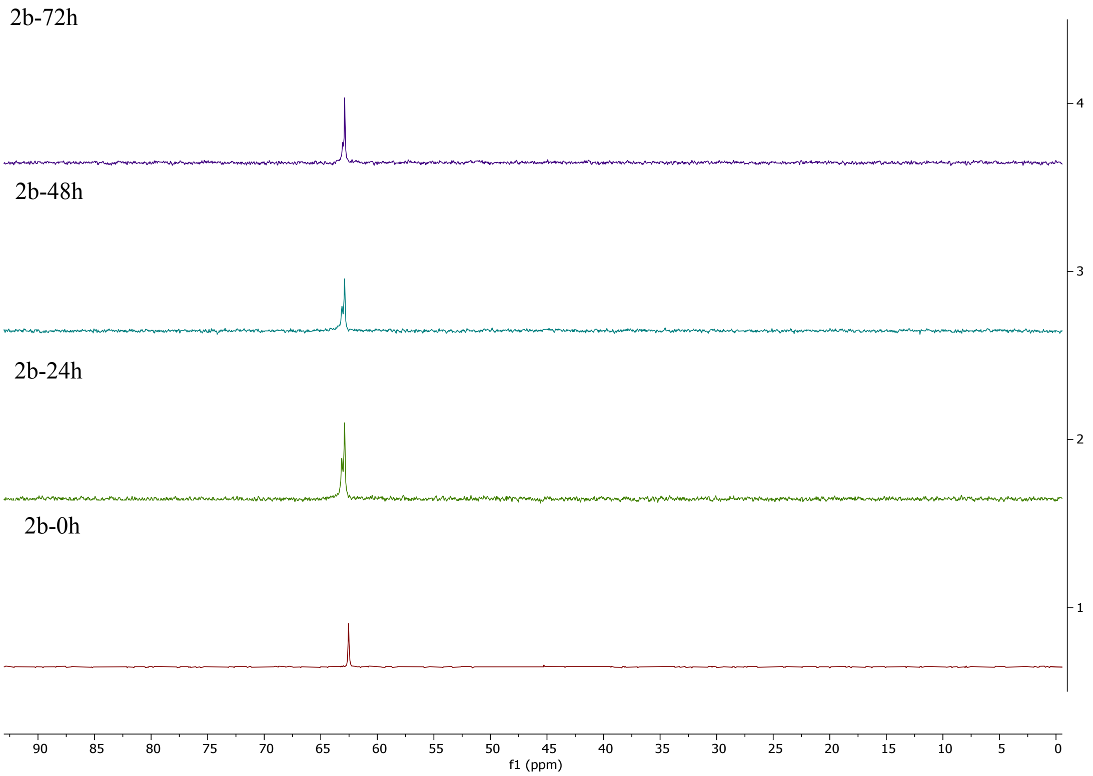

Figure S55.^{31}P {^{1}H} NMR spectrum of 2b over 72h in DMSO-d6/D_2O (8/2) with a NAC.

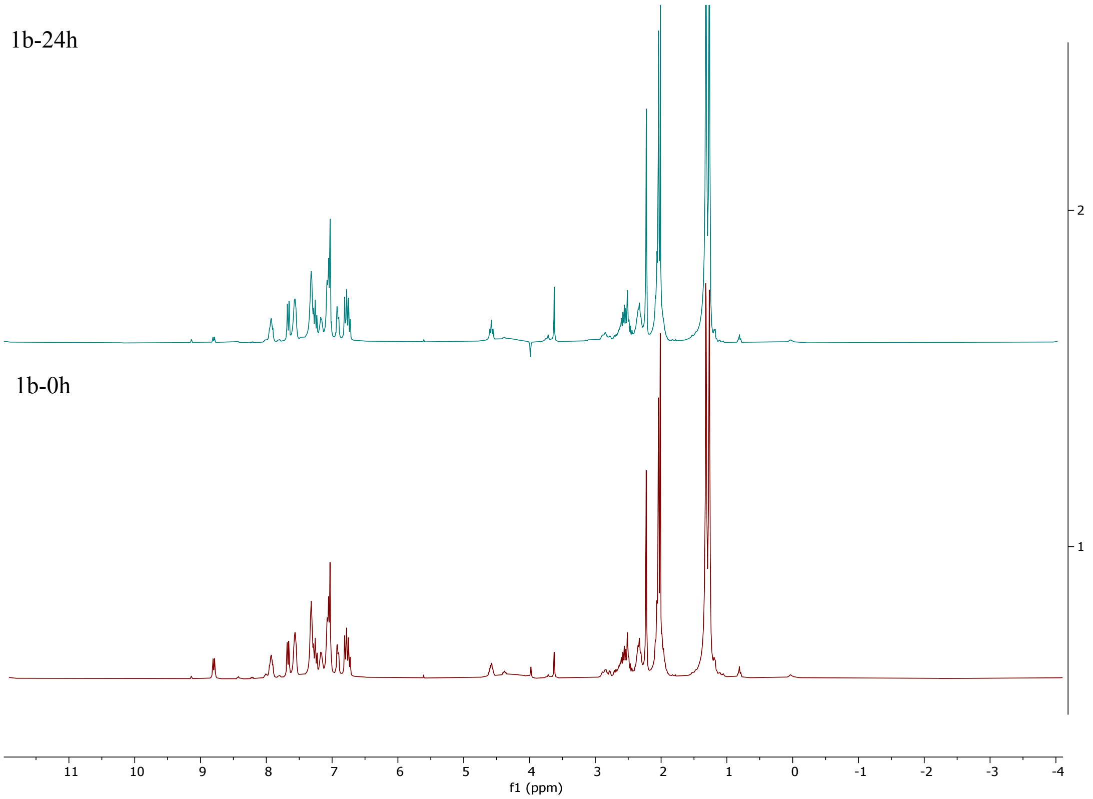

Figure S56. ^{1}H NMR NMR spectrum of 1b over 24h in DMSO-d6/D_2O (8/2) with a GSH.

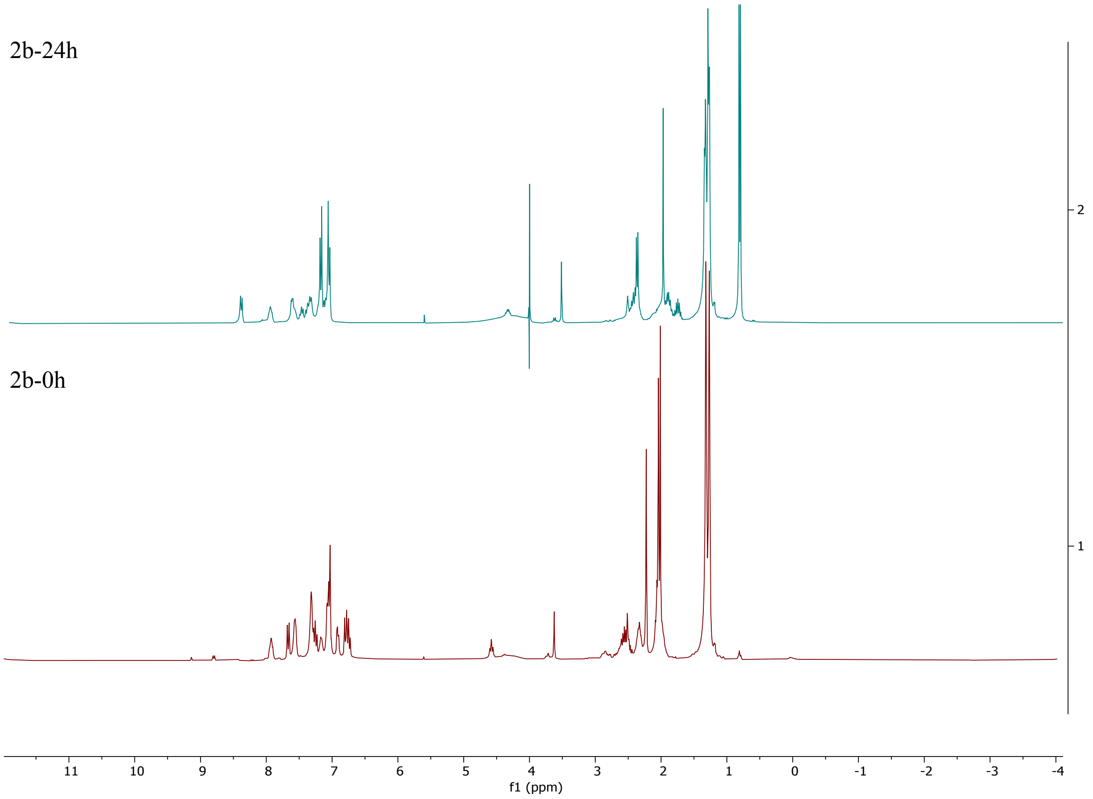

Figure S57. ^{1}H NMR NMR spectrum of 1b over 24h in DMSO-d6/D_2O (8/2) with a GSH.

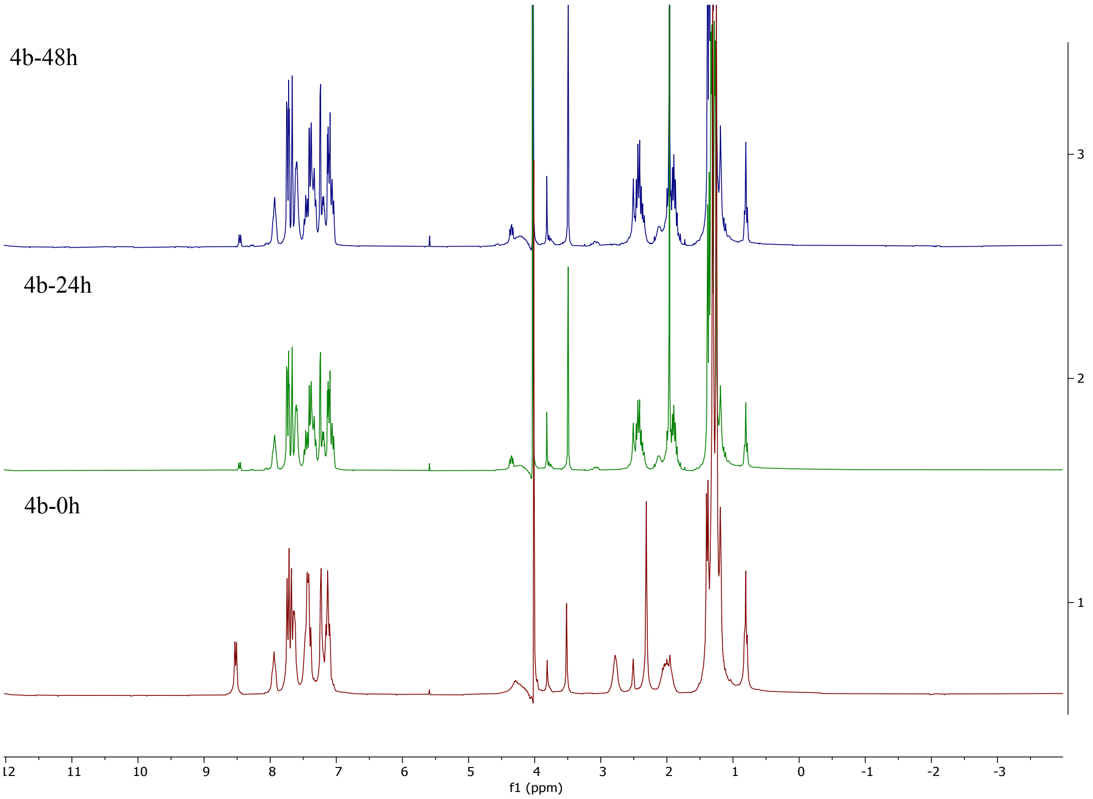

Figure S58. ^{1}H NMR NMR spectrum of 2b over 48h in DMSO-d6/D_2O (8/2) with a GSH.

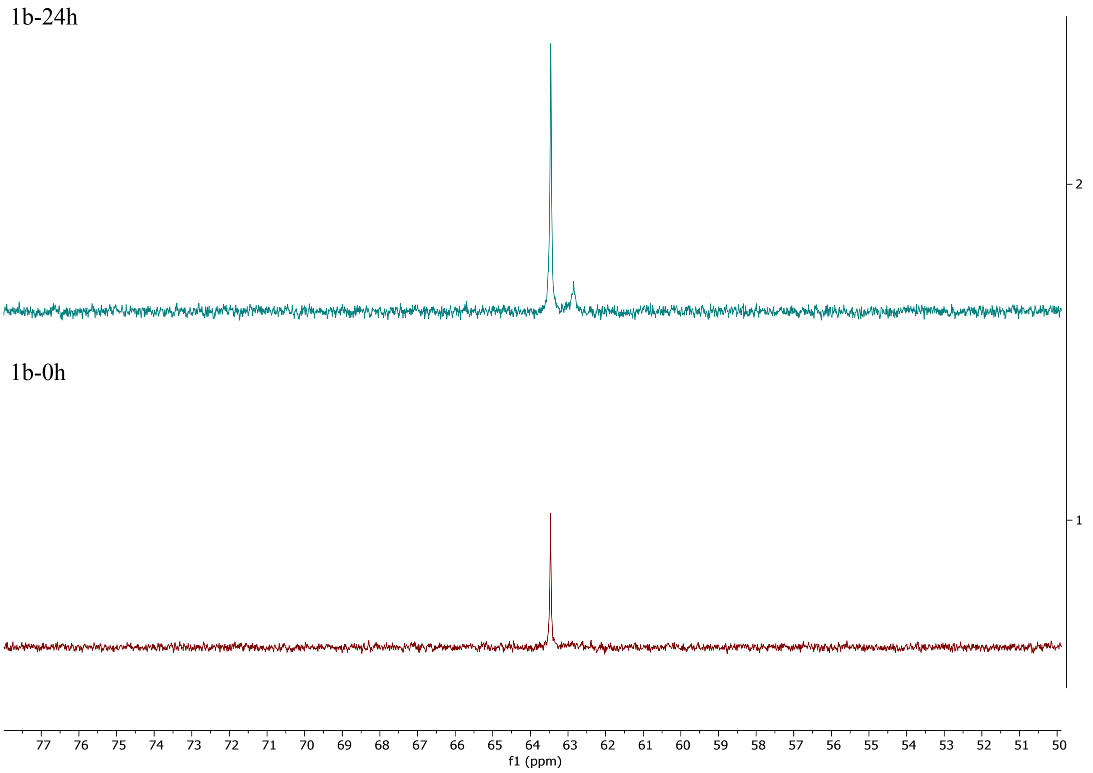

Figure S59. ^{31}P {^{1}H} NMR spectrum of 1b over 24h in DMSO-d6/D_2O (8/2) with a GSH.

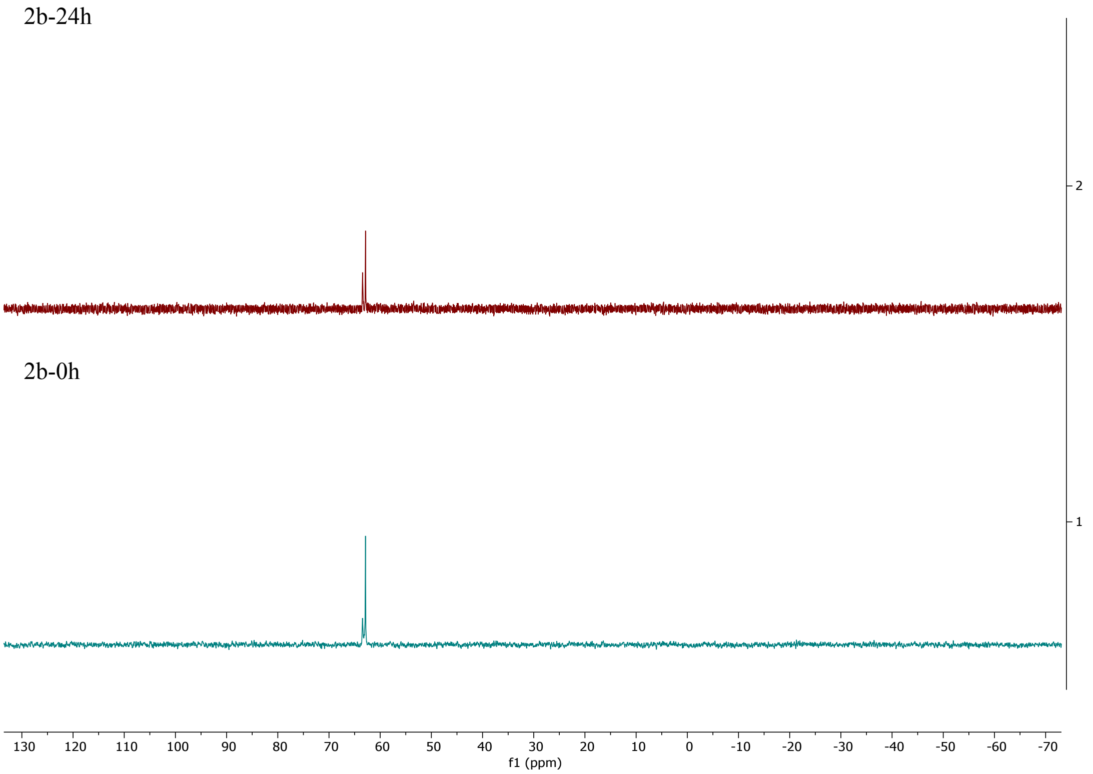

Figure S60. ^{31}P {^{1}H} NMR spectrum of 2b over 24h in DMSO-d6/D_2O (8/2) with a GSH.

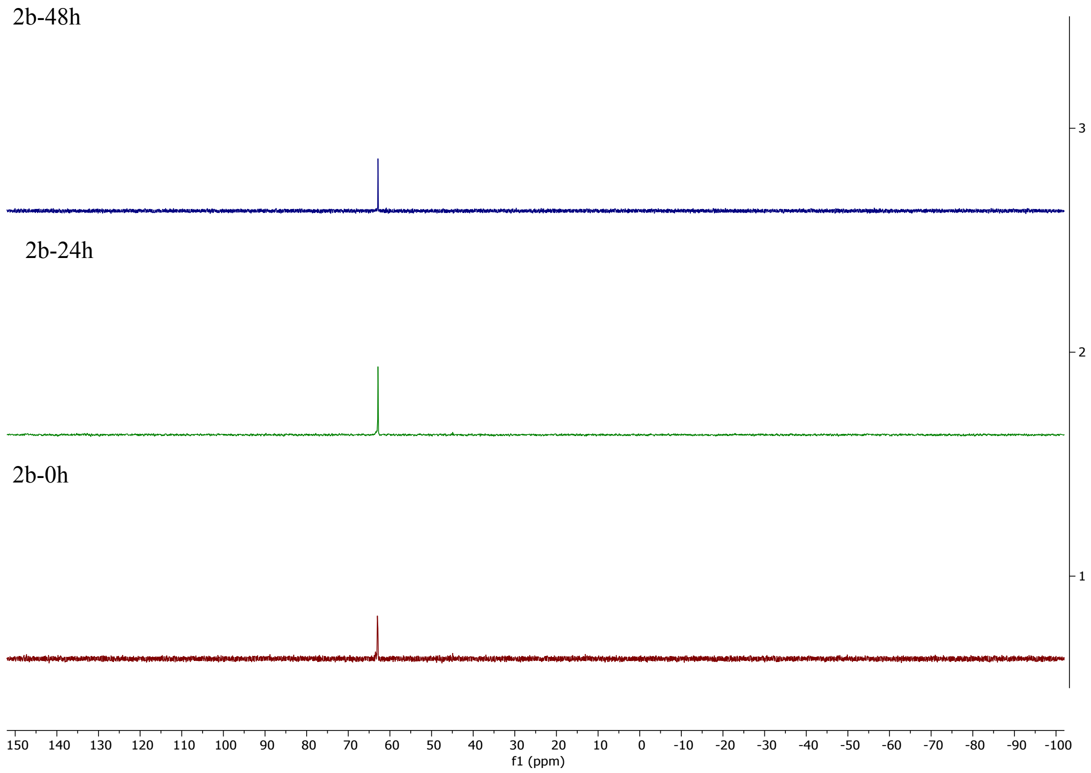

Figure S61. $^{31}P\ \{^1H\}$ NMR spectrum of 4b over 48h in DMSO-d6/D_2O (8/2) with a GSH.

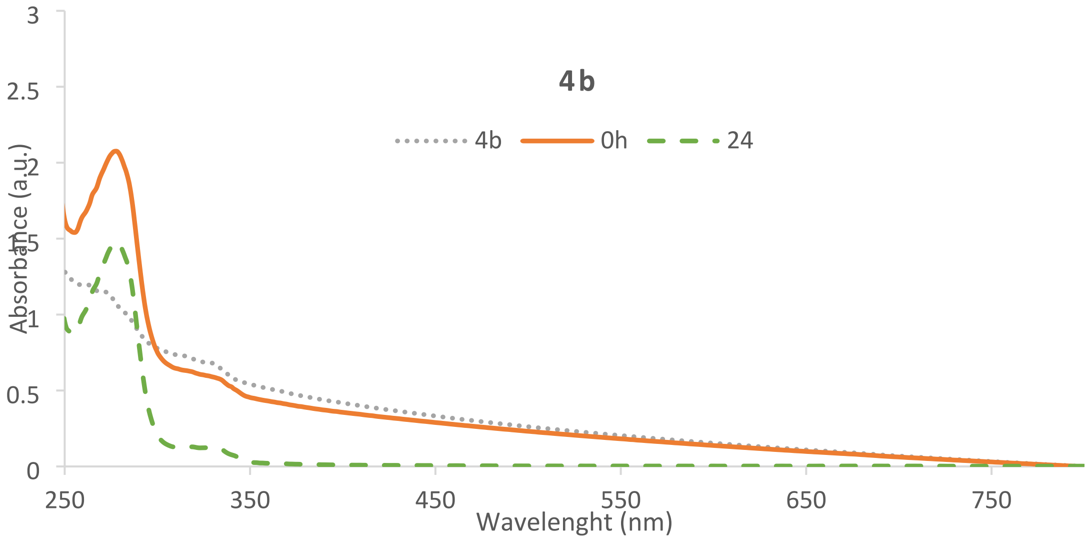

Figure S62. UV-vis spectra of buffered solutions of complex 4b in the presence of BSA in a 1:1 ratio. [complex] = [BSA] = 5x10^{-5} M

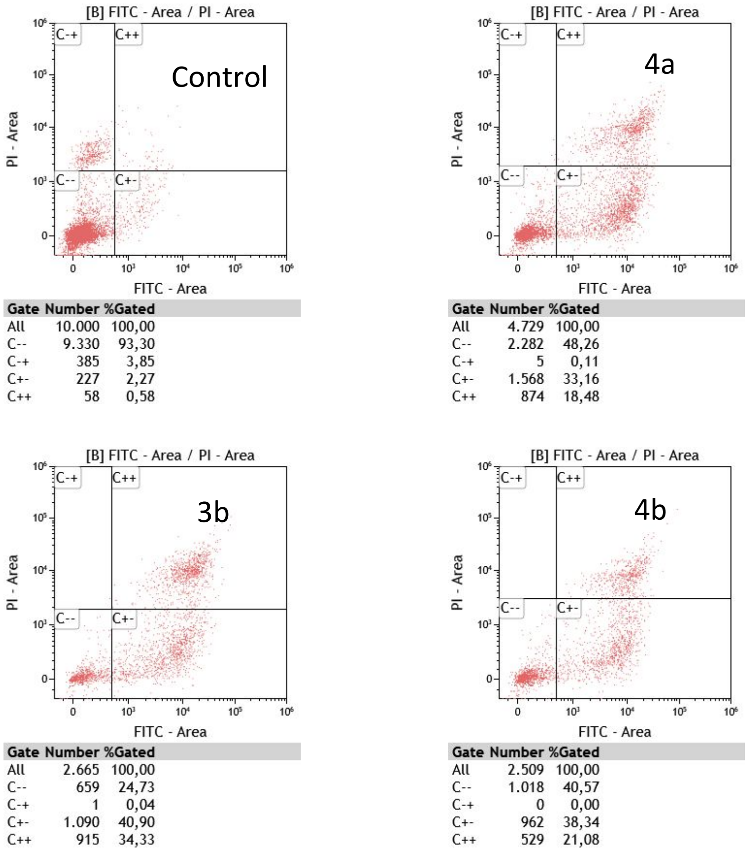

Figure S63. Flow cytometry of Caco-2/TC7cells after incubation for 48 hours with complexes 3b, 4a and 4b Study of cell death mechanism.
